# A catalogue of the tribe Sepidiini Eschscholtz, 1829 (Tenebrionidae, Pimeliinae) of the world

**DOI:** 10.3897/zookeys.844.34241

**Published:** 2019-05-13

**Authors:** Marcin J. Kamiński, Kojun Kanda, Ryan Lumen, Jonah M. Ulmer, Christopher C. Wirth, Patrice Bouchard, Rolf Aalbu, Noël Mal, Aaron D. Smith

**Affiliations:** 1 Museum and Institute of Zoology, Polish Academy of Sciences, Warsaw, Poland Northern Arizona University Flagstaff United States of America; 2 Northern Arizona University, Flagstaff, USA Museum and Institute of Zoology, Polish Academy of Sciences Warsaw Poland; 3 Pennsylvania State University, State College, USA Pennsylvania State University University Park United States of America; 4 Agriculture and Agri-Food Canada, Ottawa, Canada Agriculture and Agri-Food Canada Ottawa Canada; 5 California Academy of Sciences, San Francisco, USA California Academy of Sciences San Francisco United States of America; 6 Royal Belgian Institute of Natural Sciences, Brussels, Belgium Royal Belgian Institute of Natural Sciences Brussels Belgium

**Keywords:** Africa, distribution, Molurini, new synonyms, new combinations, nomen novum, nomenclature, type species

## Abstract

This catalogue includes all valid family-group (six subtribes), genus-group (55 genera, 33 subgenera), and species-group names (1009 species and subspecies) of Sepidiini darkling beetles (Coleoptera: Tenebrionidae: Pimeliinae), and their available synonyms. For each name, the author, year, and page number of the description are provided, with additional information (e.g., type species for genus-group names, author of synonymies for invalid taxa, notes) depending on the taxon rank. Verified distributional records (loci typici and data acquired from revisionary publications) for all the species are gathered. Distribution of the subtribes is illustrated and discussed.

Several new nomenclatural acts are included. The generic names *Phanerotomea* Koch, 1958 [= *Ocnodes* Fåhraeus, 1870] and *Parmularia* Koch, 1955 [= *Psammodes* Kirby, 1819] are new synonyms (valid names in square brackets).

The following new combinations are proposed: *Ocnodesacuductusacuductus* (Ancey, 1883), *O. acuductusufipanus* (Koch, 1952), *O. adamantinus* (Koch, 1952), *O. argenteofasciatus* (Koch, 1953), *O. arnoldiarnoldi* (Koch, 1952), *O. arnoldisabianus* (Koch, 1952), *O.barbosai* (Koch, 1952), *O.basilewskyi* (Koch, 1952), *O.bellmarleyi* (Koch, 1952), *O. benguelensis* (Koch, 1952), *O. bertolonii* (Guérin-Méneville, 1844), *O. blandus* (Koch, 1952), *O. brevicornis* (Haag-Rutenberg, 1875), *O. brunnescensbrunnescens* (Haag-Rutenberg, 1871), *O. brunnescensmolestus* (Haag-Rutenberg, 1875), *O. buccinator* (Koch, 1952), *O. bushmanicus* (Koch, 1952), *O. carbonarius* (Gerstaecker, 1854), *O. cardiopterus* (Fairmaire, 1888), *O. cataractus* (Koch, 1952), *O. cinerarius* (Koch, 1952), *O. complanatus* (Koch, 1952), *O. confertus* (Koch, 1952), *O. congruens* (Péringuey, 1899), *O. cordiventris* (Haag-Rutenberg, 1871), *O. crocodilinus* (Koch, 1952), *O. dimorphus* (Koch, 1952), *O. distinctus* (Haag-Rutenberg, 1871), *O. dolosus* (Péringuey, 1899), *O. dorsocostatus* (Gebien, 1910), *O. dubiosus* (Péringuey, 1899), *O. ejectus* (Koch, 1952), *O. epronoticus* (Koch, 1952), *O. erichsoni* (Haag-Rutenberg, 1871), *O. ferreiraeferreirae* (Koch, 1952), *O. ferreiraezulu* (Koch, 1952), *O. fettingi* (Haag-Rutenberg, 1875), *O. fistucans* (Koch, 1952), *O. fraternus* (Haag-Rutenberg, 1875), *O. freyi* (Koch, 1952), *O. freudei* (Koch, 1952), *O. fulgidus* (Koch, 1952), *O. funestus* (Haag-Rutenberg, 1871), *O. gemmeulus* (Koch, 1952), *O. gibberosulus* (Péringuey, 1908), *O. gibbus* (Haag-Rutenberg, 1879), *O. globosus* (Haag-Rutenberg, 1871), *O. granisterna* (Koch, 1952), *O. granulosicollis* (Haag-Rutenberg, 1871), *O.gridellii* (Koch, 1960), *O. gueriniguerini* (Haag-Rutenberg, 1871), *O. guerinilawrencii* (Koch, 1954), *O. guerinimancus* (Koch 1954), *O. haemorrhoidalishaemorrhoidalis* (Koch, 1952), *O. haemorrhoidalissalubris* (Koch, 1952), *O. heydeni* (Haag-Rutenberg, 1871), *O. humeralis* (Haag-Rutenberg, 1871), *O. humerangula* (Koch, 1952), *O. imbricatus* (Koch, 1952), *O.imitatorimitator* (Péringuey, 1899), *O. imitatorinvadens* (Koch, 1952), *O. inflatus* (Koch, 1952), *O. janssensi* (Koch, 1952), *O. javeti* (Haag-Rutenberg, 1871), *O. junodi* (Péringuey, 1899), *O. kulzeri* (Koch, 1952), *O. lacustris* (Koch, 1952), *O. laevigatus* (Olivier, 1795), *O. lanceolatus* (Koch, 1953), *O. licitus* (Peringey, 1899), *O. luctuosus* (Haag-Rutenberg, 1871), *O. luxurosus* (Koch, 1952), *O. maputoensis* (Koch, 1952), *O. marginicollis* (Koch, 1952), *O. martinsi* (Koch, 1952), *O. melleus* (Koch, 1952), *O. mendicusestermanni* (Koch, 1952), *O. mendicusmendicus* (Péringuey, 1899), *O. miles* (Péringuey, 1908), *O. mimeticus* (Koch, 1952), *O. misolampoides* (Fairmaire, 1888), *O. mixtus* (Haag-Rutenberg, 1871), *O. monacha* (Koch, 1952), *O. montanus* (Koch, 1952), *O. mozambicus* (Koch, 1952), *O. muliebriscurtus* (Koch, 1952), *O. muliebrismuliebris* (Koch, 1952), *O. muliebrissilvestris* (Koch, 1952), *O. nervosus* (Haag-Rutenberg, 1871), *O.notatum* (Thunberg, 1787), *O. notaticollis* (Koch, 1952), *O. odorans* (Koch, 1952), *O. opacus* (Solier, 1843), *O. osbecki* (Billberg, 1815), *O. overlaeti* (Koch, 1952), *O. ovulus* (Haag-Rutenberg, 1871), *O. pachysomaornata* (Koch, 1952), *O. pachysomapachysoma* (Péringuey, 1892), *O. papillosus* (Koch, 1952), *O. pedator* (Fairmaire, 1888), *O. perlucidus* (Koch, 1952), *O. planus* (Koch, 1952), *O. pretorianus* (Koch, 1952), *O. procursus* (Péringuey, 1899), *O. protectus* (Koch, 1952), *O. punctatissimus* (Koch, 1952), *O. puncticollis* (Koch, 1952), *O. punctipennisplanisculptus* (Koch, 1952), *O. punctipennispunctipennis* (Harold, 1878), *O. punctipleura* (Koch, 1952), *O. rhodesianus* (Koch, 1952), *O. roriferus* (Koch, 1952), *O. rufipes* (Harold, 1878), *O. saltuarius* (Koch, 1952), *O.scabricollis* (Gerstaecker, 1854), *O. scopulipes* (Koch, 1952), *O. scrobicollisgriqua* (Koch, 1952), *O. scrobicollissimulans* (Koch, 1952), *O. semirasus* (Koch, 1952), *O. semiscabrum* (Haag-Rutenberg, 1871), *O. sericicollis* (Koch, 1952), *O.similis* (Péringuey, 1899), *O. sjoestedti* (Gebien, 1910), *O. spatulipes* (Koch, 1952), *O. specularis* (Péringuey, 1899), *O. spinigerus* (Koch, 1952), *O. stevensoni* (Koch, 1952), *O. tarsocnoides* (Koch, 1952), *O. temulentus* (Koch, 1952), *O. tenebrosusmelanarius* (Haag-Rutenberg, 1871), *O. tenebrosustenebrosus* (Erichson, 1843), *O. tibialis* (Haag-Rutenberg, 1871), *O. torosus* (Koch, 1952), *O. transversicollis* (Haag-Rutenberg, 1879), *O. tumidus* (Haag-Rutenberg, 1871), *O. umvumanus* (Koch, 1952), *O. vagus* (Péringuey, 1899), *O. vaticinus* (Péringuey, 1899), *O. verecundus* (Péringuey, 1899), *O. vetustus* (Koch, 1952), *O. vexator* (Péringuey, 1899), *O. virago* (Koch, 1952), *O. warmeloi* (Koch, 1953), *O. zanzibaricus* (Haag-Rutenberg, 1875), *Psammophanesantinorii* (Gridelli, 1939), and *P.mirei* (Pierre, 1979).

The type species [placed in square brackets] of the following genus-group taxa are designated for the first time, *Ocnodes* Fåhraeus, 1870 [*Ocnodesscrobicollis* Fåhraeus, 1870], *Psammodophysis* Péringuey, 1899 [*Psammodophysisprobes* Péringuey, 1899], and *Trachynotidus* Péringuey, 1899 [*Psammodesthoreyi* Haag-Rutenberg, 1871].

A lectotype is designated for *Histrionotusomercooperi* Koch, 1955 in order to fix its taxonomic status. *Ulamus* Kamiński is introduced here as a replacement name for *Echinotus* Marwick, 1935 [**Type species.***Aviculaechinata* Smith, 1817] (Mollusca: Pteriidae) to avoid homonymy with *Echinotus* Solier, 1843 (Coleoptera: Tenebrionidae).

## Introduction

The Sepidiini Eschscholtz, 1829 are a diverse tribe of ground-dwelling darkling beetles (Tenebrionidae) of the subfamily Pimeliinae Latreille, 1802 (Figs 1–45). The tribe is widely distributed throughout the Afrotropical Realm, with several species reaching the southern part of the Western Palaearctic (Koch 1955). Some Sepidiini (mainly *Ocnodes* Fåhraeus, 1870 and *Psammodes* Kirby, 1819) are commonly known for their tapping behaviour (sexual communication), which accounts for their vernacular name, the “toktokkies” (Lighton 1987, Matthews et al. 2010). The group also includes many large and/or morphologically remarkable species, e.g., *Stridulomussulcicollis* (Péringuey, 1885), the largest (~ 80.0 mm) currently known tenebrionid species (Koch 1955, Matthews et al. 2010).

**Figures 1–45.**
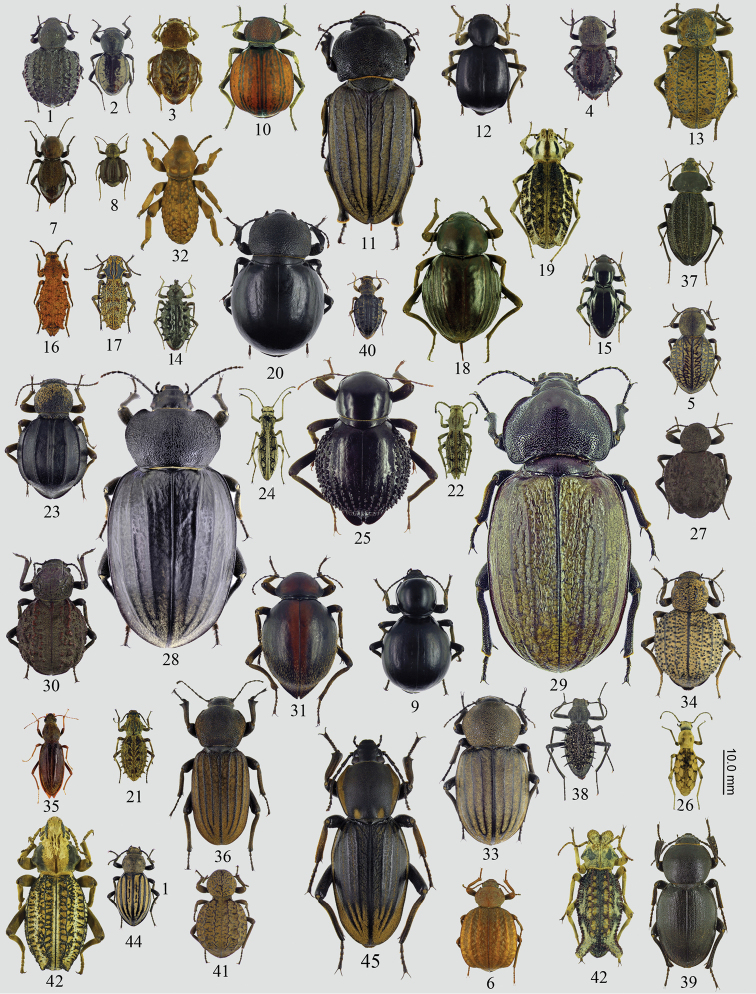
Morphology of representative species of the different subtribes of Sepidiini**1***Arturiumdallastai* (Molurina) **2***Psammophanesjockli* (Molurina) **3***Phrynocolusdentatus* (Molurina) **4***Euphrynussexdentatus* (Molurina) **5***Psammophanesraffrayi* (Molurina) **6***Distretusmormocyle* (Molurina) **7***Psammophanespoccilator* (Molurina) **8***Euphrynussexdentatus* (Molurina) **9***Molurisnitida* (Molurina) **10***Dichthainflata* (Molurina) **11***Psammorhyssustitanus* (Phanerotomeina) **12***Dichthacubica* (Molurina) **13***Brachyphrynuspetrosus* (Molurina) **14***Echinotusspinicollis* (Sepidiina) **15***Psammodesattenuata* (Phanerotomeina) **16***Vietamuscosa* (Sepidiina) **17***Sepidiumbidentatum* (Sepidiina) **18***Psammodesponderosus* (Phanerotomeina) **19***Sepidiumcristatum* (Sepidiina) **20***Ocnodesscabricollis* (Phanerotomeina) **21***Sepidiumhystryx* (Sepidiina) **22***Vietaspiculosa* (Sepidiina) **23***Amiantuspicteti* (Molurina) **24***Sepidiostenuscompressus* (Sepidiina) **25***Psammodesvialis* (Phanerotomeina) **26***Dimoniacisjacksoni* (Sepidiina) **27***Physophrynusharoldi* (Molurina) **28***Tarsocnodesmolossa* (Phanerotomeina) **29***Tarsocnodesnielseni* (Phanerotomeina) **30***Physophrynusbredoi* (Molurina) **31***Psammodesfartus* (Phanerotomeina) **32***Vietomorphafoveipennis* (Sepidiina) **33***Ocnodes* sp. (Phanerotomeina) **34***Physophrynusburdoi* (Molurina) **35***Oxurasetosa* (Oxurina) **36***Ocnodes* sp. (Phanerotomeina) **37***Somaticusaeneus* (Trachynotina) **38***Trachynotusomercooperi* (Trachynotina) **39***Ocnodesprocrustes* (Phanerotomeina) **40***Peringueyiadispar* (Sepidiina) **41***Amiantusgibbosus* (Molurina) **42***Sepidiumcrassicaudatum* (Sepidiina) **43***Sepidiostenusruspolii* (Sepidiina) **44***Psammophanes* sp. (Molurina) **45***Ocnodesguerini* (Phanerotomeina).

**Figures 46–49.**
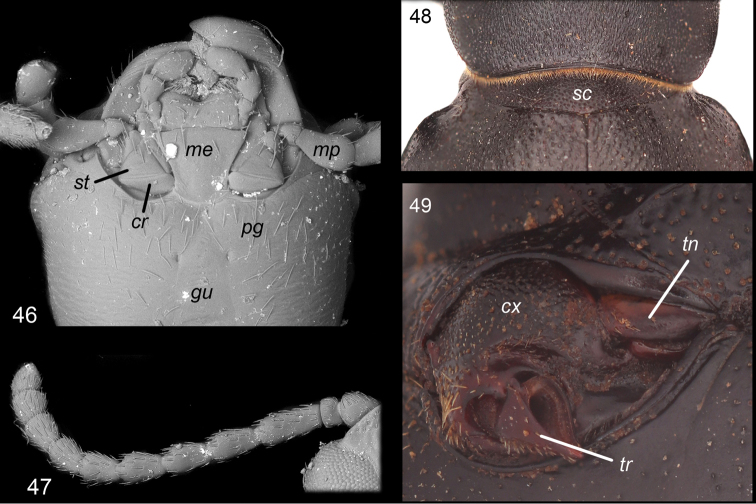
Characters proposed to define Sepidiini. **46** Ventral portion of head (*Dichthainflata*) **47** 11-segmented antenna (*Ocnodessimilis*) **48** connection of pronotum and elytra (*Ocnodesprocrustes*) **49** mesocoxa (*Trachynotidus* sp.). Abbreviations: cr - cardo; cx - coxa; gu - gula; me - mentum; mp - maxillary palp; pg - postgena; sc - scutellum; st - stipes; tn - trochantin; tr - trochanter.

From the morphological perspective, Sepidiini are defined by the following combination of characters (Koch 1955):

- cardo and stipes of maxillae and prelabium not covered by mentum (Fig. 46),
- anterior margin of postgenae with a maxillary ridge or emargination (Fig. 46),
- antennae with eleven segments (Fig. 47),
- mesocoxae, in vast majority of cases, with visible trochantin (reduced in Sepidiina and a few Molurina Solier, 1843) (Fig. 48),
- large scutellum, extending across entire width of mesothoracic peduncle (Fig. 49), and
- elytral base without vertical articulation face (the pronotum consequently freely movable on scutellum). Doyen (1994) also noted that in many Sepidiini the abdominal-sternal interlocking mechanism is different from all other Pimeliinae with the epipleural edge of the elytron overlapping the expanded sternite edge, rather than dovetailing into a groove.

The last comprehensive checklist of the species currently classified within Sepidiini was published by Gebien (1937a). At that time, 581 species were listed and divided over two separate tribes, Molurini and Sepidiini. No subtribal classification was proposed. After Gebien’s catalogue (1937a) more than 50 contributions were published on the taxonomy, nomenclature, and classification of today’s Sepidiini (see references). This includes descriptions of more than 400 species, reinterpretation of extremely diverse genera (e.g., *Ocnodes*, *Psammodes*, *Somaticus* Hope, 1840), fusion of the former tribes Molurini and Sepidiini (see Koch 1955, Doyen 1994), and finally, designation of the six currently recognised subtribes (see Bouchard et al. 2005, 2011).

From the strictly formal point of view, the validity of many names introduced after 1937 remained questionable (e.g., *Histrionotusomercooperi* Koch, 1955), since their unusual “descriptions” were incorporated in remarks concerning other taxa. Furthermore, the taxonomic affiliation of many genera and species is uncertain because of ambiguous remarks made by the contributors, see notes in the catalogue below.

The main aim of this work is to synthesise available nomenclatural, taxonomic, and distributional information concerning Sepidiini.

## Materials and methods

### Nomenclatural data

All nomenclaturally available family-, genus-, and species-group names are included. The author, year, and page of the original description are provided for each scientific name. The type genus for each family-group name and the type species and type fixation for each genus-group name are included. Type species fixed by original designation were only accepted when an explicit statement (e.g., “**Type species.**”) was used in the original publication (see ICZN 1999, Articles 67.5, 68.2). The reference in which a given generic or specific name is first placed in synonymy with the current valid name is listed [e.g., “syn. by Penrith (1986: 11)”]. For every species-group name which was subsequently transferred to another genus, the original genus-group name is provided. The author which transferred a particular species-group name to a currently accepted subgenus is listed in square parentheses (e.g., *vagecostatus* (Fairmaire, 1882b) *Psammodes* [Koch 1953a] listed under Psammophanes (Psammophanes)): in this example, Koch (1953a) was the one to include this species under the subgenus Psammophanes. The subtribal classification follows Bouchard et al. (2011).

Type deposition data and the status of the name bearing types was primarily taken from the original publications. However, when authors provided the name of the entomological collection for the type deposition without referring to any public institutions the following publications were used in order to locate those collections:

**Allard, Bates, Crotch, DeGeer, Desbrochers des Loges, Dohrn, Dupont, Gory, Hope, Klug, Pallas, Pascoe, Sahlberg, and Westwood collections** – Bousquet (2016)

**Banks collection** – Chambers (2000)

**Fabricius collection** – Zimsen (1964), Copenhagen Museum (2019)

**Gestro collection** – Conci and Poggi (1996)

**Haag-Rutenberg collection** – Scherer (1992), Bousquet (2016)

**Kocher collection** – Bezděk and Regalin (2015)

**Kolbe collection** – Ohl (2012)

**Spinola collection** – Ekis (1975), Generani and Scaramozzino (2000), and Bousquet (2016)

Type deposition information provided in this catalogue was verified against the databases originating from the Basel (Kulzer 1963), British, Brussels, Budapest (Merkl et al. 2015), Cape, Ditsong, Tervuren, Warsaw, and Windhoek (Irish 1985) museums.

The following format for deposition information was used throughout the catalogue:

**Agricultural Institute** Agricultural Institute in Pretoria, Pretoria, South Africa

**Barcelona Museum** Museu de Ciències Naturals de Barcelona, Barcelona, Spain

**Basel Museum** Naturhistorisches Museum Basel, Basel, Switzerland

**Berlin Museum** Museum für Naturkunde, Berlin, Germany

**Bloemfontein Museum** Bloemfontein National Museum, Bloemfontein, South Africa

**Bologna Museum** Bologna Zoological Museum, Bologna, Italy

**Bremen Museum** Übersee Museum, Bremen, Germany

**British Museum** The Natural History Museum, London, United Kingdom

**Brussels Museum** Muséum des sciences naturelles de Belgique, Brussels, Belgium

**Budapest Museum** Magyar Természettudományi Múzeum, Budapest, Hungary

**California Academy** Museum of the California Academy of Sciences, San Francisco, USA

**Cambridge Museum** Harvard Museum of Natural History, Cambridge, USA

**Cape Museum** Iziko South African Museum, Cape Town, South Africa

**Companhia Diamantes** Companhia de Diamantes de Angola, Luanda, Angola

**Copenhagen Museum** Statens Naturhistoriske Museum, University of Copenhagen, Copenhagen, Denmark

**Cornell University** Cornell University, Ithaca, USA

**Ditsong Museum** Ditsong National Museum of Natural History, Pretoria, South Africa

**Dundo Museum** Dundo Museum, Dundo, Angola

**Durban Museum** Durban Natural Science Museum, Durban, South Africa

**Florence Museum** Museo di Storia Naturale di Firenze, Florence, Italy

**Frankfurt Museum** Naturmuseum Senckenberg, Frankfurt, Germany

**Geneva Museum** Muséum d’Histoire Naturelle, Geneva, Switzerland

**Genoa Museum** Civic Museum of Natural History Giacomo Doria, Genoa, Italy

**Glasgow Museum** Hunterian Zoology Museum, Glasgow, Scotland

**Hamburg University** Universität Hamburg, Hamburg, Germany

**Humboldt University** Humboldt-Universität, Berlin, Germany

**Kenya Museum** National Museums of Kenya, Nairobi, Kenia

**Kiel Museum** Zoologischen Museum Kiel, Kiel, Germany

**Leiden Museum** Naturalis Museum, Leiden, Holland

**Lund University** Zoological Museum, Lund University, Lund, Sweden

**Madrid Museum** Museo Nacional de Ciencias Naturales, Madrid, Spain

**Maputo Museum** Centro de Investigação Científica Algodoeira, Maputo, Mozambique

**Marseille Museum** Muséum d’histoire naturelle de Marseille, Marseille, France

**McGregor Museum** McGregor Museum, Kimberley, South Africa

**Milan Museum** Museo Civico di Storia Naturale, Milano, Italy

**Monaco Museum** Nouveau Musée National de Monaco, Monaco, Monaco

**Munich Museum** Bayerisches Nationalmuseum, Munich, Germany

**Museo Civico Filangieri** Museo Civico Filangieri, Naples, Italy

**National Congo** nstitute of the National Parks of Belgian Congo (temporarily preserved in Tervuren Museum)

**Naturhistoriska riksmuseet** Naturhistoriska riksmuseet, Stockholm, Sweden

**New York Museum** American Museum New York, New York, USA

**Ohio State** Ohio State University, Columbus, USA

**Oxford University** Oxford University Museum of Natural History, Oxford, United Kingdom

**Paris Museum** Muséum National d’Histoire Naturelle, Paris, France

**Prague Museum** Národní muzeum, Prague, Czech Republic

**Pretoria University** University of Pretoria, Pretoria, South Africa

**Rabat Institute** d’Entomologie de l’Institut Scientifque Chérifen, Rabat, Morocco

**Rhodes University** Rhodes University, Grahamstown, South Africa

**Rhodesia Museum** National Museum of Southern Rhodesia, Bulawayo, Zimbabwe

**South African National**

**Collection** South African National Collection of Insects, Pretoria, South Africa

**Stellenbosch University** Stellenbosch University, Stellenbosch, South Africa

**Stuttgart Museum** Staatliches Museum für Naturkunde Stuttgart, Stuttgart, Germany

**Tervuren Museum** Musée royal de l’Afrique centrale, Tervuren, Belgium

**Torino Museum** Museo Regionale di Scienze Naturali di Torino, Turin, Italy

**Trieste Museum** Museo Civico di Storia Naturale, Trieste, Italy

**Uppsala University** Uppsala universitet Evolutionsmuseet, Uppsala, Sweden

**Vienna Museum** Naturhistorisches Museum Wien, Wien, Austria

**Warsaw Museum** Muzeum i Instytut Zoologii, Polska Akademia Nauk, Warsaw, Poland

**Windhoek Museum** National Museum of Namibia, Windhoek, Namibia

### Distribution data

The distribution of all subtribes was illustrated using Quantum GIS (QGIS) v. 2.4. All vector layers were downloaded from the Natural Earth webpage (http://www.naturalearthdata.com). The list of localities was built by consulting available literature and is available as an Suppl. material to this publication (Suppl. material 1). Because of the uncertain status of most of the listed species, and extreme difficulties with identification of the majority of Sepidiini representatives, only records acquired from original species descriptions or revisionary papers were included. Geographic data with low degrees of accuracy (e.g., countries or states) were not georeferenced, and therefore are absent on the maps and distributional sections of particular subtribes.

## Results and remarks

**Figure 50.**
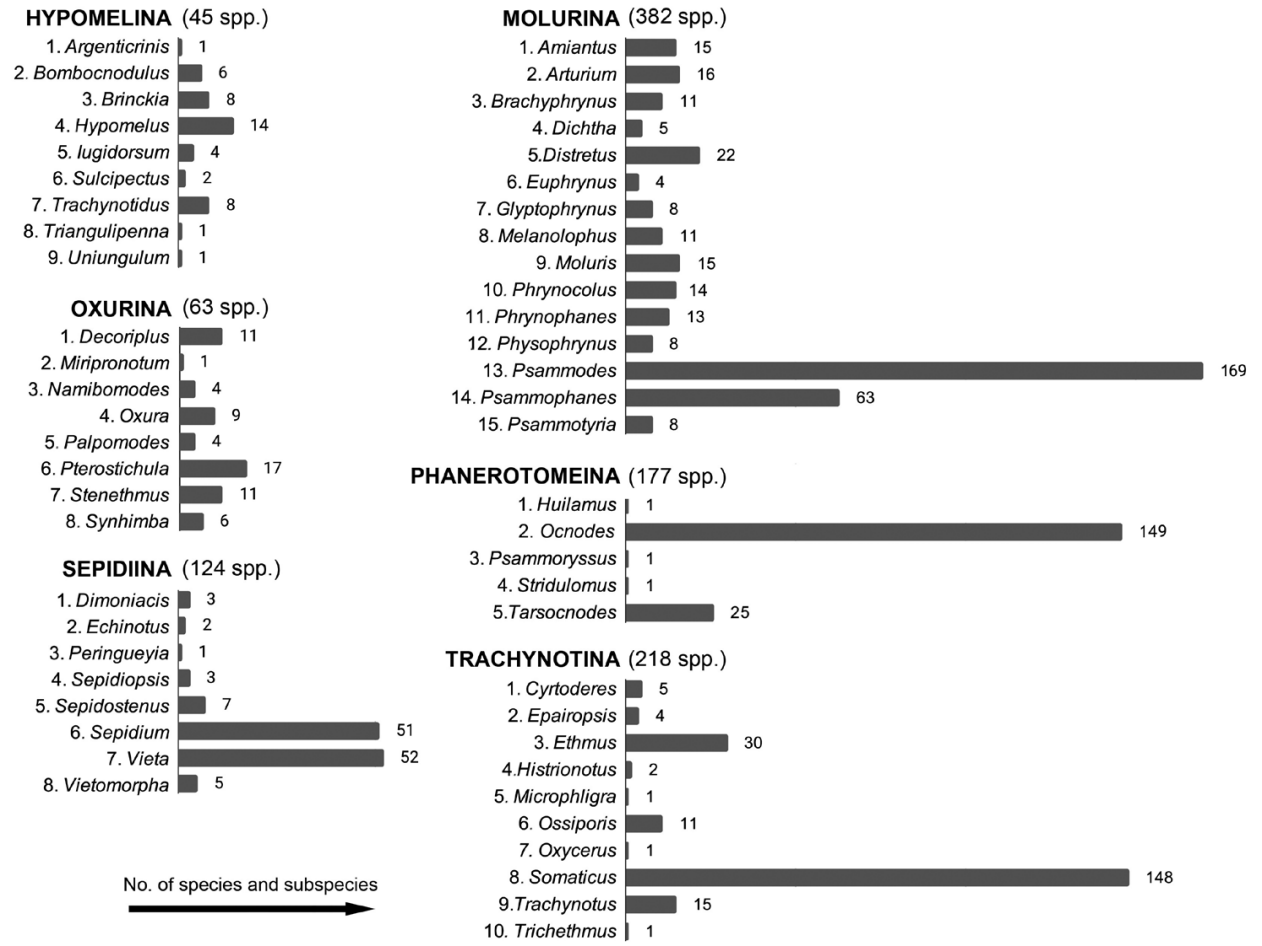
Summary of the generic and sub-/species diversity of the subtribes representing Sepidiini.

A total of 1009 valid species and subspecies divided over 55 genera (33 subgenera) and six subtribes is listed in this catalogue. The subtribe Molurina is the most diverse with 382 valid species-group taxa, followed by the Trachynotina (218), Phanerotomeina (177), Sepidiina (124), Oxurina (63), and Hypomelina (45). In most cases, the species and subspecies diversity is not equally divided over the available genera (Fig. 50). This is most evident in the case of Phanerotomeina, where a single genus, *Ocnodes*, groups over 83% of currently accepted species and subspecies. A similar trend is seen in Molurina and Trachynotina, while in Sepidiina over 80% of known species and subspecies diversity is divided between *Sepidium* Fabricius, 1775 and *Vieta* Hope, 1840 (Fig. 50). This tendency was not reported for the most recently revised subtribes (Louw 1979, Penrith 1986), i.e., Hypomelina and Oxurina. In total, 11 monotypic genera are listed.

Although this publication focuses on the nomenclature and classification of Sepidiini, the examined references enabled to reveal the most urgent taxonomic problems within the tribe. According to Penrith (1986, 1987), the status of the majority of currently recognised subtribes should be tested. This strictly relies on the verification of monophyly of the most speciose genera, such as *Ocnodes* and *Psammodes*. The taxonomic history of theses taxa is complex (see catalogue below), resulting in taxonomic ambiguities at the higher classification levels. The other urgent taxonomic problem within Sepidiini concerns the verification of the status of many genera of Molurina. The available contributions to the taxonomy of molurines presented in several different publications (e.g., Gebien 1910a, Wilke 1921, Koch 1951, 1952, 1953b, 1954a, 1956, 1960, 1962a). The lack of a comprehensive revisions may cause taxonomic inflation, especially when alphataxonomic contributions prevail. Future efforts concerning Sepidiini should include phylogenetic and revisionary studies.

**Figure 51.**
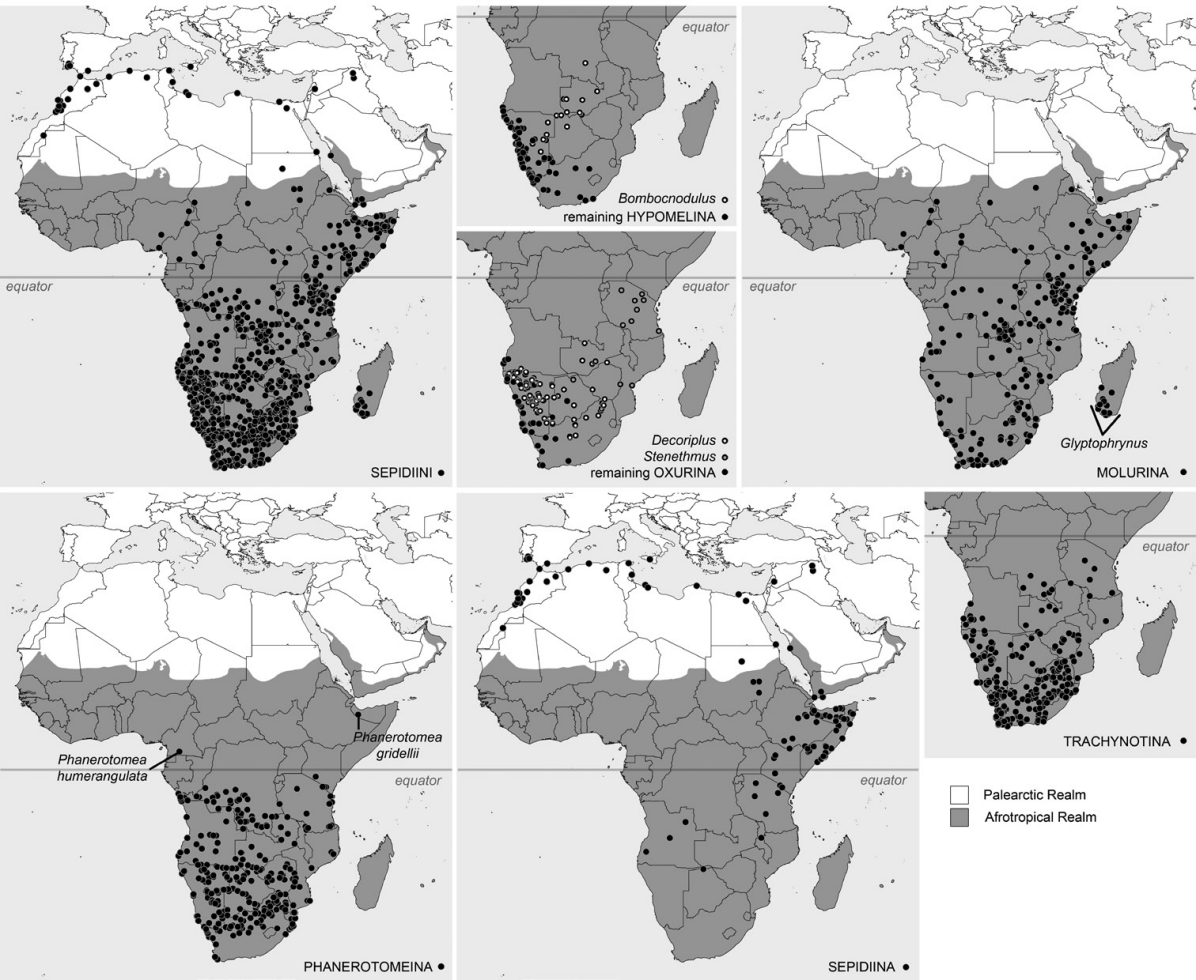
Revealed distributional patterns of Sepidiini. Selected outstanding records for particular subtribes were highlighted. Images were generated based on 1,666 records acquired from descriptive and revisionary papers.

A database containing 2523 distributional records (857 not georeferenced) was created during this study (Fig. 51, Suppl. material 1). It needs to be highlighted that this list exclusively concerns loci typici and taxonomically revised data (acquired from generic revisions). However, because most Sepidiini species are known only from the type series, some basic remarks concerning distributional patterns of this tribe might be made.

According to the acquired data, Sepidiini are widely distributed throughout the Afrotropical Realm, except its northwestern parts (Fig. 51). Only Sepidiina has a distribution that extends into the Western Palaearctic (mainly Mediterranean Basin). However, the majority of the species of this subtribe were described from Somalia. Within the remaining subtribes, the presence of only Molurina and Phanerotomeina was revealed north of the equator. The former seems to be especially speciose in the Horn of Africa. Furthermore, Molurina is the only subtribe within Sepidiini with Malagasy representatives. The distribution of Hypomelina, Oxurina, and Trachynotina is limited to the southern part of the African continent. The majority of the species representing the Hypomelina were described from the Namibian coast (Fig. 51).

## Catalogue of the Sepidiini (Tenebrionidae: Pimeliinae) of the world

### Tribe Sepidiini Eschscholtz, 1829: 4

**Type genus.***Sepidium* Fabricius, 1775

**Subtribes included.**Hypomelina, Molurina, Oxurina, Phanerotomeina, Sepidiina, Trachynotina.

**Distribution.** Western Palaearctic (mainly Mediterranean Basin) and Afrotropical Realm (with exception of western Africa) (Fig. 51).

#### Subtribe Hypomelina Koch, 1955: 36

**Type genus.***Hypomelus* Solier, 1843

**Taxonomic diversity.** (9 gen., 45 spp.): *Argenticrinis* (1 sp.), *Bombocnodulus* (6), *Brinckia* (8), *Hypomelus* (14), *Iugidorsum* (4), *Sulcipectus* (2), *Trachynotidus* (8), *Triangulipenna* (1), *Uniungulum* (1).

**Distribution.** Southern part of the Afrotropical Realm. Majority of species were described from the Namibian coast. A single genus, *Bombocnodulus*, reaching north to Central Africa (Fig. 51).

##### Genus *Argenticrinis* Louw, 1979: 100

**Type species.***Argenticrinishaackei* Louw, 1979 (by original designation); syn. of *Psammodeslossowi* Koch, 1952)

*lossowi* (Koch, 1952: 339) *Psammodes* [Louw, 1980: 216]

**Type data.** Holotype (Ditsong Museum)

= *Argenticrinishaackei* Louw, 1979: 101 [syn. by Louw (1980: 216)]

**Type data.** Holotype (Windhoek Museum) and paratypes (Ditsong Museum, Windhoek Museum)

##### Genus *Bombocnodulus* Koch, 1955: 36

**Type species.***Psammodescrinicollis* Haag-Rutenberg, 1879 (by monotypy)

**Notes.** Although the description is unusual, it meets the criteria of Art. 11 and 13 ICZN (1999).

Genus originally described under Phanerotomeina, and subsequently transferred to Hypomelina by Louw (1979).

*crinicolliscrinicollis* (Haag-Rutenberg, 1879: 293) *Psammodes* [Koch, 1955: 36]

**Type data.** Holotype (British Museum)

= *Psammodesinquinatus* Péringuey, 1899: 292 [syn. by Penrith (1986: 59)]

**Type data.** Holotype (Cape Museum)

*crinicollisfortuitus* (Péringuey, 1899: 292) *Psammodes* [Penrith, 1986: 61]

**Type data.** Syntypes (Cape Museum)

**Notes.**Penrith (1986) refers to a single specimen; however, the original description is based on more than one specimen. Péringuey (1899) does not designate a holotype in the text.

*dollmani* Penrith, 1986: 64

**Type data.** Holotype (British Museum) and paratype (Ditsong Museum)

*longantennatus* Penrith, 1986: 62

**Type data.** Holotype (Ditsong Museum) and paratype (Windhoek Museum)

*torridus* Penrith, 1986: 62

**Type data.** Holotype (British Museum) and paratypes (British Museum, Ditsong Museum)

*wittei* Penrith, 1986: 66

**Type data.** Holotype (Brussels Museum) and paratypes (Brussels Museum, Ditsong Museum)

##### Genus *Brinckia* Koch, 1962b: 117

**Type species.***Psammodesdebilis* Péringuey, 1899 (by original designation)

*australis* Penrith, 1986: 81

**Type data.** Holotype (Ditsong Museum) and paratypes (Ditsong Museum, Windhoek Museum)

*debilis* (Péringuey, 1899: 277) *Psammodes* [Koch, 1962b: 121]

**Type data.** Lectotype, designated by Penrith (1986) (Cape Museum) and paralectotypes (Ditsong Museum)

*delicata* Penrith, 1986: 77

**Type data.** Holotype (Windhoek Museum) and paratypes (Ditsong Museum, Windhoek Museum)

*insularis* (Péringuey, 1908: 410) *Trachynotidus* [Koch, 1962b: 121]

**Type data.** Lectotype, designated by Penrith (1986) (Ditsong Museum) and paralectotype (Cape Museum)

*oblonga* (Thunberg, 1787: 37) *Sepidium* [Ferrer, 2009: 114]

**Type data.** Holotype (Uppsala University)

*oograbiensis* Koch, 1962b: 118

**Type data.** Holotype (Ditsong Museum) and paratype (Cape Museum)

*serratina* Koch, 1962b: 119

**Type data.** Holotype (Ditsong Museum) and paratypes (Basel Museum, Ditsong Museum)

*vaga* (Péringuey, 1908: 411) *Trachynotidus* [Koch, 1962b: 122]

**Type data.** Lectotype, designated by Penrith (1986) (Cape Museum) and paralectotypes (Ditsong Museum, Windhoek Museum)

##### Genus *Hypomelus* Solier, 1843: 93

**Type species.***Hypomelusbicolor* Solier, 1843 (by original designation); syn. of *Helopsperonatus* Germar, 1823

**Notes.** Interpreted as a subgenus of *Psammodes* for a long time (e.g., Gebien 1937a) before being re-elevated to the generic level by Koch (1955).

The species composition mostly follows that in Gebien’s catalogue (1937a).

*basalis* (Haag-Rutenberg, 1871a: 70, in key) *Psammodes* [Gebien, 1937a: 772]

**Type data.** Holotype (Munich Museum – Haag-Rutenberg coll.)

**Note.** A detailed morphological description was provided by Haag-Rutenberg (1871b: 93).

*inaequalis* Solier, 1843: 98

**Type data.** Holotype (Torino Museum – Spinola coll.)

= *Hypomelusflagrans* Péringuey, 1899: 273 [homonym of *Psammodesflagrans* Péringuey, 1899: 295 published on the same date; Gebien 1910b acted as First Reviser when he proposed the replacement name *Psammodesdentipennis* for the species *Hypomelusflagrans* Péringuey, 1899: 273]

= *Psammodesdentipennis*Gebien 1910b: 155, replacement name [syn. by Gebien (1937a: 772)]

**Type data.** Holotype (Cape Museum)

*interstitialis* (Haag-Rutenberg, 1871a: 70, in key) *Psammodes* [Gebien, 1937a: 772]

**Type data.** Holotype (Munich Museum – Haag-Rutenberg coll.)

**Note.** A detailed morphological description was provided by Haag-Rutenberg (1871b: 94).

*obliquatus* Solier, 1843: 97

**Type data.** Holotype (Geneva Museum – Gory collection)

= *Hypomelussabulosus* Solier, 1843: 308 [syn. by Haag-Rutenberg (1871b: 97)]

**Type data.** Syntypes (Paris Museum)

*obliteratus* Solier, 1843: 96

**Type data.** Holotype (Geneva Museum – Gory collection)

*peringueyi* (Gebien 1910b: 158, replacement name) *Psammodes* [Gebien, 1937a: 772]

= *Hypomelusplausibilis* Péringuey, 1899: 295 [homonym of *Psammodesplausibilis* Péringuey, 1899: 271 published on the same date; Gebien 1910b acted as First Reviser when he proposed the replacement name *Psammodesperingueyi* for the species *Hypomelusplausibilis* Péringuey, 1899: 295]

**Type data.** Syntypes (Cape Museum)

*peronatus* (Germar, 1823: 149) *Helops* [Gebien, 1937a: 771]

**Type data.** Syntypes (Cape Museum, Paris Museum)

= *Oxurapsammodioides* Guérin-Méneville, 1834: 20 [syn. by Haag-Rutenberg (1871b: 91)]

**Type data.** Syntypes (Paris Museum)

= *Hypomelusbicolor* Solier, 1843: 100 [syn. by Haag-Rutenberg (1871b: 91)]

**Type data.** Syntypes (Paris Museum)

*profugus* (Péringuey, 1899: 277) *Psammodes* [Koch 1955: caption to fig. 4]

**Type data.** Syntypes (Cape Museum)

*reflexicollis* (Haag-Rutenberg, 1871a: 70, in key) *Psammodes* [Gebien, 1937a: 772]

**Type data.** Holotype (Munich Museum – Haag-Rutenberg coll.)

**Notes.** A detailed morphological description was provided by Haag-Rutenberg (1871b: 97).

*reflexus* (Haag-Rutenberg, 1871a: 70, in key) *Psammodes* [Gebien, 1937a: 772]

**Type data.** Holotype (Munich Museum – Haag-Rutenberg coll.)

**Notes.** A detailed morphological description was provided by Haag-Rutenberg (1871b: 95).

*servus* Péringuey, 1899: 294

**Type data.** Syntypes (Cape Museum)

*setosocostatus* (Haag-Rutenberg, 1871a: 70, in key) *Psammodes* [Gebien, 1937a: 772]

**Type data.** Syntypes (Munich Museum – Haag-Rutenberg coll.)

**Notes.** A detailed morphological description was provided by Haag-Rutenberg (1871b: 99).

*villosocostatus* Solier, 1843: 98

**Type data.** Holotype (Torino Museum – Spinola coll.)

*vulpinus* (Haag-Rutenberg, 1873: 45, replacement name) *Psammodes* [Gebien, 1937a: 772]

= *Psammodeshirtipennis* Haag-Rutenberg, 1871b: 92 [junior primary homonym of *Psammodeshirtipennis* Haag-Rutenberg, 1871a: 105]

**Type data.** Holotype (Munich Museum)

##### Genus *Iugidorsum* Louw, 1979: 102

**Type species.***Iugidorsumcumstriis* Louw, 1979 (by original designation)

*cumstriiscumstriis* Louw, 1979: 106

**Type data.** Holotype (Windhoek Museum) and paratypes (Windhoek Museum and Ditsong Museum)

*cumstriismagnum* Louw, 1979: 106

**Type data.** Holotype (Ditsong Museum) and paratype (Windhoek Museum)

*cumstriisprominens* Louw, 1979: 106

**Type data.** Holotype and paratypes (Ditsong Museum)

*sinestriis* Louw, 1979: 107

**Type data.** Holotype and paratype (Windhoek Museum)

##### Genus *Sulcipectus* Louw, 1979: 109

**Type species.***Sulcipectuslevis* Louw, 1979 (by original designation)

*cumcavus* Louw, 1979: 113

**Type data.** Holotype and paratypes (Windhoek Museum)

*levis* Louw, 1979: 110

**Type data.** Holotype and paratypes (Windhoek Museum)

##### Genus *Trachynotidus* Péringuey, 1899: 296

**Type species.***Psammodesthoreyi* Haag-Rutenberg, 1871 (**here designated**)

Péringuey (1899) designated both *Clinocranionalstoni* Péringuey, 1885 and *Psammodesthoreyi* Haag-Rutenberg, 1871 as type species. According to the regulations of ICZN (1999) this cannot be treated as valid fixation of type species. Therefore, to provide nomenclatural stability *Psammodesthoreyi* Haag-Rutenberg, 1871 is hereby designated as a type species of the genus *Trachynotidus*.

**Notes.** In 1904, Péringuey described a new species named *damarinus* under “Gen. *Trachynotideus* Péring”. The spelling “*Trachynotideus*” was generally treated as an incorrect subsequent spelling of *Trachynotidus* by subsequent authors (e.g., Gebien 1910b, 1937a) and is not in prevailing usage. This view is also adopted here. Judging from the context, Péringuey (1904) did not intend to describe “*Trachynotideus*” as a new genus as he stated “genus … nov” or “n. gen.” near the other newly introduced generic names, i.e., “DIESTESOMA, n. gen.”.

Koch (1955) provided a drawing of a species which he referred to as “*Trachynotidus XXI-lineatus*”. However, this description does not meet the criteria of Art. 13.1.1 of the ICZN (1999).

*alstoni* (Péringuey, 1885: 116) *Clinocranion* [Péringuey, 1899: 297]

**Type data.** Holotype (Cape Museum)

*angulicollis* (Haag-Rutenberg, 1871a: 69, in key) *Psammodes* [Péringuey, 1904: 234]

**Type data.** Holotype (Naturhistoriska riksmuseet)

**Notes.** A detailed morphological description was provided by Haag-Rutenberg (1871b: 88).

*cognatus* Péringuey, 1899: 297

**Type data.** Holotype (Cape Museum)

*cruentus* Péringuey, 1908: 411

**Type data.** Syntypes (Cape Museum)

*eximius* Péringuey, 1899: 298

**Type data.** Syntypes (British Museum, Cape Museum)

*gravis* (Gemminger 1870: 122, replacement name) *Psammodes* [Gebien, 1937a: 771]

= *Hypomelusgrandis* Solier, 1843: 101 [homonym of *Phanerotomagrande* Solier, 1843: 90 published on the same date; Gebien 1910b acted as First Reviser when he proposed the replacement name *Psammodesgravis* for the species *Hypomelusgrandis* Solier, 1843: 101]

**Type data.** Holotype (Geneva Museum – Gory collection)

*rufozonatus* (Fairmaire, 1888a: 194) *Trachynotus* [Gebien, 1937a: 771]

**Type data.** Holotype (Paris Museum)

= *Trachynotidusmanifestus* Péringuey, 1899: 297 [syn. by Péringuey (1904: 297)]

**Type data.** Syntypes (Cape Museum)

*thoreyi* (Haag-Rutenberg, 1871b: 104) *Psammodes* [Gebien, 1937a: 771]

**Type data.** Syntypes (Munich Museum – Haag-Rutenberg coll.)

##### Genus *Triangulipenna* Louw, 1979: 114

**Type species.***Triangulipennalacuna* Louw, 1979 (by original designation)

*lacuna* Louw, 1979: 115

**Type data.** Holotype (Cape Museum) and paratypes (Cape Museum, Windhoek Museum)

##### Genus *Uniungulum* Koch, 1962b: 113

**Type species.***Uniungulumhoeschi* Koch, 1962 (by original designation)

*hoeschi* Koch, 1962b: 114

**Type data.** Holotype (Ditsong Museum) and paratypes (Budapest Museum, Ditsong Museum)

#### Subtribe Molurina Solier, 1843: 1

**Type genus.***Moluris* Latreille, 1802

**Taxonomic diversity.** (15 gen., 382 spp.): *Amiantus* (15 sp.), *Arturium* (16), *Brachyphrynus* (11), *Dichtha* (5), *Distretus* (22), *Euphrynus* (4), *Glyptophrynus* (8), *Melanolophus* (11), *Moluris* (15), *Phrynocolus* (14), *Phrynophanes* (13), *Physophrynus* (8), *Psammodes* (169), *Psammophanes* (63), *Psammotyria* (8).

**Distribution.** With exception of western Africa, widely distributed in the Afrotropical Realm. *Glyptophrynus* is the only Malagasy representative of the whole tribe (Fig. 51).

##### Genus *Amiantus* Fåhraeus, 1870: 279

**Type species.***Amiantusgibbosus* Fåhraeus, 1870 (by subsequent designation by Haag-Rutenberg (1871a: 45))

*browni* Haag-Rutenberg, 1871a: 47

**Type data.** Syntypes (British Museum, Munich Museum – Haag-Rutenberg coll.)

*connexus* Haag-Rutenberg, 1871a: 49

**Type data.** Syntypes (British Museum)

*costipennis* Kolbe, 1886: 292

**Type data.** Holotype (Berlin Museum)

*decemcostatus* (Gebien, 1910a: 156) *Phrynocolus* [Gebien 1937a: 757]

**Type data.** Holotype (Hamburg University)

*gibbosus* Fåhraeus, 1870: 280

**Type data.** Syntypes (Naturhistoriska riksmuseet)

*globulipennis* Péringuey, 1896: 167

**Type data.** Holotype (Cape Museum)

= *Amiantusmulticostatus* Fairmaire, 1899a: 181 [syn. by Gebien (1937a: 757)]

**Type data.** Syntypes (Basel Museum, Paris Museum)

*lobicollis* Kolbe, 1886: 291

**Type data.** Holotype (Berlin Museum)

*mechowi* (Quedenfeldt, 1885: 6) *Distretus* [Gebien 1910b: 152]

**Type data.** Syntypes (Berlin Museum)

*octocostatus* Péringuey, 1896: 167

**Type data.** Holotype (Cape Museum)

*octocristatus* Fairmaire, 1899: 181

**Type data.** Holotype (Paris Museum)

*opacus* Haag-Rutenberg, 1871a: 49

**Type data.** Syntypes (British Museum, Munich Museum – Haag-Rutenberg coll.)

*pusillus* Péringuey, 1904: 235

**Type data.** Syntypes (Cape Museum)

*rusticus* Fåhraeus, 1870: 280

**Type data.** Syntypes (Naturhistoriska riksmuseet)

*scrobipennis* Haag-Rutenberg, 1875: 68

**Type data.** Syntypes (British Museum, Munich Museum – Haag-Rutenberg coll.)

*undosus* Distant, 1892: 199

**Type data.** Holotype (Ditsong Museum)

##### Genus *Arturium* Koch, 1951: 83

**Type species.***Melanolophusater* Waterhouse, 1885 (by original designation)

*absciri* Koch, 1959: 7

**Type data.** Holotype (Munich Museum)

*ater* (Waterhouse, 1885: 234) *Melanolophus* [Koch, 1951: 83]

**Type data.** Syntypes (British Museum)

**Notes.**Wilke (1921) suggested synonymy between *Melanolophusater* Waterhouse, 1885 and *M.tenuecostatus* Gebien, 1910. However, this view was not accepted by Gebien (1937b, 1938) or subsequent authors (e.g., Koch (1951)).

*auriculatus* (Gebien, 1910a: 155) *Phrynocolus* [Koch, 1951: 83]

**Type data.** Holotype (Basel Museum)

*benanum* (Wilke, 1921: 167) *Phrynocolus* [Koch, 1951: 83]

**Type data.** Holotype (Basel Museum)

*crispatus* (Fairmaire, 1887: 184) *Phrynocolus* [Koch, 1951: 83]

**Type data.** Syntypes (Paris Museum)

= *Phrynocolusundatocostatus* Kolbe, 1891: 30 [syn. by Wilke (1921: 172)]

**Type data.** Holotype (Berlin Museum)

*dallastai* Ardoin, 1977: 811

**Type data.** Holotype and paratype (Tervuren Museum)

*fiomicum* (Wilke, 1921: 167) *Phrynocolus* [Koch, 1951: 83]

**Type data.** Holotype (Berlin Museum)

*fulleborni* (Wilke, 1921: 167) *Phrynocolus* [Koch, 1951: 83]

**Type data.** Holotype (Berlin Museum)

*gebieni* (Wilke, 1921: 168) *Phrynocolus* [Koch, 1951: 83]

**Type data.** Holotype (Basel Museum)

*glauningi* (Wilke, 1921: 167) *Phrynocolus* [Koch, 1951: 83]

**Type data.** Holotype (Berlin Museum)

*methneri* (Wilke, 1921: 166) *Phrynocolus* [Koch, 1951: 83]

**Type data.** Holotype (Basel Museum)

*parvulus* (Gestro, 1895: 132) *Phrynocolus* [Koch, 1951: 83]

**Type data.** Holotype (Genoa Museum)

*pretiosum* (Wilke, 1921: 167) *Phrynocolus* [Koch, 1951: 83]

**Type data.** Holotype (Basel Museum)

*tenuecostatus* (Gebien, 1910a: 155) *Phrynocolus* [Koch, 1951: 83]

**Type data.** Syntypes (Basel Museum, Berlin Museum)

*undaticostis* (Fairmaire, 1887: 183) *Phrynocolus* [Koch, 1951: 83]

**Type data.** Holotype (Paris Museum)

*wembericum* (Wilke, 1921: 168) *Phrynocolus* [Koch, 1951: 83]

**Type data.** Holotype (Berlin Museum)

##### Genus *Brachyphrynus* Fairmaire, 1882a: 71

**Type species.***Brachyphrynusspissicornis* Fairmaire, 1882 (by monotypy)

*abyssinicusabyssinicus* (Haag-Rutenberg, 1871a: 39) *Phrynocolus* [Koch, 1951: 85]

**Type data.** Syntypes (Basel Museum, British Museum)

**Notes.** Treated as a synonym of *Psammophanescatenatus* (Reiche, 1850) by Koch (1953a). However, this interpretation was not adopted by the subsequent authors (Kaszab 1963).

*abyssinicusbreuningi* Kaszab, 1963: 348

**Type data.** Holotype (Tervuren Museum) and paratypes (Budapest Museum, Tervuren Museum)

*gallanus* (Wilke, 1921: 163) *Phrynocolus* [Koch, 1951: 85]

**Type data.** Holotype (Berlin Museum)

*kuntzeni* (Wikle, 1921: 163) *Phrynocolus* [Koch, 1951: 85]

**Type data.** Syntypes (Basel Museum)

*petrosuserlangeri* (Wilke, 1921: 164) *Phrynocolus* [Koch, 1951: 85]

**Type data.** Syntypes (Basel Museum)

*petrosuspetrosus* (Gerstaecker, 1871: 59) *Phrynocolus* [Koch, 1951: 85]

**Type data.** Holotype (Berlin Museum)

= *Phrynocolusikutanus* Fairmaire, 1897: 113 [syn. by Wilke (1921: 171)]

**Type data.** Syntypes (Paris Museum)

*placidus* (Kolbe, 1885: 112) *Phrynocolus* [Koch, 1951: 85]

**Type data.** Holotype (Geneva Museum)

*somalicus* (Wilke, 1921: 164) *Phrynocolus* [Koch, 1951: 85]

**Type data.** Syntypes (Basel Museum)

*spissicornis* Fairmaire, 1882a: 72

**Type data.** Holotype (Paris Museum)

*subnodosus* (Gebien, 1937b: 48) *Phrynocolus* [Koch, 1951: 85]

**Type data.** Holotype (Trieste Museum) and paratype (Basel Museum)

*wachei* (Wilke, 1921: 163) *Phrynocolus* [Koch, 1951: 85]

**Type data.** Syntypes (Basel Museum)

##### Genus *Dichtha* Haag-Rutenberg, 1871a: 39

**Type species.***Cryptogeniusinflatus* Gerstacker, 1854 (by original designation)

**Notes.** “*Dichtha incantatoris* / *incantatoria* Koch, 1952” is considered here as a nomen nudum, since no published record of this species-group name was found during the present work.

*cubica* (Guérin-Méneville, 1845: 285) *Moluris* [Haag-Rutenberg, 1871a: 41]

**Type data.** Holotype (Paris Museum)

*inflata* (Gerstaecker, 1854: 532) *Cryptogenius* [Haag-Rutenberg, 1871a: 41]

**Type data.** Syntypes (Berlin Museum)

*modesta* Robiche, 2013: 159

**Type data.** Holotype (Paris Museum) and paratypes (Paris Museum, Gérard Robiche collection)

*transvalica* Brancsik, 1914: 65

**Type data.** Syntypes (Budapest Museum)

*quedenfeldti* Kolbe, 1886: 293

**Type data.** Syntypes (Berlin Museum)

##### Genus *Distretus* Haag-Rutenberg, 1871a: 42

**Type species.***Molurisamplipennis* Fåhraeus, 1870 (by subsequent designation by Rye (1873: 287))

###### Subgenus Distretus Haag-Rutenberg, 1871a: 42

**Type species.***Molurisamplipennis* Fåhraeus, 1870 (by subsequent designation by Rye (1873: 287))

*amplipennis* (Fåhraeus, 1870: 262) *Moluris* [Haag-Rutenberg, 1871a: 43]

**Type data.** Syntypes (Naturhistoriska riksmuseet)

*dissociatus* (Péringuey, 1899: 274) *Psammodes* [Gebien, 1937a: 757]

**Type data.** Holotype (Cape Museum)

*fahraei* Haag-Rutenberg, 1871a: 43

**Type data.** Holotype (Naturhistoriska riksmuseet)

*inaequalis* Fairmaire, 1894: 320

**Type data.** Holotype (Basel Museum)

*mashunus* (Péringuey, 1896: 167) *Amiantus* [Gebien, 1937a: 757]

**Type data.** Holotype (Cape Museum)

*undosus* Kolbe, 1886: 291

**Type data.** Syntypes (Berlin Museum)

*undatus* (Haag-Rutenberg, 1875: 69) *Amiantus* [Gebien, 1910b: 153]

**Type data.** Syntypes (British Museum, Munich Museum – Haag-Rutenberg coll.)

*variabilis* Gebien, 1910a: 153

**Type data.** Syntypes (Basel Museum, Tervuren Musuem)

*variolosus* (Guérin-Méneville, 1854: 245) *Moluris* [Haag-Rutenberg, 1871a: 44]

**Type data.** Holotype (Warsaw Museum – Dohrn coll.)

= *Molurispilicornis* Fåhraeus, 1870: 263 [syn. by Fairmaire (1894: 320)]

**Type data.** Syntypes (Naturhistoriska riksmuseet)

*vietus* (Péringuey, 1899: 273) *Psammodes* [Gebien, 1937a: 757]

**Type data.** Holotype (Cape Museum)

###### Subgenus Perdistretus Koch, 1953b: 65

**Type species.**Distretus (Perdistretus) vilhenai Koch, 1953 (by original designation)

*acutecostatus* (Fairmaire, 1888b: 260) *Dichtha* [Koch, 1953: 65]

**Type data.** Syntypes (Basel Museum, Leiden Museum)

*angolanus* Koch, 1953b: 72

**Type data.** Holotype (Cape Museum)

*angustipennis* Péringuey, 1892: 52

**Type data.** Holotype (Cape Museum)

**Notes.** Considered as a synonym of *Perdistretusacutecostatus* Fairmaire, 1888b by Gebien (1937a). However, this interpretation was not adopted by the subsequent authors (e.g., Koch 1953b: 68).

Originally described in combination with the generic name “Dichtrethus”, which is treated as an incorrect subsequent spelling of *Distretus*, not in prevailing usage.

*auritus* Koch, 1953b: 73

**Type data.** Holotype (Munich Museum)

*duartei* Koch, 1953b: 70

**Type data.** Holotype (Munich Museum) and paratypes (Basel Museum, Ditsong Museum, Munich Museum)

*gracilis* Gebien, 1910a: 152

**Type data.** Syntypes (Tervuren Museum)

*mormolyce* Koch, 1953b: 68

**Type data.** Holotype (Munich Museum) and paratype (Basel Museum, Ditsong Museum, Munich Museum)

*seminitidus* Quedenfeldt, 1888: 184

**Type data.** Holotype (Berlin Museum)

*strioliceps* Koch, 1953b: 71

**Type data.** Holotype (Munich Museum)

*schoutedeni* Koch, 1954a: 435

**Type data.** Holotype (Tervuren Museum) and paratypes (Ditsong Museum, Tervuren Museum)

*upembensis* Koch, 1954a: 437

**Type data.** Holotype (National Congo) and paratypes (Ditsong Museum, National Congo)

*vilhenai* Koch, 1953b: 65

**Type data.** Holotype (Dundo Museum) and paratype (Ditsong Museum, Dundo Museum)

##### Genus *Euphrynus* Fairmaire, 1897: 114

**Type species.***Euphrynusspinithorax* Fairmaire, 1897 (by monotypy)

*carinatus* (Fåhraeus, 1870: 281) *Amiantus* [Koch, 1952: 345]

**Type data.** Holotype (Naturhistoriska riksmuseet)

= *Amiantuscostatus* Péringuey, 1896: 168 [syn. by Gebien (1937a: 758)]

**Type data.** Syntypes (Cape Museum)

*jansei* Koch, 1952: 343

**Type data.** Holotype (Ditsong Museum) and paratypes (Basel Museum, Ditsong Museum)

*sexdentatus* Koch, 1952: 344

**Type data.** Holotype (Ditsong Museum) and paratype (Basel Museum)

*spinithorax* Fairmaire, 1897: 114

**Type data.** Holotype (Geneva Museum)

##### Genus *Glyptophrynus* Fairmaire, 1899b: 532

**Type species.***Glyptophrynustenuesculptus* Fairmaire, 1899 (by monotypy)

**Notes.** Treated as a synonym of *Phrynocolus* by several authors (e.g., Gebien 1910b). However, this interpretation was not adopted in the more recent taxonomic works (i.e., Koch 1962a).

*cordipennis* Koch, 1962a: 12

**Type data.** Holotype and paratypes (Ditsong Museum)

*madecassusmadecassus* (Fairmaire, 1901: 183) *Phrynocolus* [Wilke, 1921: 174]

**Type data.** Syntypes (Berlin Museum, British Museum)

*madecassuspauliani* Koch, 1962a: 15

**Type data.** Holotype (Ditsong Museum) and paratypes (Basel Museum, Budapest Museum, Ditsong Museum)

*ovipennisovipennis* (Fairmaire, 1899b: 533) *Phrynocolus* [Wilke, 1921: 174]

**Type data.** Syntypes (Paris Museum)

*ovipennisserricostatus* Koch, 1962a: 17

**Type data.** Holotype (Ditsong Museum)

*tenuesculptuscrassigranulatus* Wilke, 1921: 174

**Type data.** Syntypes (Berlin Museum)

*tenuesculptustenuesculptus* Fairmaire, 1899b: 532

**Type data.** Syntypes (Basel Museum, Paris Museum)

*voeltzkowi* Wilke, 1921: 174

**Type data.** Syntypes (Berlin Museum)

##### Genus *Melanolophus* Fairmaire, 1882a: 69

**Type species.***Melanolophusseptemcostatus* Fairmaire, 1882 (by monotypy)

**Notes.** Treated as a synonym of *Amiantus* by several authors (e.g., Fairmaire 1887, Gebien 1910b, 1937a). However, this interpretation was not adopted in the more recent taxonomic works (e.g., Koch 1956, 1960).

*gridellii* Koch, 1956: 170

**Type data.** Holotype (Trieste Museum) and paratypes (Ditsong Museum, Trieste Museum)

*lomianus* Koch, 1956: 173

**Type data.** Holotype (Trieste Museum) and paratypes (Basel Museum, Ditsong Museum, Trieste Museum)

*pictetipicteti* (Haag-Rutenberg, 1871a: 46) *Amiantus* [Koch, 1960: 258]

**Type data.** Holotype (Geneva Museum)

*pictetiseptemcostatus* Fairmaire, 1882a: 70

**Type data.** Holotype (Basel Museum)

**Notes.** Synonymised with the nominotypical form by Gestro (1883). However, this decision was not adopted by the subsequent authors (Koch 1960).

*pictetisplendidus*Koch 1960: 257

**Type data.** Holotype (Ditsong Museum)

*praeplanatus* Koch, 1960: 261

**Type data.** Holotype (Ditsong Museum)

*sexcostatusbenardellii* Koch, 1960: 258

**Type data.** Holotype (Ditsong Museum) and paratypes (Geneva Museum, Munich Museum)

*sexcostatusgibbithorax* Koch, 1956: 175

**Type data.** Holotype (Trieste Museum) and paratypes (Ditsong Museum, Trieste Museum)

*sexcostatushellardi* Koch, 1960: 258

**Type data.** Holotype and paratype (Ditsong Museum)

*sexcostatussexcostatus* (Gahan, 1900: 28) *Amiantus* [Koch, 1956: 175]

**Type data.** Holotype (British Museum)

*sexcostatustuberculatus* Koch, 1960: 258

**Type data.** Holotype (Ditsong Museum) and paratypes (Berlin Museum, Munich Museum, Milan Museum)

##### Genus *Moluris* Latreille, 1802: 169

**Type species.***Tenebriogibbus* Pallas, 1781 (by monotypy)

= *Physodera* Solier, 1843: 78 [junior subjective synonym proposed by Lacordaire (1859); junior homonym of *Physodera* Eschscholtz, 1829 (Coleoptera: Carabidae)]

**Type species.***Pimeliagibba* Fabricius, 1787 (by original designation)

*chevrolati* Haag-Rutenberg, 1871a: 52

**Type data.** Holotype (Paris Museum)

*discoidea* Guérin-Méneville, 1845: 286

**Type data.** Holotype (Paris Museum)

**Notes.** According to Haag-Rutenberg (1871a), this species may be a member of *Distretus*.

*ferrari* Haag-Rutenberg, 1871a: 55

**Type data.** Holotype (Vienna Museum)

*gibba* (Pallas, 1781: 46) *Tenebrio* [Latreille, 1802: 169]

**Type data.** Syntypes (Humboldt University – Pallas collection)

= *Pimeliaplanata* Thunberg, 1787: 49 [syn. by Haag-Rutenberg (1871a: 53)]

**Type data.** Syntypes (Uppsala University)

= *Pimeliagibba* Fabricius, 1787: 24 [junior secondary homonym of *Tenebriogibba* Pallas, 1781: 46]

**Type data.** Syntypes (Copenhagen Museum, Glasgow Museum, Kiel Museum)

= *Pimeliabistriata* Herbst, 1799: 50 [syn. by Haag-Rutenberg (1871a: 53)]

**Type data.** Syntypes (Berlin Museum)

*gibbicollis* Haag-Rutenberg, 1871a: 107

**Type data.** Holotype (Munich Museum – Haag-Rutenberg coll.)

*gibbosa* (Thunberg, 1787: 49) *Pimelia* [Solier, 1843: 79]

**Type data.** Syntypes (Uppsala University)

= *Opatrumgibbosum* Thunberg, 1821: 33 [syn. by Ferrer (2009: 116)]

**Type data.** Holotype (Uppsala University)

*gibbosaglobulicollis* Solier, 1843: 80

**Type data.** Holotype (Torino Museum – Spinola coll.)

*herbsti* Haag-Rutenberg, 1871a: 54, replacement name

= *Pimeliagibba* Herbst, 1799: 48 [junior secondary homonym of *Tenebriogibba* Pallas, 1781: 46]

**Type data.** Holotype (Berlin Museum)

*nitida* Haag-Rutenberg, 1871a: 52

**Type data.** Syntypes (Brussels Museum)

*pseudonitida* Péringuey, 1908: 406

**Type data.** Holotype (Cape Museum)

*redtenbacheri* Haag-Rutenberg, 1871a: 56

**Type data.** Holotype (Vienna Museum)

*rustica* Haag-Rutenberg, 1871a: 54

**Type data.** Holotype (Naturhistoriska riksmuseet)

*semiscabra* Solier, 1843: 81

**Type data.** Holotype (Torino Museum – Spinola coll.)

*strigosa* (Herbst, 1799: 49) *Pimelia* [Haag-Rutenberg, 1871a: 55]

**Type data.** Syntypes (Berlin Museum)

= *Molurisrouleti* Solier, 1843: 80 [syn. by Haag-Rutenberg (1871a: 55)]

**Type data.** Holotype (Geneva Museum – Gory collection)

*tuberculata* Haag-Rutenberg, 1871a: 107

**Type data.** Syntypes (Paris Museum)

##### Genus *Phrynocolus* Lacordaire, 1859: 201

**Type species.***Cryptogeniusdentatus* Solier, 1843 (by original designation)

###### Subgenus Phrynocolopsis Koch, 1951: 93

**Type species.***Phrynocolusfrondosus* Gerstacker, 1871 (original designation)

*denhardtidenhardti* Wilke, 1921: 165 [Koch, 1951: 93]

**Type data.** Holotype (Berlin Museum)

*denhardtifractus* Koch, 1969: 13

**Type data.** Holotype (Munich Museum)

*denhardtihumeralis* Koch, 1969: 12

**Type data.** Holotype (Geneva Museum)

*desaegeri* Koch, 1969: 15

**Type data.** Holotype (Brussels Museum) and paratypes (Brussels Museum, Tervuren Museum)

*frondosus* Gerstaecker, 1871: 59 [Koch, 1951: 93]

**Type data.** Holotype (Cape Museum)

*transversus* Fairmaire, 1887: 183 [Koch, 1951: 93]

**Type data.** Holotype (Paris Museum)

*subfrondosus* Wilke, 1921: 166 [Koch, 1951: 93]

**Type data.** Syntypes (Basel Museum, Berlin Museum)

###### Subgenus Phrynocolus Lacordaire, 1859: 201, replacement name

= *Cryptogenius* Solier, 1843: 37 [junior homonym of *Cryptogenius* Westwood, 1842 (Coleoptera: Hybosoridae)]

**Type species.***Cryptogeniusdentatus* Solier, 1843 (by original designation)

*dentatus* (Solier, 1843: 38) *Cryptogenius* [Lacordaire, 1859: 201]

**Type data.** Syntypes (Cape Museum, Paris Museum)

*felinus* Koch, 1951: 89

**Type data.** Holotype (Paris Museum) and paratypes (Basel Museum)

*spinolaispinolai* (Solier, 1843: 39) *Cryptogenius* [Lacordaire, 1859: 201]

**Type data.** Holotype (Warsaw Museum – Dupont collection)

**Notes.**Koch (1951: 88) described “var. emarginatus”. He expressly gave it infrasubspecific rank since he also designated taxa at the subspecies level. Therefore, according to the art. 45.6.4. of the ICZN (1999) is should not be treated as an available subspecies.

= *Phrynocolusniloticus* Haag-Rutenberg, 1871a: 38 [syn. by Koch (1951: 88)]

**Type data.** Syntypes (Basel Museum, British Museum)

= *Phrynocoluscultratus* Fairmaire, 1891a: 249 [syn. by Wilke (1921: 171)]

**Type data.** Holotype (Paris Museum)

*spinolaiwilkei* Koch, 1951: 88

**Type data.** Holotype and paratypes (Basel Museum)

*theryi* Koch, 1951: 89

**Type data.** Holotype (Basel Museum) and paratypes (Basel Museum, Budapest Museum, Ditsong Museum)

###### Subgenus Spinophrynus Koch, 1951: 90

**Type species.***Phrynocolusspinipennis* Gebien, 1910 (by original designation)

*spinipennis* Gebien, 1910a: 154

**Type data.** Syntypes (Basel Museum)

incertae sedis

*menghallensis* Wilke, 1922: 381

**Type data.** Holotype (Berlin Museum)

**Notes.** This species and Wilke’s (1922) publication were overlooked by Koch (1951) and therefore the correct placement of this species in one of the valid subgenera is uncertain.

##### Genus *Phrynophanes* Koch, 1951: 92

**Type species.***Molurisgredleri* Haag-Rutenberg, 1877 (by original designation)

**Notes.** Originally described as a subgenus of *Phrynocolus*. Elevated to generic level by Koch (1960).

*citernii* (Gridelli, 1939b: 229) *Psammodes* [Koch, 1969: 21]

**Type data.** Holotype (Geneva Museum)

*cryptisculptus* Koch, 1969: 4

**Type data.** Holotype (Munich Museum)

*discoideus* (Fairmaire, 1891b: CCXCIV) *Phrynocolus* [Koch, 1969: 17]

**Type data.** Holotype (Paris Museum)

*gredleri* (Haag-Rutenberg, 1877: 515) *Moluris* [Koch, 1951: 93]

**Type data.** Holotype (Munich Museum – Haag-Rutenberg coll.)

= *Phrynocolusunicarinatus* Wilke, 1921: 170 [syn. by Koch (1969: 18)]

**Type data.** Holotype (Basel Museum)

*humilis* (Wilke, 1921: 170) *Phrynocolus* [Koch, 1951: 93]

**Type data.** Holotype (Berlin Museum)

*lateritius* (Wilke, 1921: 169) *Phrynocolus* [Koch, 1951: 93]

**Type data.** Syntypes (Basel Museum, Berlin Museum)

*neumanni* (Wilke, 1921: 169) *Phrynocolus* [Koch, 1951: 93]

**Type data.** Holotype (Basel Museum)

*reticulatus* Wilke, 1921: 169

**Type data.** Syntypes (Basel Museum, Berlin Museum)

*schereri* Koch, 1969: 20

**Type data.** Holotype (Munich Museum)

*scortecii* Koch, 1969: 19

**Type data.** Holotype (Geneva Museum)

*schoutedeni* (Koch, 1951: 92) *Phrynocolus* [Koch, 1969: 24]

**Type data.** Holotype (Brussels Museum) and paratypes (Basel Museum, Brussels Museum)

*squamifergridellianus* Koch, 1960: 262

**Type data.** Holotype and paratype (Ditsong Museum)

*squamifersquamifer* (Gridelli, 1939b: 228) *Psammodes* [Koch, 1960: 262]

**Type data.** Syntypes (Geneva Museum, Trieste Museum)

##### Genus *Physophrynus* Fairmaire, 1882b: L

**Type species.***Physophrynusburdoi* Fairmaire, 1882 (by monotypy)

*bufo* (Haag-Rutenberg, 1871a: 48) *Amiantus* [Koch, 1953a: 177]

**Type data.** Holotype (Warsaw Museum – Dohrn coll.)

= *Amiantusreichardi* Kolbe, 1886: 228 [syn. by Koch (1953a: 177)]

**Type data.** Holotype (Berlin Museum)

*burdoi* Fairmaire, 1882b: L

**Type data.** Holotype (Paris Museum)

*bredoi* Mal, 2005: 9

**Type data.** Holotype and paratypes (Brussels Museum)

*bufocrenatocostatus* (Fairmaire, 1887: 181) *Amiantus* [Koch, 1953a: 177]

**Type data.** Holotype (Fairmaire collection)

*haroldi* (Haag-Rutenberg, 1871a: 47) *Amiantus* [Koch, 1953a: 177]

**Type data.** Syntypes (British Museum, Munich Museum – Haag-Rutenberg coll.)

*kaszabi* Koch, 1953a: 176

**Type data.** Holotype (Budapest Museum)

*manicanus* (Péringuey, 1899: 226) *Amiantus* [Koch, 1953a: 177]

**Type data.** Holotype (Cape Museum)

*revoili* Fairmaire, 1887: 182

**Type data.** Holotype (Paris Museum)

##### Genus *Psammodes* Kirby, 1819: 412

**Type species.***Psammodeslongicornis* Kirby, 1819 (by monotypy)

= *Piesomera* Solier, 1843: 77 [junior subjective synonym proposed by Gebien (1937a: 759)]

**Type species.***Pimeliascabra* Fabricius, 1775 (by monotypy)

= *Psammodophysis* Péringuey, 1899: 296 [junior subjective synonym proposed by Gebien (1910b: 154)]

**Type species.***Psammodophysisprobes* Péringuey, 1899 (**here designated**)

= *Parmularia* Koch, 1955: 35, syn. n. [homonym of *Parmularia* Macgillivray, 1887 (Bryozoa: Cheilostomida)]

**Type species.***Psammodescaffra* Fåhraeus, 1870 (by monotypy)

**Notes.** Originally described as a monotypic subgenus of *Psammodes*. Interpreted here as a synonym of the nominal form, as sustaining a weakly defined and monotypic subgenus within present *Psammodes* seems to be unjustified.

*algoensis* Péringuey, 1899: 275

**Type data.** Holotype (Cape Museum)

*asperulipennis* Fairmaire, 1888: 193

**Type data.** Holotype (Paris Museum)

*atratus* Haag-Rutenberg, 1871a: 73, in key

**Type data.** Syntypes (British Museum, Munich Museum – Haag-Rutenberg coll.)

**Notes.** A detailed morphological description was provided by Haag-Rutenberg (1871b: 60).

*basuto* Koch, 1953c: 7

**Type data.** Holotype (Ditsong Museum) and paratypes (Ditsong Museum, Lund University, Munich Museum)

*barbatus* Fåhraeus, 1870: 268

**Type data.** Holotype (Naturhistoriska riksmuseet)

= *Psammodespraeliator* Péringuey, 1899: 272 [syn. by Gebien (1937a: 765)]

**Type data.** Holotype (Cape Museum)

*batesi* Haag-Rutenberg, 1871a: 77

**Type data.** Holotype (Munich Museum – Haag-Rutenberg coll.)

**Notes.** Treated as a synonym of *Psammodesponderosus* Fåhraeus, 1870 by Péringuey (1904). However, this interpretation was not adopted by subsequent authors (Gebien 1937, Koch 1953c).

*bennigseni* Kraatz, 1897: 46

**Type data.** Holotype (Berlin Museum)

*blapsoides* Haag-Rutenberg, 1871a: 63, in key

**Type data.** Holotype (Munich Museum – Haag-Rutenberg coll.)

**Notes.** A detailed morphological description was provided by Haag-Rutenberg (1871b: 43).

*brunneusbrunneus* (Olivier, 1795: 14) *Pimelia* [Haag-Rutenberg, 1871b: 42]

**Type data.** Syntypes (Paris Museum)

*brunneusrufocastaneus* Haag-Rutenberg, 1871b: 42

**Type data.** Holotype (Munich Museum – Haag-Rutenberg coll.)

*brunnipes* Haag-Rutenberg, 1871a: 72, in key

**Type data.** Holotype (Munich Museum – Haag-Rutenberg coll.)

**Notes.** A detailed morphological description was provided by Haag-Rutenberg (1871b: 54).

*caelatus* Péringuey, 1899: 281

**Type data.** Syntypes (Cape Museum)

*caffra* Fåhraeus, 1870: 265

**Type data.** Holotype (Naturhistoriska riksmuseet)

*caraboides* Haag-Rutenberg, 1871a: 69, in key

**Type data.** Syntypes (British Museum, Cape Museum, Geneva Museum)

**Notes.** A detailed morphological description was provided by Haag-Rutenberg (1871b: 50).

*carinatus* Haag-Rutenberg, 1871a: 103

**Type data.** Syntypes (Berlin Museum)

*clarus* Haag-Rutenberg, 1873: 76

**Type data.** Holotype (Munich Museum – Haag-Rutenberg coll.)

*collaris* Haag-Rutenberg, 1871b: 101

**Type data.** Holotype (Berlin Museum)

*coloratus* Haag-Rutenberg, 1871a: 71, in key

**Type data.** Holotype (Berlin Museum)

**Notes.** A detailed morphological description was provided by Haag-Rutenberg (1871b: 57).

*comatus* Haag-Rutenberg, 1871a: 106

**Type data.** Holotype (Munich Museum – Haag-Rutenberg coll.)

*comptus* Haag-Rutenberg, 1871a: 109

**Type data.** Holotype (Munich Museum – Haag-Rutenberg coll.)

*convexus* (Solier, 1843: 91) *Phanerotoma* [Haag-Rutenberg, 1871b: 61]

**Type data.** Holotype (Paris Museum)

*coriaceus* (Gerstaecker, 1854: 532) *Phanerotoma* [Haag-Rutenberg, 1871b: 68]

**Type data.** Syntypes (Berlin Museum)

= *Psammodesmanifestus*Péringuey 1899: 274 [syn. by Gebien (1937a: 768)]

**Type data.** Holotype (Cape Museum)

*costalis* Haag-Rutenberg, 1871a: 97

**Type data.** Syntypes (Munich Museum – Haag-Rutenberg coll., Royal Museum)

*dejeani* (Solier, 1843: 71) *Moluris* [Haag-Rutenberg, 1871a: 92]

**Type data.** Holotype (Paris Museum)

*depressicollis* Haag-Rutenberg, 1871a: 72, in key

**Type data.** Syntypes (British Museum)

**Notes.** A detailed morphological description was provided by Haag-Rutenberg (1871b: 67).

*devexus* Fåhraeus, 1870: 266

**Type data.** Holotype (Cape Museum)

*diabolicusdiabolicus* Koch, 1952: 335

**Type data.** Holotype (Ditsong Museum) and paratype (Basel Museum, Budapest Museum, Ditsong Museum)

*diabolicustactilis* Koch, 1962b: 123

**Type data.** Holotype and paratype (Ditsong Museum)

*difficilis* Haag-Rutenberg, 1871a: 73, in key

**Type data.** Syntypes (British Museum, Geneva Museum)

**Notes.** A detailed morphological description was provided by Haag-Rutenberg (1871b: 61).

*dilutus* Haag-Rutenberg, 1871a: 64, in key

**Type data.** Holotype (Warsaw Museum – Dohrn coll.)

**Notes.** A detailed morphological description was provided by Haag-Rutenberg (1871b: 80).

*dimidiatus* Haag-Rutenberg, 1871a: 71, in key

**Type data.** Syntypes (British Museum, Munich Museum – Haag-Rutenberg coll.)

**Notes.** A detailed morphological description was provided by Haag-Rutenberg (1871b: 88).

*discrepans* Péringuey, 1904: 230

**Type data.** Holotype (Cape Museum)

*dohrni* Haag-Rutenberg, 1871a: 67, in key

**Type data.** Holotype (Munich Museum – Haag-Rutenberg coll.)

**Notes.** A detailed morphological description was provided by Haag-Rutenberg (1871b: 36).

*eberlanzi* Koch, 1952: 337

**Type data.** Holotype (Ditsong Museum)and paratypes (British Museum, Budapest Museum, Ditsong Museum)

*egregius* Haag-Rutenberg, 1871a: 74

**Type data.** Syntypes (Munich Museum – Haag-Rutenberg coll.)

*ethologus* Koch, 1953c: 10

**Type data.** Holotype (Ditsong Museum) and paratype (Durban Museum)

*expletus* Quedenfeldt, 1885: 4

**Type data.** Syntypes (Berlin Museum)

**Notes.** Type specimens of this species were unknown to Koch (1952); however, base on the original description he indicated this species to be a potential member of *Ocnodes*.

*fartus* Péringuey, 1904: 232

**Type data.** Holotype (Cape Museum)

= *Psammodesillotus* Péringuey, 1904: 233 [syn. by Gebien 1937a: 764]

**Type data.** Syntypes (Cape Museum)

*Psammodesferrugineus* Haag-Rutenberg, 1871a: 79

**Type data.** Syntypes (Cape Museum, Munich Museum – Haag-Rutenberg coll.)

*flagrans* Péringuey, 1899: 273

**Type data.** Holotype (Cape Museum)

*fragilis* Haag-Rutenberg, 1871a: 68, in key

**Type data.** Holotype (Munich Museum – Haag-Rutenberg coll.)

**Notes.** A detailed morphological description was provided by Haag-Rutenberg (1871b: 32).

*fritschi* Haag-Rutenberg, 1871a: 103

**Type data.** Holotype (Munich Museum – Haag-Rutenberg coll.)

*funestus* Haag-Rutenberg, 1871a: 72, in key

**Type data.** Holotype (Munich Museum – Haag-Rutenberg coll.)

**Notes.** A detailed morphological description was provided by Haag-Rutenberg (1871b: 79).

*gariesus* Péringuey, 1899: 282

**Type data.** Holotype (Cape Museum)

*gerstaeckeri* Haag-Rutenberg, 1871b: 100

**Type data.** Holotype (Berlin Museum)

*gibbuscoelata* (Solier, 1843: 64) *Moluris* [Haag-Rutenberg, 1871a: 82]

**Type data.** Holotype (Paris Museum)

*gibbusgibbus* (Linnaeus, 1760: 226) *Tenebrio* [Ferrer & Holson, 2009: 30]

**Type data.** Lectotype, designated by Ferrer and Holson (2009) (Naturhistoriska riksmuseet)

= *Pimeliastriata* Fabricius, 1775: 251 [syn. by Ferrer and Holson (2009: 34)]

**Type data.** Syntypes (Copenhagen Museum, Kiel Museum)

= *Tenebrioglandiformis* Pallas, 1781: 45 [syn. by Haag-Rutenberg, 1871a: 82]

**Type data.** Syntypes (Humboldt University – Pallas collection)

*gibbusgravidus* (Solier, 1843: 69) *Moluris* [Haag-Rutenberg, 1871a: 85]

**Type data.** Syntypes (Paris Museum)

*gibbushemisphaericus* (Solier, 1843: 68) *Moluris* [Haag-Rutenberg, 1871a: 85]

**Type data.** Holotype (Paris Museum)

*gibbusnigrocostatus* Haag-Rutenberg, 1871a: 85

**Type data.** Syntypes (Munich Museum)

*gibbussolieri* Gebien, 1910b: 161, replacement name

= *Molurisunicolor* Solier, 1843: 64 [junior secondary homonym of *Pimeliaunicolor* Fabricius, 1787: 316].

**Type data.** (Warsaw Museum – Dupont collection)

*gibbusunicolor* (Fabricius, 1787: 316) *Pimelia* [Haag-Rutenberg, 1871a: 82]

**Type data.** Syntypes (British Museum, Kiel Museum, Naturhistoriska riksmuseet)

*glaber* Koch, 1953c: 10

**Type data.** Holotype (Lund University) and paratypes (Ditsong Musuem, Lund University)

*glabratusbienus* Koch, 1953b: 77

**Type data.** Holotype (Munich Museum)

*glabratusglabratus* Harold, 1878: 106

**Type data.** Holotype (Berlin Museum)

*grandis* (Solier, 1843: 90) *Phanerotoma* [Gemminger, 1870: 122]

**Type data.** Holotype (Paris Museum)

= *Psammodeslugubris* Fåhraeus, 1870: 269 [syn. by Haag-Rutenberg (1871b: 58)]

**Type data.** Holotype (Naturhistoriska riksmuseet)

*granulatus* (Solier, 1843: 87) *Phanerotomea* [Haag-Rutenberg, 1871b: 53]

**Type data.** Holotype (Paris Museum)

*granulifer* Haag-Rutenberg, 1871b: 54

**Type data.** Syntypes (Geneva Museum)

*guillarmodi* Koch, 1952: 340

**Type data.** Holotype (Ditsong Museum)

*haagi* Gebien, 1910b: 156, replacement name

= *Psammodesobliteratus* Haag-Rutenberg, 1871a: 103 [junior secondary homonym of *Hypomelusobliteratus* Solier, 1843: 97]

**Type data.** Holotype (Munich Museum)

*hirtipennis* Haag-Rutenberg, 1871a: 105

**Type data.** Holotype (Munich Museum – Haag-Rutenberg coll.)

*hirtipes* (Laporte, 1840: 198) *Moluris* [Gebien, 1910b: 159]

**Type data.** Holotype (Paris Museum)

= *Molurisreichei* Solier, 1843: 67 [syn. by Haag-Rutenberg (1871a: 78)]

**Type data.** Holotype (Paris Museum)

*hirtus* (Bertoloni, 1849: 399) *Moluris* [Gerstaecker, 1854: 532]

**Type data.** Holotype (Bologna Museum)

*herculeanus* Haag-Rutenberg, 1871a: 68, in key

**Type data.** Syntypes (Naturhistoriska riksmuseet)

**Notes.** A detailed morphological description was provided by Haag-Rutenberg (1871b: 86).

*herero* Péringuey, 1908: 409

**Type data.** Holotype (Cape Museum)

*hottentottus* Péringuey, 1899: 267

**Type data.** Holotype (Cape Museum)

*incongruens* Péringuey, 1899: 281

**Type data.** Syntypes (Cape Museum)

*infernalis* Harold, 1878: 106

**Type data.** Syntypes (Munich Museum)

*intermedius* Péringuey, 1899: 272

**Type data.** Holotype (Cape Museum)

*janitor* Koch, 1953c: 11

**Type data.** Holotype (Ditsong Museum) and paratypes (Cape Museum, Ditsong Museum, Lund University, Rhodes University)

*kamagasus* Péringuey, 1908: 409

**Type data.** Holotype (Cape Museum)

*kirschi* Haag-Rutenberg, 1871b: 102

**Type data.** Holotype (Munich Museum – Haag-Rutenberg coll.)

*kubub* Péringuey, 1908: 408

**Type data.** Syntypes (Cape Museum)

*kuisip* Péringuey, 1908: 504

**Type data.** Holotype (Cape Museum)

**Notes.** Originally described under the name *Psammodestuberculifer* (intended redescription; page: 407). However, in erratum (page: 504), renamed *kuisip*.

*lanuginosus* Haag-Rutenberg, 1871a: 105

**Type data.** Holotype (Warsaw Museum – Dohrn coll.)

*lethargicus* Péringuey, 1899: 284

**Type data.** Holotype (Cape Museum)

*laevicollis* (Solier, 1843: 65) *Moluris* [Haag-Rutenberg, 1871a: 78]

**Type data.** Holotype (Paris Museum)

*longicornis* Kirby, 1819: 480

**Type data.** Syntypes (British Museum)

= *Phanerotomaruficore* Solier, 1843: 86 [syn. by Haag-Rutenberg (1871b: 45)]

**Type data.** Syntypes (Paris Museum)

*longipes* Haag-Rutenberg, 1871a: 108

**Type data.** Holotype (Munich Museum – Haag-Rutenberg coll.)

*lucidus* Fåhraeus, 1870: 267

**Type data.** Holotype (Naturhistoriska riksmuseet)

*mashunus* Péringuey, 1899: 269

**Type data.** Syntypes (Cape Museum)

*memnonius* Haag-Rutenberg, 1871a: 50, in key

**Type data.** Holotype (British Museum)

**Notes.** A detailed morphological description was provided by Haag-Rutenberg (1871b: 50).

*mimipinguis* Koch, 1953c: 9

**Type data.** Holotype (Lund University) and paratypes (Ditsong Museum, Munich Museum)

*moschleri* Haag-Rutenberg, 1875: 73

**Type data.** Holotype (Munich Museum – Haag-Rutenberg coll.)

*muata* Harold, 1878: 106

**Type data.** Syntypes (Munich Museum, Warsaw Museum)

*mulleri* Péringuey, 1899: 269

**Type data.** Syntypes (Cape Museum)

*nigrisaxicola* Koch, 1953b: 78

**Type data.** Holotype (British Museum)

*nitens* Fåhraeus, 1870: 267

**Type data.** Holotype (Naturhistoriska riksmuseet)

*nitidicollis* Haag-Rutenberg, 1871a: 91

**Type data.** Holotype (Munich Museum – Haag-Rutenberg coll.)

*nitidipennis* (Fairmaire, 1897: 114) *Amiantus* [Gebien, 1937a: 763]

**Type data.** Holotype (Paris Museum)

*nitidissimus* Haag-Rutenberg, 1871a: 92

**Type data.** Holotype (Warsaw Museum – Dohrn coll.)

*obsulcatus* Haag-Rutenberg, 1871a: 72, in key

**Type data.** Holotype (Geneva Museum)

**Notes.** A detailed morphological description was provided by Haag-Rutenberg (1871b: 55).

*ovatus* (Solier, 1843: 90) *Phanerotoma* [Haag-Rutenberg, 1871b: 62]

**Type data.** Holotype (Paris Museum)

*ovipennis* Haag-Rutenberg, 1871a: 102

**Type data.** Holotype (Warsaw Museum – Dohrn coll.)

*perfidus* Péringuey, 1899: 283

**Type data.** Holotype (Cape Museum)

*piceus* Haag-Rutenberg, 1871a: 67, in key

**Type data.** Syntype (Geneva Museum)

**Notes.** A detailed morphological description was provided by Haag-Rutenberg (1871b: 33).

*pilifer* Haag-Rutenberg, 1871a: 69, in key

**Type data.** Holotype (British Museum – Bates coll.)

**Notes.** A detailed morphological description was provided by Haag-Rutenberg (1871b: 37).

*pilosellus* Haag-Rutenberg, 1875: 71

**Type data.** Syntypes (British Museum – Bates coll.)

*pilosipennis* Haag-Rutenberg, 1871a: 89

**Type data.** Holotype (Munich Museum – Haag-Rutenberg coll.)

*pilosus* (Thunberg, 1787: 49) *Pimelia* [Haag-Rutenberg, 1871a: 104]

**Type data.** Syntypes (Uppsala University)

*pinguis* (Solier, 1843: 70) *Moluris* [Haag-Rutenberg, 1871a: 86]

**Type data.** Holotype (Paris Museum)

= *Psammodesrotundipennis* Péringuey, 1899: 268 [syn. by Gebien (1937a: 765)]

**Type data.** Holotype (Cape Museum)

*placidus* Péringuey, 1899: 280

**Type data.** Syntypes (Cape Museum)

*plicatus* (Solier, 1844: 72) *Moluris* [Haag-Rutenberg, 1871a: 95]

**Type data.** Holotype (Marseille Museum)

*plicipennis* Gemminger, 1870: 1899, replacement name

= *Phanerotomaplicatus* Solier, 1844: 299 [homonym of *Molurisplicatus* Solier, 1844: 284 published on the same date; Gemminger (1870) acted as First Reviser when he proposed the replacement name *Psammodesplicipennis* for the species *Phanerotomaplicatus* Solier, 1844: 87]

**Type data.** Holotype (Paris Museum)

*ponderosus* Fåhraeus, 1870: 264

**Type data.** Syntypes (Cape Museum, Naturhistoriska riksmuseet)

*probes* (Péringuey, 1899: 296) *Psammodophysis* [Gebien, 1910b: 159]

**Type data.** Holotype (Cape Museum)

*procerus* (Fåhraeus, 1870: 271) *Hypomelus* [Gebien, 1910b: 159]

**Type data.** Holotype (Naturhistoriska riksmuseet)

*procustes* (Westwood, 1875: 224) *Moluris* [Gebien, 1910b: 159]

**Type data.** Holotype (Oxford University – Westwood coll.)

= *Psammodesgiganteus* Haag-Rutenberg, 1879: 290 [syn. by Gebien (1937a: 768)]

**Type data.** Syntypes (British Museum, Munich Museum – Haag-Rutenberg coll.)

*productus* Haag-Rutenberg, 1871b: 101

**Type data.** Holotype (Berlin Museum)

*profanus* Péringuey, 1899: 271

**Type data.** Holotype (Cape Museum)

*propinquus* Quedenfeldt, 1885: 5

**Type data.** Syntypes (Berlin Museum)

*protensus* Haag-Rutenberg, 1871a: 73, in key

**Type data.** Syntypes (Munich Museum – Haag-Rutenberg coll.)

**Notes.** A detailed morphological description was provided by Haag-Rutenberg (1871b: 62).

*pubescens* (Solier, 1843: 85) *Phanerotomea* [Haag-Rutenberg, 1871b: 37]

**Type data.** Holotype (Paris Museum)

*pustulifer* Haag-Rutenberg, 1871a: 71, in key

**Type data.** Syntypes (Naturhistoriska riksmuseet)

**Notes.** A detailed morphological description was provided by Haag-Rutenberg (1871b: 52).

*quadricostatus* (Fåhraeus, 1870: 272) *Hypomelus* [Gebien, 1910b: 159]

**Type data.** Syntypes (Naturhistoriska riksmuseet)

*raucus* Haag-Rutenberg, 1875: 159

**Type data.** Syntypes (Munich Museum – Haag-Rutenberg coll.)

*refleximargo* (Gebien, 1920: 90) *Trachynotidus* [Gebien, 1937a: 771]

**Type data.** Holotype (Hamburg University – Michaelsen coll.)

*retrospinosus* Haag-Rutenberg, 1871a: 61, in key

**Type data.** Syntypes (Geneva Museum, Warsaw Museum)

**Notes.** A detailed morphological description was provided by Haag-Rutenberg (1871b: 29).

*rotundicollis* Haag-Rutenberg, 1871b: 69

**Type data.** Holotype (Munich Museum – Haag-Rutenberg coll.)

*rufofasciatus* Haag-Rutenberg, 1871a: 96

**Type data.** Syntypes (Cape Museum, Warsaw Museum)

*rufonervosus* Haag-Rutenberg, 1871a: 96

**Type data.** Holotype (Munich Museum – Haag-Rutenberg coll.)

*rufostriatus* Haag-Rutenberg, 1875: 70

**Type data.** Syntypes (British Museum, Cape Museum, Munich Museum – Haag-Rutenberg coll.)

*rugulosipennis* Haag-Rutenberg, 1871a: 98

**Type data.** Syntypes (Munich Museum – Haag-Rutenberg coll.)

*rugulosus* (Solier, 1843: 93) *Phanerotomea* [Haag-Rutenberg, 1871b: 49]

**Type data.** Holotype (Paris Museum)

= *Psammodesexilis* Péringuey, 1899: 280 [syn. by Péringuey (1904: 297)]

**Type data.** Syntypes (Cape Museum)

**Notes.** Interpreted as a synonym of *Psammodescaraboides* Haag-Rutenberg, 1871 by Gebien (1937a); however, no justification was provided. It is unclear if Gebien (1937a) was aware of Péringuey’s (1904) interpretation. A detailed morphological investigation of the type material is needed to resolve the status of these species. Presently, this catalogue favours the older interpretation of Péringuey (1904).

*rusticus* Péringuey, 1899: 270

**Type data.** Syntypes (Cape Museum)

*scaber* (Fabricius, 1775: 251) *Pimelia* [Haag-Rutenberg, 1871a: 109]

**Type data.** Holotype (British Museum)

*scabratusscabratus* (Solier, 1843: 74) *Moluris* [Haag-Rutenberg, 1871a: 110]

**Type data.** Holotype (Warsaw Museum – Dupont collection)

*scabratusgariepinus* Koch, 1953c: 5

**Type data.** Holotype (Ditsong Museum) and paratypes (Cape Museum, Ditsong Museum)

*scabriusculus* Haag-Rutenberg, 1871a: 98

**Type data.** Syntypes (Brussels Museum)

*schultzei* Peinguey, 1908: 408

**Type data.** Holotype (Cape Museum)

*segnis* Haag-Rutenberg, 1871a: 71, in key

**Type data.** Syntypes (Vienna Museum)

**Notes.** A detailed morphological description was provided by Haag-Rutenberg (1871b: 49).

*sellatussellatus* Haag-Rutenberg, 1875: 72

**Type data.** Syntypes (Munich Museum – Haag-Rutenberg coll.)

*sellatusuriai* Koch, 1953b: 75

**Type data.** Holotype (Ditsong Museum)

*semipilosus* Haag-Rutenberg, 1871a: 80

**Type data.** Syntypes (Geneva Museum)

= *Psammodesapproximans* Péringuey, 1899: 270 [syn. by Gebien (1937a: 765)]

**Type data.** Holotype (Cape Museum)

*semivillosus* Haag-Rutenberg, 1871a: 80

**Type data.** Syntypes (Munich Museum – Haag-Rutenberg coll.)

*setipennis* Haag-Rutenberg, 1871a: 107

**Type data.** Syntypes (Munich Museum – Haag-Rutenberg coll.)

*solitarius* Péringuey, 1899: 273

**Type data.** Holotype (Cape Museum)

*spiculosus* Haag-Rutenberg, 1871a: 111

**Type data.** Holotype (Munich Museum – Haag-Rutenberg coll.)

= *Psammodeskarrooensis* Péringuey, 1899: 267 [syn. by Gebien (1937a: 760)]

**Type data.** Syntypes (Cape Museum)

*spinosus* Haag-Rutenberg, 1871a: 62, in key

**Type data.** Holotype (Cape Museum)

**Notes.** A detailed morphological description was provided by Haag-Rutenberg (1871b: 40).

*splendens* Haag-Rutenberg, 1871a: 73, in key

**Type data.** Syntypes (British Museum, Munich Museum – Haag-Rutenberg coll.)

**Notes.** A detailed morphological description was provided by Haag-Rutenberg (1871b: 61).

*steinhelli* Haag-Rutenberg, 1878: 91

**Type data.** Holotype (Munich Museum – Haag-Rutenberg coll.)

*striatopilosus* Haag-Rutenberg, 1871a: 90

**Type data.** Syntypes (Geneva Museum)

*subaeneus* Harold, 1878: 106

**Type data.** Holotype (Munich Museum)

*subcostatus* (Solier, 1843: 88) *Phanerotomea* [Haag-Rutenberg, 1871b: 46]

**Type data.** Holotype (British Museum)

*subgranulatus* Haag-Rutenberg, 1871a: 78

**Type data.** Syntypes (Cape Museum, Munich Museum – Haag-Rutenberg coll.)

*tenuipes* (Fåhraeus, 1870: 273) *Hypomelus* [Haag-Rutenberg, 1871b: 47]

**Type data.** Holotype (Cape Museum)

*timarchoides* Haag-Rutenberg, 1871a: 79

**Type data.** Holotype (Munich Museum – Haag-Rutenberg coll.)

*togatus* Koch, 1953c: 10

**Type data.** Holotype (Ditsong Museum) and paratypes (Ditsong Museum, Lund University)

*tomentosus* (Solier, 1843: 73) *Moluris* [Haag-Rutenberg, 1871a: 93]

**Type data.** Holotype (Paris Museum)

*trachysceloides* Haag-Rutenberg, 1871a: 72, in key

**Type data.** Syntypes (British Museum, Munich Museum – Haag-Rutenberg coll.)

**Notes.** A detailed morphological description was provided by Haag-Rutenberg (1871b: 55).

*transvaalensis* Haag-Rutenberg, 1875: 81

**Type data.** Syntypes (British Museum, Munich Museum – Haag-Rutenberg coll.)

=*Psammodeslaetulus* Péringuey, 1899: 278 [syn. by Gebien (1937a: 766)]

**Type data.** Syntypes (Cape Museum)

*tricostatus* (Fåhraeus, 1870: 273) *Hypomelus* [Haag-Rutenberg, 1871b: 48]

**Type data.** Syntypes (Naturhistoriska riksmuseet)

= *Psammodesmendax* Péringuey, 1899: 283 [syn. by Gebien (1937a: 766)]

**Type data.** Holotype (Cape Museum)

= *Psammodespraestans* Péringuey, 1899: 282 [syn. by Péringuey (1904: 297)]

**Type data.** Syntypes (Cape Museum)

**Notes.** Interpreted as a synonym of *Hypomelustenuipes* Fåhraeus, 1870 by Gebien (1937a); however, no justification was provided. It is not clear if Gebien (1937a) was aware of Péringuey’s (1904) interpretation. A detailed morphological investigation of the type material of *tenuipes*, *tricostatus*, and its synonyms, is needed in order to resolve the status of these species. Presently, this catalogue favours the older interpretation of Péringuey (1904).

*tristis* Fåhraeus, 1870: 269

**Type data.** Syntypes (Naturhistoriska riksmuseet)

*tuberculipennis* Haag-Rutenberg, 1871a: 60, in key

**Type data.** Holotype (British Museum)

**Notes.** A detailed morphological description was provided by Haag-Rutenberg (1871b: 31).

= *Psammodesinterventor* Péringuey, 1908: 410 [syn. by Péringuey (1908: 504) in erratum of the original work]

**Type data.** Holotype (Cape Museum)

**Notes.**Gebien (1937a) treated *tuberculipennis* and *interventor* as two independent species; however, no comments were provided. It is possible that Gebien missed Péringuey’s (1908) erratum. A detailed investigation of the type specimens is needed to solve this taxonomic problem. Presently, this catalogue favours the older interpretation of Péringuey (1908).

*tumidipennis* Haag-Rutenberg, 1871a: 88

**Type data.** Syntypes (Geneva Museum, Warsaw Museum – Dohrn coll.)

*undulatus* Haag-Rutenberg, 1871a: 102

**Type data.** Holotype (Munich Museum – Haag-Rutenberg coll.)

*uniformisuniformis* Haag-Rutenberg, 1871a: 88

**Type data.** Holotype (Munich Museum – Haag-Rutenberg coll.)

*uniformisuniformis* Koch, 1953c: 7

**Type data.** Holotype (Cape Museum) and paratypes (Ditsong Museum, Lund University, McGregor Museum, Rhodes University)

*uniformisrugigaster* Koch, 1953c: 7

**Type data.** Holotype (Cape Museum) and paratypes (Cape Museum, Ditsong Museum, Stellenbosch University, Lund University)

*validus* Kratz, 1897: 48

**Type data.** Holotype (Berlin Museum)

*velutinus* Haag-Rutenberg, 1871a: 87

**Type data.** Syntypes (Vienna Museum)

*ventricosus* Fåhraeus, 1870: 264

**Type data.** Syntypes (Naturhistoriska riksmuseet)

*vialistuberculifer* Haag-Rutenberg, 1871a: 60, in key

**Type data.** Syntypes (Munich Museum – Haag-Rutenberg coll.)

**Notes.** A detailed morphological description was provided by Haag-Rutenberg (1871b: 31).

*vialisvialis* (Burchell, 1822: 305) *Moluris* [Gebien, 1937a: 760]

**Type data.** Syntypes (Oxford University – Burchell coll.)

= *Molurispierreti* Amyot, 1835: 129 [syn. by Gebien (1937a: 760)]

**Type data.** Syntypes (Paris Museum)

*villosostriatus* Haag-Rutenberg, 1871a: 87

**Type data.** Holotype (Munich Museum – Haag-Rutenberg coll.)

*villosulus* Haag-Rutenberg, 1871a: 81

**Type data.** Syntypes (Munich Museum – Haag-Rutenberg coll.)

*vittatus* (Solier, 1843: 113) *Trachynotus* [Haag-Rutenberg, 1871a: 85]

**Type data.** Holotype (Paris Museum)

*volvulus* Haag-Rutenberg, 1871a: 68, in key

**Type data.** Holotype (Naturhistoriska riksmuseet)

**Notes.** A detailed morphological description was provided by Haag-Rutenberg (1871b: 37).

=*Psammodesadventitus* Péringuey, 1899: 299 [syn. by Péringuey, (1904: 297)]

**Type data.** Holotype (Cape Museum)

*zschokkei* Koch, 1953b: 84

**Type data.** Holotype (Ditsong Museum) and paratypes (Cape Museum, Ditsong Museum)

##### Genus *Psammophanes* Lesne, 1922: 689

**Type species.***Moluriscatenata* Reiche, 1850 (by original designation)

**Notes.** Originally described as a subgenus of *Psammodes*, and elevated to the generic level by Koch (1953a).

###### Subgenus Psammolophus Koch, 1953a: 154

**Type species.***Psammodesacuticosta* Fairmaire, 1884 (by original designation)

*acuticosta* (Fairmaire, 1884: LXXIV) *Psammodes* [Koch, 1953a: 155]

**Type data.** Syntypes (Basel Museum, Cape Museum)

*lomii* (Gridelli, 1939b: 230) *Psammodes* [Koch, 1953a: 155]

**Type data.** Syntypes (Trieste Museum)

###### Subgenus Psammophanes Lesne, 1922: 689

**Type species.***Moluriscatenata* Reiche, 1850 (by original designation)

*angulicauda* (Lesne, 1922: 691) *Psammodes* [Lesne, 1922: 691]

**Type data.** Syntypes (Paris Museum)

*antinorii* (Gridelli, 1939b: 234) *Psammodes* comb. n.

**Type data.** Syntypes (Trieste Museum)

**Notes.** This species was unknown to Koch (1953a). Included here within the subgenus Psammophanes based on its close affiliation to *raffrayi* (see Gridelli 1939b).

*beccariibeccarii* (Gridelli, 1939a: 105) *Psammodes* [Gridelli, 1939a: 105]

**Type data.** Holotype (Vienna Museum) and paratype (Monaco Museum)

*beccariisudanicus* Koch, 1953a: 169

**Type data.** Holotype (Ditsong Museum) and paratypes (Basel Museum, Ditsong Museum, Munich Museum)

*borosi* Koch, 1953a: 173

**Type data.** Holotype (Brussels Museum) and paratypes (Basel Museum, Brussels Museum, Budapest Museum, Ditsong Museum)

*castanopterus* (Haag-Rutenberg, 1875: 69) *Amiantus* [Gebien 1937a: 763]

**Type data.** Syntypes (British Museum, Munich Museum – Haag-Rutenberg coll.)

*catenatus* (Reiche, 1850: 366) *Moluris* [Lesne, 1922: 692]

**Type data.** Holotype (Paris Museum)

= *Psammodesabyssinicus* Haag-Rutenberg, 1871b: 32 [syn. by Koch (1953a: 171)]

**Type data.** Syntypes (Munich Museum, Warsaw Museum)

*camiadei* Robiche, 2013: 157

**Type data.** Holotype (Paris Museum) and paratypes (Paris Museum, Gérard Robiche collection)

*densepunctatus* Koch, 1953a: 158

**Type data.** Holotype (Munich Museum) and paratypes (Ditsong Museum, Munich Museum)

*duodecimcostatus* (Lesne, 1922: 693) *Psammodes* [Lesne, 1922: 693]

**Type data.** Syntypes (Paris Museum)

**Notes.** Originally described as a subspecies of *catenatus*; status elevated by Koch (1953a).

*granuliger* Koch, 1953a: 160

**Type data.** Holotype (Ditsong Museum) and paratypes (Basel Museum, Ditsong Museum)

*gridelliigridellii* Koch, 1953a: 164

**Type data.** Holotype (Munich Museum)

*gridelliimicrosetosus* Koch, 1953a: 165

**Type data.** Holotype (Munich Museum)

*gurannicus* (Lesne, 1922: 694) *Psammodes* [Lesne, 1922: 694]

**Type data.** Holotype (Paris Museum)

*impressiventris* (Fairmaire, 1897: 115) *Psammodes* [Gebien, 1937a: 762]

**Type data.** Holotype (Basel Museum)

*kilimandjarus* Koch, 1953a: 174

**Type data.** Holotype (Paris Museum) and paratypes (Munich Museum)

*leakeyi* Koch, 1953a: 171

**Type data.** Holotype (Kenya Museum) and paratypes (Ditsong Museum)

*mirei* (Pierre, 1979: 7) *Psammodes* comb. n.

**Notes.** Originally described as Psammodes (Psammophanes) mirei. According to the original description, this species is allied to Psammophanes (Psammophanes) naivashanus (Lesne, 1922). Based on this information, *P.mirei* is hereby included within subgenus Psammophanes.

**Type data.** Holotype and paratypes (Paris Museum)

*nairobiensis* Koch, 1953a: 173

**Type data.** Holotype (Royan Brussels) and paratypes (Basel Museum, Brussels Museum, Budapest Museum, Ditsong Museum, Munich Museum, Kenya Museum)

*naivashanus* (Lesne, 1922: 690) *Psammodes* [Lesne, 1922: 690]

**Type data.** Syntypes (Budapest Museum, Paris Museum)

*pilosiusculusecostatus* (Lesne, 1922: 691) *Psammodes* [Lesne, 1922: 691]

**Type data.** Syntypes (Paris Museum)

*pilosiusculuspilosiusculus* (Gebien, 1913: 60) *Psammodes* [Gebien, 1937a: 763]

**Type data.** Holotype (Munich Museum) and paratypes (Basel Museum, Budapest Museum, Munich Museum, Tervuren Museum)

*pilosiusculusruandanus* Koch, 1953a: 168

**Type data.** Holotype (Tervuren Museum)

*plicatoides* Koch, 1953a: 164

**Type data.** Holotype and paratype (Kenya Museum)

*plicatusaethiopicus* Koch, 1953a: 162

**Type data.** Holotype (Munich Museum)

*plicatusmultilineatus* Koch, 1953a: 161

**Type data.** Holotype (Kenya Museum) and paratypes (Basel Museum, Budapest Museum, Munich Museum, Kenya Museum)

*plicatusplicatus* (Gerstaecker, 1871: 59) *Phrynocolus* [Koch, 1953a: 160]

**Type data.** Syntypes (Berlin Museum)

*plicatussulcatus* (Gebien 1910b, replacement name) *Psammodes* [Lesne, 1922: 694]

= *Psammodesplicipennis* Fairmaire, 1891b: CCXCIII [junior primary homonym of *Psammodesplicipennis* Gemminger, 1870: 1899]

**Type data.** Holotype (Paris Museum)

*praetenuispraetenuis* Koch, 1953a: 163

**Type data.** Holotype (Munich Museum) and paratypes (Basel Museum, Budapest Museum, Ditsong Museum, Munich Museum)

*praetenuissubtomentosus* Koch, 1953a: 163

**Type data.** Holotype (Munich Museum) and paratypes (Budapest Museum, Munich Museum)

*pyriformis* (Gridelli, 1939b) *Psammodes* [Koch, 1953a: 171]

**Type data.** Syntypes (Trieste Museum)

*raffrayipseudocatenatus* Koch, 1953a: 167

**Type data.** Holotype (Ditsong Museum) and paratypes (Basel Museum, Budapest Museum, Ditsong Museum, Munich Museum)

*raffrayiraffrayi* (Lesne, 1922: 694) *Psammodes* [Lesne, 1922: 694]

**Type data.** Syntypes (Paris Museum)

*rubrolineatus* (Lesne, 1922: 693) *Psammodes* [Lesne, 1922: 693]

**Type data.** Syntypes (Basel Museum)

*sexcostatus* (Gerstäcker, 1884: 54) *Phrynocolus* [Lesne, 1922: 691]

**Type data.** Holotype (Berlin Museum)

*somalicus* Koch, 1953a: 158

**Type data.** Holotype (Munich Museum) and paratype (Disong Museum)

*terrenuscrassecostatus* Koch, 1953a: 175

**Type data.** Holotype (Tervuren Museum) and paratypes (Budapest Museum, Ditsong Museum)

*terrenusrugilineatus* Koch, 1953a: 176

**Type data.** Holotype (Tervuren Museum) and paratypes (National Museums of Kenya)

*terrenusterrenus* (Lesne, 1922: 694) *Psammodes* [Lesne, 1922: 694]

**Type data.** Holotype (Paris Museum)

*vagecostatus* (Fairmaire, 1882b: L) *Psammodes* [Koch 1953a: 169]

**Type data.** Holotype (Paris Museum)

**Notes.** Treated as a synonym of *Amiantuscastanopterus* Haag-Rutenberg, 1875 by Gebien (1937a); however, this interpretation was not accepted by more recent reviewers (e.g., Koch 1953a: 169).

###### Subgenus Psammophrynopsis Koch, 1953a: 157

**Type species.***Phrynocolusfrommi* Wilke, 1921 (by original designation)

*frommi* (Wilke, 1921: 169) *Phrynocolus* [Koch, 1953a: 157]

**Type data.** Syntypes (Berlin Museum)

###### Subgenus Psammophrynus Koch, 1953a: 146

**Type species.**Psammophanes (Psammophrynus) jokli Koch, 1953 (by original designation)

*jokli* Koch, 1953a: 152

**Type data.** Holotype (Brussels Museum) and paratypes (Budapest Museum, Brussels Museum, Ditsong Museum, Munich Museum)

*penicillatuspenicillatus* Koch, 1953a: 153

**Type data.** Holotype (Tervuren Museum)

*penicillatuspiacatus* Koch, 1953a: 154

**Type data.** Holotype (Tervuren Museum)

*poccilator* Koch, 1953a: 153

**Type data.** Holotype (Tervuren Museum)

###### Subgenus Psammostretus Koch, 1953a: 145

**Type species.***Psammodesbisbicostatus* Gebien, 1910 (by original designation)

*bisbicostatusbisbicostatus* (Gebien, 1910a: 153) *Psammodes* [Koch, 1953a: 147]

**Type data.** Syntypes (Basel Museum, Tervuren Museum)

*bisbicostatusleleupi* Koch, 1953a: 148

**Type data.** Holotype (Brussels Museum) and paratypes (Basel Museum, Budapest Museum, Ditsong Museum, Tervuren Museum)

*circumscriptus* Koch, 1953a: 151

**Type data.** Holotype (Cape Museum) and paratypes (Basel Museum, Brussels Museum, Budapest Museum, Ditsong Museum)

*erectepilosuserectepilosus* Koch, 1953a: 150

**Type data.** Holotype (Tervuren Museum) and paratypes (Ditsong Museum, Tervuren Museum)

*erectepilosustanganyikanus* Koch, 1953a: 150

**Type data.** Holotype (Munchen Museum)

*maculicollis* Koch, 1953a: 150

**Type data.** Holotype (Brussels Museum) and paratypes (Basel Museum, Budapest Museum, Ditsong Museum, Tervuren Museum)

*neavei* (Gebien, 1910a: 153) *Psammodes* [Koch, 1953a: 149]

**Type data.** Syntypes (Basel Museum, Tervuren Museum)

*punctipilus* Koch, 1953a: 149

**Type data.** Holotype (Brussels Museum) and paratype (Tervuren Museum)

*prosodoides* (Gebien, 1910a: 153) *Psammodes* [Koch, 1953a: 151]

**Type data.** Syntypes (Basel Museum, Cape Museum, Tervuren Museum)

###### Subgenus Psammotyriopsis Koch, 1953a: 144

**Type species.**Psammophanes (Psammotyriopsis) bredoi Koch, 1953 (by original designation)

*bredoi* Koch, 1953a: 144

**Type data.** Holotype (Budapest Museum) and paratype (Budapest Museum, Royal Brusseles)

###### Subgenus Somalarabes Koch, 1953a: 155

**Type species.***Psammodesgracilentus* Fairmaire, 1882 (by original designation)

*ahlmedoensis* Koch, 1969: 31

**Type data.** Holotype and paratypes (Munich Museum)

*arabicus* (Gebien, 1938: 58, in Schuster & Gebien, 1938) *Psammodes* [Koch, 1953a: 155]

**Type data.** Syntypes (Hamburg University)

*benardellii* Koch, 1965: 126

**Type data.** Holotype (Milan Museum)

*gracilentus* (Fairmaire, 1882a: 69) *Psammodes* [Koch, 1953a: 156]

**Type data.** Syntypes (Paris Museum)

*hemmingi* Koch, 1969: 25

**Type data.** Holotype (Munich Museum) and paratypes (Ditsong Museum)

*nogalus* Koch, 1962c: 242

**Type data.** Holotype (Milan Museum)

##### Genus *Psammotyria* Koch, 1953a: 137

**Type species.***Psammodesertli* Kolbe, 1904 (by original designation)

**Notes.** Originally described as a subgenus of *Psammodes*. Elevated to generic level by Koch (1955).

*attenuatusattenuatus* (Fairmaire, 1887: 180) *Moluris* [Koch, 1953a: 142]

**Type data.** Holotype (Paris Museum)

= *Moluristentyrioides* Fairmaire, 1891a: 249 [syn. by Koch 1953a: 142]

**Type data.** Holotype (Paris Museum)

*attenuatusmagnophthalmus* (Koch, 1953a: 143) *Psammodes* [Koch, 1953a: 143]

**Type data.** Syntypes (Basel Museum, Ditsong Museum, Munich Museum)

*ertliertli* (Kolbe, 1904: 301) *Psammodes* [Koch, 1955]

**Type data.** Syntypes (Basel Museum, Berlin Museum, Tervuren Museum)

*ertlipunctativentris* (Koch, 1953a: 143) *Psammodes* [Koch, 1953a: 143]

**Type data.** Holotype (Budapest Museum)

*ertlispinosocostatus* (Kolbe, 1904: 302) *Psammodes* [Koch, 1955]

**Type data.** Syntypes (Berlin Museum)

*lateridenslateridens* (Fairmaire, 1887: 179) *Moluris* [Koch, 1953a: 143]

**Type data.** Holotype (Paris Museum)

*lateridensnyassicus* (Koch, 1953a: 143) *Psammodes* [Koch, 1953a: 143]

**Type data.** Holotype (Budapest Museum)

*quadriplicatus* (Gebien, 1910b: 159, replacement name) *Psammodes* [Koch, 1953a: 141]

= *Psammodesquadricostatus* Fairmaire, 1891b: CCXCIII [junior secondary homonym of *Hypomelusquadricostatus* Fåhraeus, 1870: 272]

**Type data.** Holotype (Paris Museum)

#### Subtribe Oxurina Koch, 1955: 34

**Type genus.***Oxura* Kirby, 1819

**Taxonomic diversity.** (8 gen., 63 spp.): *Decoriplus* (11 ssp.), *Miripronotum* (1), *Namibomodes* (4), *Oxura* (9), *Palpomodes* (4), *Pterostichula* (17), *Stenethmus* (11), *Synhimba* (6).

**Distribution.** The majority of species were described from Namibia. A small number of species of *Stenethmus* were described from the northern part of Tanzania, while some species of *Decoriplus* from Central Africa (Fig. 51).

##### Genus *Decoriplus* Louw, 1979: 120

**Type species.***Psammodespictus* Haag-Rutenberg, 1871 (by original designation)

*aequabilis* Louw, 1979: 125

**Type data.** Holotype (Ditsong Museum) and paratypes (Ditsong Museum and Windhoek Museum)

*clavus* Louw, 1979: 126

**Type data.** Holotype and paratypes (Ditsong Museum)

*convexus* Louw, 1979: 127

**Type data.** Holotype (Ditsong Museum)

*costimargo* Louw, 1979: 128

**Type data.** Holotype (Windhoek Museum) and paratypes (Budapest Museum, Cape Museum, Ditsong Museum, Windhoek Museum)

*discicollis* Louw, 1979: 130

**Type data.** Holotype and paratype (Ditsong Museum)

*granulimargo* Louw, 1979: 131

**Type data.** Holotype (Ditsong Museum) and paratypes (Ditsong Museum, Windhoek Museum)

*hamatus* Louw, 1979: 133

**Type data.** Holotype (British Museum) and paratypes (British Museum, New York Museum)

*aequabilishieroglyphicus* (Haag-Rutenberg, 1871a: 69, in key) *Psammodes* [Louw, 1979: 134]

**Type data.** Lectotype, designated by Louw (1979) (Naturhistoriska riksmuseet) and paralectotypes (Ditsong Museum, Naturhistoriska riksmuseet, Warsaw Museum)

**Notes.**A detailed morphological description was provided by Haag-Rutenberg (1871b: 81).

*humerus* Louw, 1979: 136

**Type data.** Holotype (Ditsong Museum) and paratypes (British Museum, Ditsong Museum, Paris Museum, Windhoek Museum)

*pictus* (Haag-Rutenberg, 1871a: 69, in key) *Psammodes* [Louw, 1979: 122]

**Notes.** A detailed morphological description was provided by Haag-Rutenberg (1871b: 80).

**Type data.** Holotype (Naturhistoriska riksmuseet)

*striatulus* Louw, 1979: 138

**Type data.** Holotype (Ditsong Museum) and paratypes (Budapest Museum, Ditsong Museum, Paris Museum, Windhoek Museum)

##### Genus *Miripronotum* Louw, 1979: 118

**Type species.***Miripronotumprominoculatum* Louw, 1979 (by original designation)

*prominoculatum* Louw, 1979: 119

**Type data.** Holotype and paratypes (Windhoek Museum)

##### Genus *Namibomodes* Koch, 1952: 223

**Type species.***Psammodesserrimargo* Gebien, 1938 (by original designation)

*maculicollis* Koch, 1962b: 111

**Type data.** Holotype (Ditsong Museum) and paratypes (Basel Museum, Budapest Museum, Ditsong Museum)

*rubra* Koch, 1962b: 112

**Type data.** Holotype and paratype (Ditsong Museum)

*serrimargo* (Gebien, 1938a: 86) *Psammodes* [Koch, 1952: 221]

**Type data.** Syntypes (Basel Museum, Bremen Museum, Cape Museum)

*zarcoi* Koch, 1962b: 110

**Type data.** Holotype (Ditsong Museum) and paratypes (Budapest Museum, Ditsong Museum)

##### Genus *Oxura* Kirby, 1819: 413

**Type species.***Oxurasetosa* Kirby, 1819 (by monotypy)

= *Oxyura* Agassiz, 1846: 267 [junior homonym of *Oxyura* Bonaparte, 1831 (Aves: Anatidae)]

**Type species.***Oxurasetosa* Kirby, 1819 (by monotypy)

**Notes.** Unjustified emendation of *Oxura* Kirby, 1819.

*connexa* (Haag-Rutenberg, 1871a: 46, in key) *Psammodes* [Louw, 1979: 159]

**Type data.** Holotype (Paris Museum) and paratypes (Cape Museum, Paris Museum)

**Notes.** A detailed morphological description was provided by Haag-Rutenberg (1871b: 35).

*femoralis* Haag-Rutenberg, 1871b: 111

**Type data.** Lectotype, designated by Louw (1979) and praralectotype (Munich Museum)

*margoabsolutamargoabsoluta* Louw, 1979: 164

**Type data.** Holotype (Ditsong Museum) and paratypes (Ditsong Museum, Windhoek)

*margoabsolutapuncticollis* Louw, 1979: 165

**Type data.** Holotype (Windhoek Museum) and paratypes (Pretoria University, Windhoek Museum)

*punctipennis* Haag-Rutenberg, 1871b: 111

**Type data.** Lectotype, designated by Louw (1979) (British Museum)

*rufotibiatarufotibiata* Louw, 1979: 167

**Type data.** Holotype (Windhoek Museum) and paratypes (Ditsong Museum and Windhoek Museum)

*rufotibiataplanipennata* Louw, 1979: 168

**Type data.** Holotype and paratypes (Windhoek Museum)

*setosa* Kirby, 1819: 414

**Type data.** Lectotype, designated by Louw (1979) (Geneva Museum) and paralectotype (British Museum)

*vestita* Solier, 1843: 119

**Type data.** Holotype (Torino Museum)

##### Genus *Palpomodes* Koch, 1952: 223

**Type species.***Psammodesphysopterus* Gebien, 1920 (by monotypy)

**Notes.** Originally described as a subgenus of *Namibomodes*. Elevated to the generic level by Koch (1962b).

###### Subgenus Palpomodes Koch, 1952: 223

**Type species.***Psammodesphysopterus* Gebien, 1920 (by monotypy)

*halophilus* (Koch, 1958:57) *Namibomodes* [Koch, 1958: 58]

**Type data.** Syntypes (Basel Museum, Budapest Museum, Ditsong Museum)

*physopterusangolensis* (Koch, 1958: 58) (*Namibomodes*) [Koch, 1958: 58]

**Type data.** Holotype (Ditsong Museum)

*physopterusphysopterus* (Gebien, 1920) *Psammodes* [Koch, 1952: 223]

**Type data.** Holotype (Hamburg University – Michaelsen coll.)

###### Subgenus Pygmaeodes Koch, 1952: 223

**Type species.***Namibomodesrudebecki* Koch, 1952 (by monotypy)

**Notes.** Originally described as a subgenus of *Namibomodes*.

*rudebecki* (Koch, 1952: 223) *Namibomodes* [Koch, 1952: 223]

**Type data.** Holotype (Lund University)

##### Genus *Pterostichula* Koch, 1952: 224

**Type species.**Pterostichula (Pterostichula) calathoides Koch, 1952 (by original designation)

###### Subgenus Pterostichula Koch, 1952: 224

**Type species.**Pterostichula (Pterostichula) calathoides Koch, 1952 (by original designation)

*aridipaludis* Louw, 1979: 146

**Type data.** Holotype (Cape Museum) and paratype (Cape Museum, Windhoek)

*broomoides* Koch, 1952: 228

**Type data.** Holotype (Ditsong Museum) and paratypes (Cape Museum, Ditsong Museum)

*calathoides* Koch, 1952: 227

**Type data.** Holotype (Cape Museum) and paratypes (Agricultural Institute, Basel Museum, Budapest Museum, Cape Museum, Ditsong Museum)

*diaphana* Louw, 1979: 145

**Type data.** Holotype (Ditsong Museum) and paratypes (Budapest Museum, Ditsong Museum, Windhoek Museum)

*dubia* Louw, 1979: 141

**Type data.** Holotype (Windhoek Museum) and paratype (Cape Museum, Windhoek Museum)

*ellamariae* Koch, 1952: 229

**Type data.** Holotype (Ditsong Museum) and paratypes (Basel Museum, Budapest Museum, Cape Museum, Ditsong Museum, Munich Museum)

*infuscata* Koch, 1952: 227

**Type data.** Holotype (Ditsong Museum)

*kung* Koch, 1952: 229

**Type data.** Holotype and paratype (Ditsong Museum)

*namaqua* Koch, 1952: 228

**Type data.** Holotype (Ditsong Museum)

*quarzophila* Koch, 1952: 227

**Type data.** Holotype (Ditsong Museum) and paratypes (Ditsong Museum, Munich Museum)

*solitudo* Louw, 1979: 147

**Type data.** Holotype (Windhoek Museum) and paratype (Cape Museum, Windhoek Museum)

###### Subgenus Ripicolodes Koch, 1952: 225

**Type species.**Pterostichula (Ripicolodes) misanthropa Koch, 1952 (by original designation)

*arenicola* Koch, 1952: 232

**Type data.** Holotype (Ditsong Museum)

*frontalis* Koch, 1952: 232

**Type data.** Holotype (Ditsong Museum)

*misanthropamisanthropa* Koch, 1952: 231

**Type data.** Holotype (Ditsong Museum) and paratypes (Cape Museum, Ditsong Museum, Munich Museum)

*misanthropakunenensis* Koch, 1952: 231

**Type data.** Holotype and paratype (Ditsong Museum)

*omurambestris* Koch, 1952: 230

**Type data.** Holotype (Ditsong Museum)

*parvicollis* Louw, 1979: 153

**Type data.** Holotype (Ditsong Museum) and paratypes (Cape Museum, Ditsong Museum, Windhoek Museum)

##### Genus *Stenethmus* Gebien, 1937b: 41

**Type species.***Psammodestentyriiniformis* Hesse, 1935 (by original designation)

**Notes.** Classified within Tentyriini by Ferrer (2004b); however, no justification for this interpretation was proposed.

*borealis* Kaszab, 1972: 231

**Type data.** Holotype (Budapest Museum)

*impuncticollis* Gebien, 1937b: 42

**Type data.** Holotype and paratype (Basel Museum)

*massaicus* Kaszab, 1972: 231

**Type data.** Holotype (Budapest Museum)

*orientalis* Kaszab, 1972: 232

**Type data.** Holotype (Budapest Museum)

*poggii* Ferrer, 2004b: 513

**Type data.** Holotype (Geneva Museum)

*punctipleuris* Kaszab, 1972: 233

**Type data.** Holotype and paratype (Budapest Museum)

*punctiventris* Genien, 1937b: 43

**Type data.** Syntypes (Basel Museum)

*rhodesianus* Kaszab, 1972: 232

**Type data.** Holotype (Budapest Museum)

*szunyoghyi* Kaszab, 1972: 230

**Type data.** Holotype and paratype (Budapest Museum)

*tentyriiniformistentyriiniformis* (Hesse, 1935: 546) *Psammodes* [Gebien, 1937b: 41]

**Type data.** Holotype (Ditsong Museum) and paratypes (Cape Museum, Ditsong Museum)

*tentyriiniformisseptentrionalis* Gebien, 1937b: 44

**Type data.** Syntypes (Basel Museum, Budapest Museum)

##### Genus *Synhimba* Koch, 1952: 216

**Type species.***Psammodescordiformis* Haag-Rutenberg, 1871 (by original designation)

*cordiforme* (Haag-Rutenberg, 1871a: 62, in key) *Psammodes* [Koch, 1952: 219]

**Type data.** Syntypes (Naturhistoriska riksmuseet)

**Notes.** A detailed morphological description was provided by Haag-Rutenberg (1871b: 77).

*hyalinumhyalinum* Koch, 1952: 220

**Type data.** Holotype (Ditsong Museum) and paratypes (Basel Museum, Budapest Museum, California Academy, Ditsong Museum, Munich Museum)

*hyalinumovambo* Koch, 1952: 220

**Type data.** Holotype (Ditsong Museum) and paratypes (Basel Museum, California Academy, Ditsong Museum, Munich Museum)

*melancholica* (Haag-Rutenberg, 1871a: 69, in key) *Psammodes* [Koch, 1952: 218]

**Type data.** Holotype (Munich Museum – Haag-Rutenberg coll.)

**Notes.** A detailed morphological description was provided by Haag-Rutenberg (1871b:79).

*pruinosum* Koch, 1952: 219

**Type data.** Holotype (Ditsong Museum) and paratypes (Barcelona Museum, Basel Museum, British Museum, Budapest Museum, Ditsong Museum, Munich Museum)

*sculpturatum* (Haag-Rutenberg, 1871a: 62, in key) *Psammodes* [Koch, 1952: 219]

**Type data.** Holotype (Munich Museum – Haag-Rutenberg coll.)

**Notes.** A detailed morphological description was provided by Haag-Rutenberg (1871b: 78).

#### Subtribe Phanerotomeina Koch, 1958: 58

**Type genus.***Phanerotomea* Koch, 1958 [junior objective synonym proposed of *Ocnodes*]

**Taxonomic diversity.** (5 gen., 177 spp.): *Huilamus* (1 sp.), *Ocnodes* (149), *Psammoryssus* (1), *Stridulomus* (1), *Tarsocnodes* (25).

**Distribution.** Widely distributed in the southern part of the Afrotropical Realm. Only two species, *Ocnodesgridellii* (Koch, 1960) and *O. humerangula* (Koch, 1952), were described north from the equator. None of the known species were reported from the Eastern Cape (Fig. 51).

##### Genus *Huilamus* Koch, 1953b: 79

**Type species.***Huilamuswelwitschi* Koch, 1953 (by original designation)

*welwitschi* Koch, 1953b: 80

**Type data.** Holotype (Ditsong Museum) and paratypes (British Museum, Ditsong Museum, Munich Museum)

##### Genus *Ocnodes* Fåhraeus, 1870: 270

**Type species.***Ocnodesscrobicollis* Fåhraeus, 1870 (**here designated**)

###### Subgenus Chiliarchum Koch, 1954b: 263

**Type species.**Moluris (Phanerotoma) bertolonii Guérin-Méneville, 1844 (by original designation)

*arnoldiarnoldi* (Koch, 1952: 313) *Phanerotomea* comb. n.

**Type data.** Holotype (Ditsong Museum) and paratypes (Ditsong Museum, Durban Museum, Rhodesia Museum)

*arnoldisabianus* (Koch, 1952: 314) *Phanerotomea* comb. n.

**Type data.** Holotype (Ditsong Museum)

*bertolonii* (Guérin-Méneville, 1844: 148) *Moluris* [Koch, 1952: 314], comb. n.

**Type data.** Syntypes (Paris Museum)

**Notes.** While describing this species, Guérin-Méneville (1844) used two forms of the name: *bertolinii* and *bertolonii*. Bertoloni (1849), the first reviewer, selected the second one.

*freyi* (Koch, 1952: 316) *Phanerotomea* comb. n.

**Type data.** Holotype (Ditsong Museum)

*gueriniguerini* (Haag-Rutenberg, 1871a: 71, in key) *Psammodes* [Koch, 1952: 314], comb. n.

**Type data.** Syntypes (Geneva Museum)

**Notes.** A detailed morphological description was provided by Haag-Rutenberg (1871b: 82).

*guerinilawrencii* (Koch, 1954b: 265) *Phanerotomea* comb. n.

**Type data.** Holotype (Cape Museum) and paratypes (Cape Museum)

*guerinimancus* (Koch 1954b: 264) *Phanerotomea* comb. n.

**Type data.** Holotype (Maputo Museum)

*junodi* (Péringuey, 1899: 275) *Psammodes* [Koch, 1952: 314], comb. n.

**Type data.** Holotype (Cape Museum)

= *Psammodesjunodi* Fairmaire, 1899a: 179 [syn. by Péringuey (1904: 297)]

**Type data.** Holotype (Paris Museum)

###### Subgenus Ocnodes Fåhraeus, 1870: 270

**Type species.***Ocnodesscrobicollis* Fåhraeus, 1870 (here designated)

**Notes.** This genus-group name was treated as a synonym of *Psammodes* (see Champion 1895: 81, Gebien 1910b: 154), and later as a subgenus of that genus (e.g., Gebien 1937a). Subsequently, Koch (1958) included the majority of *Ocnodes* (sensu Gebien, 1937a: 769) species (including the newly designated type species) within *Phanerotomea*. However, the synonymy between *Ocnodes* and *Phanerotomea* was never officially proposed.

= *Phanerotoma* Solier, 1843: 82 [junior homonym of *Phanerotoma* Wesmael 1838: 695 (Insecta: Hymenoptera)]

= *Phanerotomea* Koch, 1958: 58, syn. n., replacement name

**Type species.***Phanerotomaelongatum* Solier, 1843 (by original designation)

*acuductusacuductus* (Ancey, 1883: 118) *Psammodes* comb. n.

**Type data.** Syntypes (Paris Museum)

*acuductusufipanus* (Koch, 1952: 301) *Phanerotomea* comb. n.

**Type data.** Holotype (Munich Museum) and paratypes (Ditsong Museum, Munich Museum, Tervuren Museum)

*adamantinus* (Koch, 1952: 245) *Phanerotomea* comb. n.

**Type data.** Holotype (Cape Museum)

*argenteofasciatus* (Koch, 1953b: 82) *Phanerotomea* comb. n.

**Type data.** Holotype (Ditsong Museum)

*barbosai* (Koch, 1952: 317), comb. n.

**Type data.** Holotype (Ditsong Museum) and paratypes (Basel Museum, Cape Museum, Maputo Museum)

*basilewskyi* (Koch, 1952: 308), comb. n.

**Type data.** Holotype (Tervuren Museum)

*bellmarleyi* (Koch, 1952: 305), comb. n.

**Type data.** Holotype (Ditsong Museum) and paratypes (Basel Museum, British Museum, Cape Museum, Ditsong Museum, Durban Museum)

*benguelensis* (Koch, 1952: 276) *Phanerotomea* comb. n.

**Type data.** Holotype (Cape Museum) and paratype (Basel Museum, Cape Museum)

*blandus* (Koch, 1952: 291) *Phanerotomea* comb. n.

**Type data.** Holotype (Tervuren Museum) and paratypes (Basel Museum, Ditsong Museum, Tervuren Museum)

*brevicornis* (Haag-Rutenberg, 1875: 79) *Psammodes* comb. n.

**Type data.** Holotype (Munich Museum – Haag-Rutenberg coll.)

= *Psammodesrugicollis* Kolbe, 1883: 23 [syn. by Koch 1952: 295]

**Type data.** Syntypes (Berlin Museum)

*brunnescensbrunnescens* (Haag-Rutenberg, 1871a: 72, in key) *Psammodes* comb. n.

**Type data.** Syntypes (Ditsong Museum, Munich Museum, Warsaw Museum, former Dohrn coll.)

**Notes.** A detailed morphological description was provided by Haag-Rutenberg (1871b: 65).

Type deposition information after Koch (1952).

*brunnescensmolestus* (Haag-Rutenberg, 1875: 75) *Psammodes* comb. n.

**Type data.** Holotype (Munich Museum – Haag-Rutenberg coll.)

*buccinator* (Koch, 1952: 295) *Phanerotomea* comb. n.

**Type data.** Holotype (Brussels Museum) and paratypes (Basel Museum, Ditsong Museum, Brussels Museum, Tervuren Museum)

*bushmanicus* (Koch, 1952: 257) *Phanerotomea* comb. n.

**Type data.** Holotype (Ditsong Museum)

*carbonarius* (Gerstaecker, 1854: 532) *Phanerotomea* comb. n.

**Type data.** Syntypes (Berlin Museum)

*cardiopterus* (Fairmaire, 1888b: 259) *Psammodes* comb. n.

**Type data.** Syntypes (Leiden Museum)

*cataractus* (Koch, 1952: 290) *Phanerotomea* comb. n.

**Type data.** Holotype (Rhodesia Museum)

*cinerarius* (Koch, 1952: 272) *Phanerotomea* comb. n.

**Type data.** Holotype (Basel Museum) and paratype (Basel Museum, Ditsong Museum)

*complanatus* (Koch, 1952: 299) *Phanerotomea* comb. n.

**Type data.** Holotype (Royan Brussels) and paratypes (Basel Museum, Ditsong Museum, Brussels Museum)

*concinnus* Fåhraeus, 1870

**Type data.** Syntypes (Geneva Museum)

**Notes.** Type deposition information after Haag-Rutenberg (1781b).

*confertus* (Koch, 1952: 275) *Phanerotomea* comb. n.

**Type data.** Holotype (Tervuren Museum) and paratypes (Basel Museum, Ditsong Museum, Tervuren Museum, Royal Museum)

*congruens* (Péringuey, 1899: 281) *Psammodes* comb. n.

**Type data.** Holotype (Cape Museum)

*cordiventris* (Haag-Rutenberg, 1871a: 74, in key) *Psammodes* comb. n.

**Type data.** Syntypes (Munich Museum – Haag-Rutenberg coll.)

**Notes.** A detailed morphological description was provided by Haag-Rutenberg (1871b: 71).

*crocodilinus* (Koch, 1952: 311) *Phanerotomea* comb. n.

**Type data.** Holotype (Ditsong Museum)

*dimorphus* (Koch, 1952: 297) *Phanerotomea* comb. n.

**Type data.** Holotype (Basel Museum) and paratypes (Basel Museum, Ditsong Museum)

*distinctus* (Haag-Rutenberg, 1871a: 62, in key) *Psammodes* comb. n.

**Type data.** Syntypes (Basel Museum, British Museum)

**Notes.** A detailed morphological description was provided by Haag-Rutenberg (1871b: 44).

*dolosus* (Péringuey, 1899: 291) *Psammodes* comb. n.

**Type data.** Holotype (Cape Museum)

*dorsocostatus* (Gebien, 1910a: 153) *Psammodes* comb. n.

**Type data.** Syntypes (Basel Museum, Tervuren Museum)

*dubiosus* (Péringuey, 1899: 287) *Psammodes* comb. n.

**Type data.** Holotype (Cape Museum)

*ejectus* (Koch, 1952: 239) *Phanerotomea* comb. n.

**Type data.** Holotype (Ditsong Museum) and paratypes (Basel Museum, Cornell University, Ditsong Museum)

*epronoticus* (Koch, 1952: 246) *Phanerotomea* comb. n.

**Type data.** Holotype (Cape Museum)

*erichsoni* (Haag-Rutenberg, 1871b: 63) *Psammodes* comb. n.

**Type data.** Syntypes (Munich Museum – Haag-Rutenberg coll.)

*ferreiraeferreirae* (Koch, 1952: 238) *Phanerotomea* comb. n.

**Type data.** Holotype (Ditsong Museum) and paratypes (Agricultural Institute, Basel Museum, British Museum, California Academy, Cape Museum, Ditsong Museum)

*ferreiraezulu* (Koch, 1952: 239) *Phanerotomea* comb. n.

**Type data.** Holotype (Ditsong Museum) and paratypes (Basel Museum, Durban Museum, Munich Museum)

*fettingi* (Haag-Rutenberg, 1875: 77) *Psammodes* comb. n.

**Type data.** Syntypes (Basel Museum)

**Notes.** Type deposition information after Koch (1952).

*fistucans* (Koch, 1952: 260) *Phanerotomea* comb. n.

**Type data.** Holotype and paratypes (Munich Museum – Haag-Rutenberg coll.)

*fraternus* (Haag-Rutenberg, 1875: 80) *Psammodes* comb. n.

**Type data.** Holotype (Munich Museum – Haag-Rutenberg coll.)

*freudei* (Koch, 1952: 303) *Phanerotomea* comb. n.

**Type data.** Holotype (Basel Museum) and paratypes (Basel Museum, Ditsong Museum)

*fulgidus* (Koch, 1952: 294) *Phanerotomea* comb. n.

**Type data.** Holotype (Brussels Museum) and paratype (Basel Museum, Brussels Museum, Ditsong Museum)

*funestus* (Haag-Rutenberg, 1871a: 72, in key) *Psammodes* comb. n.

**Type data.** Holotype (Munich Museum – Haag-Rutenberg coll.)

**Notes.** A detailed morphological description was provided by Haag-Rutenberg (1871b: 79).

*gemmeulus* (Koch, 1952: 287) *Phanerotomea* comb. n.

**Type data.** Holotype (Ditsong Museum) and paratypes (Basel Museum, Ditsong Museum)

*gibberosulus* (Péringuey, 1908: 407) *Psammodes* comb. n.

**Type data.** Syntypes (Cape Museum)

*gibbus* (Haag-Rutenberg, 1879: 292) *Psammodes* comb. n.

**Type data.** Syntypes (British Museum, Munich Museum – Haag-Rutenberg coll.)

= *Psammodesinteger*Péringuey 1899: 276 [syn. by Koch 1952: 309]

**Type data.** Holotype (Cape Museum)

*globosus* (Haag-Rutenberg, 1871a: 74, in key) *Psammodes* comb. n.

**Type data.** Holotype (Munich Museum – Haag-Rutenberg coll.)

**Notes.** A detailed morphological description was provided by Haag-Rutenberg (1871b: 73).

= *Psammodesmyrmidon* Péringuey, 1899: 286 [syn. by Koch 1952: 255]

**Type data.** Holotype (Cape Museum)

*granisterna* (Koch, 1952: 266) *Phanerotomea* comb. n.

**Type data.** Holotype (Tervuren Museum) and paratypes (Basel Museum, Ditsong Museum, Tervuren Museum)

*granulosicollis* (Haag-Rutenberg, 1871a: 74, in key) *Psammodes* comb. n.

**Type data.** Holotype (British Museum)

**Notes.** A detailed morphological description was provided by Haag-Rutenberg (1871b: 77).

*gridellii* (Koch, 1960: 263) *Phanerotomea* comb. n.

**Type data.** Holotype and paratypes (Ditsong Museum)

*haemorrhoidalishaemorrhoidalis* (Koch, 1952: 310) *Phanerotomea* comb. n.

**Type data.** Holotype (Tervuren Museum) and paratypes (Basel Museum, Ditsong Museum, Munich Museum)

*haemorrhoidalissalubris* (Koch, 1952: 311) *Phanerotomea* comb. n.

**Type data.** Holotype (Tervuren Museum) and paratypes (Basel Museum, Ditsong Museum, Munich Museum, Tervuren Museum)

*heydeni* (Haag-Rutenberg, 1871a: 62, in key) *Psammodes* comb. n.

**Type data.** Syntypes (Basel Museum)

**Notes.** A detailed morphological description was provided by Haag-Rutenberg (1871b: 41).

*humeralis* (Haag-Rutenberg, 1871a: 62, in key) *Psammodes* comb. n.

**Type data.** Syntypes (Naturhistoriska riksmuseet)

**Notes.** A detailed morphological description was provided by Haag-Rutenberg (1871b: 39).

*humerangula* (Koch, 1952: 332) *Tarsocnodes* comb. n.

**Type data.** Holotype (Munich Museum – Haag-Rutenberg coll.)

*imbricatus* (Koch, 1952: 256) *Phanerotomea* comb. n.

**Type data.** Holotype (Basel Museum)

*imitatorimitator* (Péringuey, 1899: 289) *Psammodes* comb. n.

**Type data.** Syntypes (Cape Museum)

**Notes.**Koch (1952) designated a variety named “*damara*” of the subspecies *imitatorimitator*, expressly giving it infrasubspecific rank. Therefore, according to art. 45.6.4. of the ICZN (1999) is should not be treated as a subspecies.

*imitatorinvadens* (Koch, 1952: 241) *Phanerotomea* comb. n.

**Type data.** Holotype (Ditsong Museum) and paratypes (Basel Museum, Ditsong Museum)

*inflatus* (Koch, 1952: 275) *Phanerotomea* comb. n.

**Type data.** Holotype (Tervuren Museum) and paratypes (Basel Museum, Brussels Museum, Ditsong Museum, Tervuren Museum)

*janssensi* (Koch, 1952: 292) *Phanerotomea* comb. n.

**Type data.** Holotype (Brussels Museum) and paratypes (Basel Museum, Brussels Museum, Ditsong Museum)

*javeti* (Haag-Rutenberg, 1871a: 74, in key) *Psammodes* comb. n.

**Type data.** Syntypes (Munich Museum – Haag-Rutenberg coll.)

**Notes.** A detailed morphological description was provided by Haag-Rutenberg (1871b: 66).

*kulzeri* (Koch, 1952: 273) *Phanerotomea* comb. n.

**Type data.** Holotype (Cape Museum)

*lacustris* (Koch, 1952: 291) *Phanerotomea* comb. n.

**Type data.** Holotype (Brussels Museum) and paratypes (Ditsong Museum, Tervuren Museum)

*laevigatus* (Olivier, 1795: 15) *Pimelia* comb. n.

**Type data.** Syntypes (Paris Museum)

= *Pimeliamarginata* Herbst, 1799: 54 [syn. by Haag-Rutenberg, 1871b: 59]

**Type data.** Syntypes (Berlin Museum)

= *Phanerotomaelongatum* Solier, 1843: 89 [syn. by Haag-Rutenberg, 1871b: 59]

**Type data.** Syntypes (Paris Museum)

*lanceolatus* (Koch, 1953a: 177) *Phanerotomea* comb. n.

**Type data.** Holotype (Museum Budapest)

*licitus* (Peringuey, 1899: 290) *Psammodes* comb. n.

**Type data.** Syntypes (Cape Museum)

*luctuosus* (Haag-Rutenberg, 1871a: 72, in key) *Psammodes* comb. n.

**Type data.** Holotype (Munich Museum)

**Notes.** A detailed morphological description was provided by Haag-Rutenberg (1871b: 66).

*luxurosus* (Koch, 1952: 276) *Phanerotomea* comb. n.

**Type data.** Holotype (Tervuren Museum), and paratypes (Basel Museum, Ditsong Museum, Tervuren Museum)

*maputoensis* (Koch, 1952: 244) *Phanerotomea* comb. n.

**Type data.** Holotype (Ditsong Museum) and paratypes (Basel Museum, Tervuren Museum)

*marginicollis* (Koch, 1952: 282) *Phanerotomea* comb. n.

**Type data.** Holotype (Cape Museum) and parartype (Ditsong Museum)

*martinsi* (Koch, 1952: 259) *Phanerotomea* comb. n.

**Type data.** Holotype (Munich Museum) and paratypes (Ditsong Museum, Munich Museum)

*melleus* (Koch, 1952: 248) *Phanerotomea* comb. n.

**Type data.** Holotype (Ditsong Museum) and paratypes (Basel Museum, Ditsong Museum, Munich Museum)

*mendicusestermanni* (Koch, 1952: 259) *Phanerotomea* comb. n.

**Type data.** Holotype (Basel Museum) and paratypes (Basel Museum, Ditsong Museum)

*mendicusmendicus* (Péringuey, 1899: 299) *Psammodes* comb. n.

**Type data.** Syntypes (Cape Museum)

*miles* (Péringuey, 1908: 408) *Psammodes* comb. n.

**Type data.** Holotype (Cape Museum)

*mimeticus* (Koch, 1952: 320) *Phanerotomea* comb. n.

**Type data.** Holotype (Ditsong Museum) and paratypes (Basel Museum, Ditsong Museum, Munich Museum)

*misolampoides* (Fairmaire, 1888b: 258) *Psammodes* comb. n.

**Type data.** Holotype (Leiden Museum)

*mixtus* (Haag-Rutenberg, 1871a: 74, in key) *Psammodes* comb. n.

**Type data.** Syntypes (British Museum)

**Notes.** A detailed morphological description was provided by Haag-Rutenberg (1871b: 73).

*monacha* (Koch, 1952: 267) *Phanerotomea* comb. n.

**Type data.** Holotype (Tervuren Museum) and paratypes (Basel Museum, Ditsong Museum, Tervuren Museum)

*montanus* (Koch, 1952: 283) *Phanerotomea* comb. n.

**Type data.** Holotype and paratype (Tervuren Museum)

*mozambicus* (Koch, 1952: 251) *Phanerotomea* comb. n.

**Type data.** Holotype (Basel Museum)

*muliebriscurtus* (Koch, 1952: 286) *Phanerotomea* comb. n.

**Type data.** Holotype (Cape Museum) and paratypes (Basel Museum, Cape Museum, Ditsong Museum)

*muliebrismuliebris* (Koch, 1952: 285) *Phanerotomea* comb. n.

**Type data.** Holotype (Ditsong Museum) and paratypes (Basel Museum, British Museum, Cape Museum, Ditsong Museum, Munich Museum)

*muliebrissilvestris* (Koch, 1952: 286) *Phanerotomea* comb. n.

**Type data.** Holotype (Ditsong Museum)

*nervosus* (Haag-Rutenberg, 1871a: 74, in key) *Psammodes* comb. n.

**Type data.** Holotype (British Museum – Bates coll.)

**Notes.** A detailed morphological description was provided by Haag-Rutenberg (1871b: 75).

*notatum* (Thunberg, 1787: 48) *Sepidium* comb. n.

**Type data.** Holotype (Uppsala University)

*notaticollis* (Koch, 1952: 263) *Phanerotomea* comb. n.

**Type data.** Holotype (Tervuren Museum) and paratypes (Basel Museum, Ditsong Museum, Tervuren Museum)

*odorans* (Koch, 1952: 321) *Phanerotomea* comb. n.

**Type data.** Holotype (Ditsong Museum) and paratypes (Basel Museum, Budapest Museum, British Museum, California Academy, Ditsong Museum, Munich Museum)

*opacus* (Solier, 1843: 91) *Phanerotomea* comb. n.

**Type data.** Holotype (Warsaw Museum – Dupont collection)

*osbecki* (Billberg, 1815: 281) *Moluris* comb. n.

**Type data.** Lectotype, designated by Ferrer (1991) (Naturhistoriska riksmuseet)

= *Phanerotomasuturalis* Solier, 1843: 92 [syn. by Gebien 1937a: 772]

**Type data.** Holotype (Paris Museum)

*overlaeti* (Koch, 1952: 274) *Phanerotomea* comb. n.

**Type data.** Holotype (Tervuren Museum) and paratype (Basel Museum, Ditsong Museum, Tervuren Museum)

*ovulus* (Haag-Rutenberg, 1871a: 73, in key) *Psammodes* comb. n.

**Type data.** Holotype (Geneva Museum)

**Notes.** A detailed morphological description was provided by Haag-Rutenberg (1871b: 75).

= *Psammodesprobus* Péringuey, 1899: 283 [syn. by Koch (1952: 288)]

**Type data.** Holotype (Cape Museum)

= *Psammodesconsors* Péringuey, 1899: 288 [syn. by Koch (1952: 288)]

**Type data.** Holotype (Cape Museum)

*pachysomaornata* (Koch, 1952: 261) *Phanerotomea* comb. n.

**Type data.** Holotypes (Munich Museum)

*pachysomapachysoma* (Péringuey, 1892: 52) *Psammodes* comb. n.

**Type data.** Syntypes (Cape Museum)

*papillosus* (Koch, 1952: 302) *Phanerotomea* comb. n.

**Type data.** Holotype (Ditsong Museum)

*pedator* (Fairmaire, 1888b: 257), **comb. nov.***Psammodes*

**Type data.** Syntypes (Leiden Museum)

*perlucidus* (Koch, 1952: 246) *Phanerotomea* comb. n.

**Type data.** Holotype (Cape Museum) and paratypes (Basel Museum, Cape Museum, Ditsong Museum)

*planus* (Koch, 1952: 307) *Phanerotomea* comb. n.

**Type data.** Holotype (Tervuren Museum) and paratypes (Basel Museum, Ditsong Museum, Tervuren Museum)

*pretorianus* (Koch, 1952: 279) *Phanerotomea* comb. n.

**Type data.** Holotype (Ditsong Museum)

*procursus* (Péringuey, 1899: 279) *Psammodes* comb. n.

**Type data.** Syntypes (Cape Museum)

*procrustes* (Westwood, 1875: 224) *Moluris* [Westwood, 1875: 224]

**Type data.** Holotype (Oxford University – Westwood coll.)

*protectus* (Koch, 1952: 323) *Phanerotomea* comb. n.

**Type data.** Holotype (Ditsong Museum) and paratypes (Basel Museum, California Academy, Cape Museum, Ditsong Museum, Dundo Museum, Munich Museum)

*punctatissimus* (Koch, 1952: 304) *Phanerotomea* comb. n.

**Type data.** Holotype (Brussels Museum) and paratypes (Basel Museum, Brussels Museum, Ditsong Museum)

*puncticollis* (Koch, 1952: 264) *Phanerotomea* comb. n.

**Type data.** Holotype (Brussels Museum) and paratypes (Basel Museum, Brussels Museum, Ditsong Museum, Tervuren Museum)

*punctipennisplanisculptus* (Koch, 1952: 265) *Phanerotomea* comb. n.

**Type data.** Holotype (Tervuren Museum) and paratypes (Basel Museum, Ditsong Museum, Tervuren Museum)

*punctipennispunctipennis* (Harold, 1878: 106) *Psammodes* comb. n.

**Type data.** Holotype (Munich Museum)

*punctipleura* (Koch, 1952: 306) *Phanerotomea* comb. n.

**Type data.** Holotype (Munich Museum) and paratype (Ditsong Museum)

*rhodesianus* (Koch, 1952: 292) *Phanerotomea* comb. n.

**Type data.** Holotype (Ditsong Museum) and paratype (Basel Museum, Cape Museum)

*roriferus* (Koch, 1952: 253) *Phanerotomea* comb. n.

**Type data.** Holotype (Ditsong Museum) and paratypes (Basel Museum, California Academy, Cape Museum, Ditsong Museum, Munich Museum)

*rowleianus* (Westwood, 1864: 8979) *Moluris* [Westwood, 1864: 8979]

**Type data.** Syntypes (British Museum)

= *Psammodeszoutpansbergianus* Péringuey, 1904: 231 [syn. Gebien 1937a: 768]

**Type data.** Holotype (Cape Museum)

*rufipes* (Harold, 1878: 106) *Psammodes* comb. n.

**Type data.** Syntypes (Munich Museum)

= *Psammodescongoanus* Gebien, 1920b: 7 [syn. by Koch 1952: 262]

**Type data.** Syntypes (Munich Museum)

*saltuarius* (Koch, 1952: 250) *Phanerotomea* comb. n.

**Type data.** Holotype (Ditsong Museum)

*scabricollis* (Gerstaecker, 1854: 532) *Phanerotomea* comb. n.

**Type data.** Holotype (Berlin Museum)

*scopulipes* (Koch, 1952: 319) *Phanerotomea* comb. n.

**Type data.** Holotype (Ditsong Museum) and paratypes (Ditsong Museum, Munich Museum)

*scrobicollisgriqua* (Koch, 1952: 234) *Phanerotomea* comb. n.

**Type data.** Holotype (Ditsong Museum) and paratypes (Cape Museum, Ditsong Museum)

*scrobicollisscrobicollis* Fåhraeus, 1870: 270

**Type data.** Holotype (Naturhistoriska riksmuseet) and paratypes (Naturhistoriska riksmuseet, Warsaw Museum)

*scrobicollissimulans* (Koch, 1952: 235) *Phanerotomea* comb. n.

**Type data.** Holotype (Ditsong Museum) and paratypes (Agricultural Institute, Basel Museum, Cape Museum, Ditsong Museum, Durban Museum, Munchen Museum, Pretoria University)

*semirasus* (Koch, 1952: 296) *Phanerotomea* comb. n.

**Type data.** Holotype (Tervuren Museum) and paratypes (Basel Museum, Ditsong Museum, Tervuren Museum)

*semiscabrum* (Haag-Rutenberg, 1871a: 73, in key) *Psammodes* comb. n.

**Type data.** Holotype (Naturhistoriska riksmuseet)

**Notes.** Type deposition information after Koch (1952). A detailed morphological description was provided by Haag-Rutenberg (1871b: 76).

= *Psammodessperabilis* Peinguey, 1899: 289 [syn. by Koch (1952: 240)]

**Type data.** Holotypes (Cape Museum)

*sericicollis* (Koch, 1952: 254) *Phanerotomea* comb. n.

**Type data.** Holotype (Ditsong Museum) and paratypes (Basel Museum, Ditsong Museum, Munich Museum)

*similis* (Péringuey, 1899: 291) *Psammodes* comb. n.

**Type data.** Syntypes (Cape Museum)

*sjoestedti* (Gebien, 1910b: 372) *Psammodes* comb. n.

**Type data.** Holotype (Munich Museum)

*spatulipes* (Koch, 1952: 318) *Phanerotomea* comb. n.

**Type data.** Holotype (Ditsong Museum)

*specularis* (Péringuey, 1899: 286) *Psammodes* comb. n.

**Type data.** Syntypes (Cape Museum, Munich Museum)

*spinigerus* (Koch, 1952: 269) *Phanerotomea* comb. n.

**Type data.** Holotype (California Academy) and paratypes (Basel Museum, California Academy, Ditsong Museum)

*stevensoni* (Koch, 1952: 284) *Phanerotomea* comb. n.

**Type data.** Holotype (Ditsong Museum) and paratype (Basel Museum, Ditsong Museum)

*tarsocnoides* (Koch, 1952: 267) *Phanerotomea* comb. n.

**Type data.** Holotype (Basel Museum) and paratypes (Basel Museum, Ditsong Museum)

*temulentus* (Koch, 1952: 284) *Phanerotomea* comb. n.

**Type data.** Holotype (Basel Museum) and paratype (Basel Museum, Cape Museum)

*tenebrosusmelanarius* (Haag-Rutenberg, 1871a: 71, in key) *Psammodes* comb. n.

**Type data.** Syntypes (Geneva Museum)

**Notes.** A detailed morphological description was provided by Haag-Rutenberg (1871b: 64).

*tenebrosustenebrosus* (Erichson, 1843: 242) *Moluris* comb. n.

**Type data.** Syntypes (Humboldt University)

*tibialis* (Haag-Rutenberg, 1871a: 63, in key) *Psammodes* comb. n.

**Type data.** Holotype (Munich Museum – Haag-Rutenberg coll.)

**Notes.** A detailed morphological description was provided by Haag-Rutenberg (1871b: 44).

*torosus* (Koch, 1952: 235) *Phanerotomea* comb. n.

**Type data.** Holotype (Ditsong Museum) and paratypes (Agricultural Institute, Basel Museum, Cape Museum, Ditsong Museum, Munich Museum, Pretoria University)

*transversicollis* (Haag-Rutenberg, 1879: 291) *Psammodes* comb. n.

**Type data.** Syntypes (British Museum, Munich Museum – Haag-Rutenberg coll.)

= *Psammodescinctipennis* Fairmaire, 1899: 180 [syn. with *P.valens* by Péringuey 1904: 297]

**Type data.** Syntypes (Paris Museum)

= *Psammodesvalens* Péringuey, 1899: 276 [syn. by Koch (1952: 281)]

**Type data.** Syntypes (Cape Museum)

*tumidus* (Haag-Rutenberg, 1871a: 73, in key) *Psammodes* comb. n.

**Type data.** Holotype (Munich Museum – Haag-Rutenberg coll.)

**Notes.** A detailed morphological description was provided by Haag-Rutenberg (1871b: 72).

*umvumanus* (Koch, 1952: 280) *Phanerotomea* comb. n.

**Type data.** Holotype (Cape Museum)

*vagus* (Péringuey, 1899: 288) *Psammodes* comb. n.

**Type data.** Syntypes (Cape Museum)

*vaticinus* (Péringuey, 1899: 279) *Psammodes* comb. n.

**Type data.** Holotype (Cape Museum)

*verecundus* (Péringuey, 1899: 286) *Psammodes* comb. n.

**Type data.** Syntypes (Cape Museum)

*vetustus* (Koch, 1952: 263) *Phanerotomea* comb. n.

**Type data.** Holotype (Tervuren Museum) and paratypes (Basel Museum, Ditsong Museum, Tervuren Museum)

*vexator* (Péringuey, 1899: 287) *Psammodes* comb. n.

**Type data.** Holotype (Cape Museum)

*virago* (Koch, 1952: 236) *Phanerotomea* comb. n.

**Type data.** Holotype (Ditsong Museum) and paratypes (Agricultural Institute, Basel Museum, California Academy of Sciences, Cape Museum, Cornell University, Ditsong Museum, Munich Museum, Pretoria University, Rhodesia Museum)

*warmeloi* (Koch, 1953b: 86) *Phanerotomea* comb. n.

**Type data.** Holotype (Cape Museum) and paratypes (Basel Museum, Cape Museum, Ditsong Museum)

*zanzibaricus* (Haag-Rutenberg, 1875: 78) *Psammodes* comb. n.

**Type data.** Holotype (Cape Museum)

##### Genus *Psammoryssus* Kolbe, 1886: 289

**Type species.***Psammoryssustitanus* Kolbe, 1886 (by monotypy)

*titanus* Kolbe, 1886: 290

**Type data.** Syntypes (Berlin Museum)

##### Genus *Stridulomus* Koch, 1955: 37

**Type species.***Psammodessulcicollis* Péringuey, 1885 (by monotypy)

*sulcicollis* (Péringuey, 1885: 110) *Psammodes* [Koch, 1955: 37]

**Type data.** Syntypes (Cape Museum)

= *Psammodesrehbocki* Kolbe, 1904: 299 [syn. by Péringuey (1908: 395)]

**Type data.** Syntypes (Berlin Museum)

##### Genus *Tarsocnodes* Gebien, 1920: 82

**Type species.***Psammodesmolossa* Haag-Rutenberg, 1871 (by original designation)

*albarenarum* Penrith, 1987: 252

**Type data.** Holotype (Bloemfontein Museum) and paratypes (British Museum, Ditsong Museum, Pretoria University)

*aquamontis* Penrith, 1987: 249

**Type data.** Holotype (Ditsong Museum) and paratypes (British Museum, Ditsong Museum, Windhoek Museum)

*brendelli* Penrith, 1987: 240

**Type data.** Holotype (British Museum) and paratypes (British Museum, Ditsong Museum)

*albarenarumcompressitarsis* (Müller, 1887: 299) *Psammodes* [Gebien, 1920: 83]

**Type data.** Lectotype, designated by Penrith (1986: 258) (Leiden Museum, Munich Museum)

*albarenarumdilaticollis* (Muller, 1887: 298) *Psammodes* [Gebien, 1937a: 759]

**Type data.** Lectotype, designated by Penrith (1986: 241) (Leiden Museum, Munich Museum)

*ephialtes* Koch, 1952: 334

**Type data.** Holotype (Ditsong Museum) and paratypes (Basel Museum, British Museum, Cape Museum, Ditsong Museum, Munich Museum)

*errans* (Péringuey, 1892: 53) *Psammodes* [Gebien, 1920: 83]

**Type data.** Syntypes (Cape Museum)

**Notes.** In his original description, Péringuey (1892) did not indicate a collection locality, and Penrith (1987) noted that the holotype did not have locality labels. However, in the original description, Péringuey gives a size range, implying multiple specimens, and Koch (1952) specified a specimen from “Nordliches Ovamboland” as the type.

= *Tarsocnodesspectabilis* Gebien, 1920: 84 [syn. by Penrith (1987: 258)]

**Type data.** Syntypes (Hamburg University)

**Notes.** Type deposition information after Penrith (1987).

*finitima* Koch, 1952: 331

**Type data.** Holotype (Ditsong Museum)

*gracilipes* Koch, 1952: 332

**Type data.** Holotype (Ditsong Museum)

**Notes.** Type deposition information after Penrith (1987).

*granulicauda* Penrith, 1987: 242

**Type data.** Holotype (Ditsong Museum) and Paratypes (British Museum, Ditsong Museum, Windhoek Museum)

*iflundi* Koch, 1952: 335

**Type data.** Holotype (Ditsong Museum) and paratypes (Basel Museum, Cape Museum, Ditsong Museum, California Academy)

*ephialteslaevipennis* (Haag-Rutenberg, 1879: 291) *Psammodes* [Koch, 1962d: 334]

**Type data.** Lectotype, designated by Penrith (1986: 239) (British Museum) and paralectotype (Munich Museum)

*madida* Koch, 1952: 326

**Type data.** Holotype (Ditsong Museum)

*michaelis* Penrith, 1986: 247

**Type data.** Holotype (Windhoek Museum) and parartypes (Ditsong Museum, Windhoek Museum)

*molossa* (Haag-Rutenberg, 1871a: 83) *Psammodes* [Gebien, 1920: 83]

**Type data.** Holotype (Munich Museum – Haag-Rutenberg coll.)

= Moluris (Phanerotoma) gravida (Westwood, 1875: 223) [syn. by. Haag-Rutenberg (1879: 290)]

**Type data.** Syntypes (British Museum)

*monasterialis* Koch, 1952: 329

**Type data.** Holotype (Tervuren Museum) and paratypes (Basel Museum, Companhia Diamantes, Ditsong Museum, Dundo Museum)

= *Tarsocnodesvariabilisdissoluta* Koch, 1952: 330 [syn. by Penrith (1987: 246)]

**Type data.** Holotype (Tervuren Museums) and paratypes (Ditsong Museum)

= *Tarsocnodesvariabilisvariabilis* Koch, 1952: 330 [syn. by Penrith (1987: 246)]

**Type data.** Holotype (Tervuren Museum) and paratypes (Basel Museum, Ditsong Museum, Munich Museum, Tervuren Museum)

*nielseni* Ferrer, Evanno & Evanno, 2010: 195

**Type data.** Holotype (Naturhistoriska riksmuseet) and paratype (Ferrer collection)

*praegrandishorribillis* Koch, 1952: 328

**Type data.** Holotype (Ditsong Museum)

*praegrandispraegrandis* Koch, 1952: 327

**Type data.** Holotype (Tervuren Museum) and paratypes (Basel Museum, Brussels Museum, Companhia Diamantes, Dundo Museum, Munich Museum, Tervuren Museum)

*prozeskyorum* Penrith, 1986: 254

**Type data.** Holotype (Ditsong Museum) and paratypes (British Museum, Cape Museum, Ditsong Museum)

*rugicollis* Gebien, 1920: 85

**Type data.** Holotype and paratypes (Munich Museum)

**Notes.** Type deposition information after Koch (1952).

*tarsalis* (Haag-Rutenberg, 1871a: 52) *Psammodes* [Gebien, 1920: 83]

**Type data.** Holotype (Naturhistoriska riksmuseet)

**Notes.** Type deposition information after Penrith (1986).

*variolata* Koch, 1952: 329

**Type data.** Holotype (Tervuren Museum)

*vernayi* Koch, 1952: 250

**Type data.** Holotype (Ditsong Museum)

*whiteheadi* Penrith, 1986: 239

**Type data.** Holotype and paratypes (Windhoek Museum)

#### Subtribe Sepidiina Eschscholtz, 1829: 4

**Type genus.***Sepidium* Fabricius, 1775

**Taxonomic diversity.** (8 gen., 124 spp.): *Dimoniacis* (3 ssp.), *Echinotus* (2), *Peringueyia* (1), *Sepidiopsis* (3), *Sepidiostenus* (7), *Sepidium* (51), *Vieta* (52), *Vietomorpha* (5).

**Distribution.** Widely distributed throughout the Mediterranean area and Sub-Saharan Africa, except its western part. Majority of the species were described form from the Horn of Africa. *Vieta* representatives are the only species, which were described from the area south from equator, while only *Sepidium* species have loci tipici north from Tropic of Cancer (Fig. 51). It needs to be noted that the distributional image presented here is probably biased due to adopted methodological approach (i.e., illustration of loci typici). For many of the analysed species the original distributional information is very general (country records) and was not georeferenced here.

##### Genus *Dimoniacis* Koch, 1958: 44

**Type species.***Dimoniacisjacksoni* Koch, 1958 (by original designation)

*jacksoni* Koch, 1958: 44

**Type data.** Holotype (Ditsong Museum)

*lavranosi* Ardoin, 1979: 60

**Type data.** Holotype and paratypes (Paris Museum)

*puccionii* Ferrer, 1995: 27

**Type data.** Holotype (Florence Museum)

##### Genus *Echinotus* Solier, 1843: 30

**Type species.***Sepidiumspinicollis* Laporte, 1840 (by original designation)

**Notes.** During the compilation of this catalog, a junior homonym of *Echinotus* Solier, 1843 was found: *Echinotus* Marwick, 1935: 301 (**Type species.***Aviculaechinata* Smith, 1817; Mollusca: Pteriidae). *Ulamus* Kaminski, **nom. nov.** is introduced here as a replacement name for the above-mentioned pteriid genus. This newly introduced name honours Stanisław Marcin Ulam, Polish-American scientist, inventor of the Monte Carlo method of computation.

*natalensis* Chevrolat, 1874: 331

**Type data.** Holotype (Paris Museum)

*spinicollis* (Laporte, 1840: 197) *Sepidium* [Solier, 1843: 31]

**Type data.** Syntypes (Paris Museum)

##### Genus *Peringueyia* Koch, 1958: 44

**Type species.***Echinotusdispar* Péringuey, 1899 (by monotypy)

*dispar* (Péringuey, 1899: 302) *Echinotus* [Koch, 1958: 44]

**Type data.** Holotype (Cape Museum)

##### Genus *Sepidiopsis* Gestro, 1892: 771

**Type species.***Sepidiopsiscornigera* Gestro, 1892 (by original designation)

*ardoini* Ferrer, 1995: 26

**Type data.** Holotype (Florence Museum)

*cornigera* Gestro, 1892: 772

**Type data.** Holotype (Genoa Museum)

*villosa* Gestro, 1892: 773

**Type data.** Holotype (Genoa Museum)

##### Genus *Sepidiostenus* Fairmaire, 1884: LXXV

**Type species.***Sepidiostenuserinaceus* Fairmaire, 1884 (by monotypy)

= *Sepidiacis* Fairmaire, 1884: CXLVI [junior subjective synonym proposed by Gestro (1892: 775)]

**Type species.***Sepidiaciscompressa* Fairmaire, 1884 (subsequent designation by Kirby (1885: 83))

**Notes.** This taxon was redescribed as new by Fairmaire (1887: 185).

*compressus* (Fairmaire, 1884: CXLVI) *Sepidiacis*

**Type data.** Holotype (Paris Museum)

*dolichopus* Gestro, 1898: 517

**Type data.** Holotype (Genoa Museum)

*erinaceus* Fairmaire, 1887: 185

**Type data.** Holotype (Paris Museum)

*fairmairei* Gestro, 1898: 512

**Type data.** Holotype (Genoa Museum)

*longipennis* Gestro, 1898: 516

**Type data.** Syntypes (Genoa Museum)

*pradieri* (Guérin-Méneville, 1858: 128) *Sepidium* [Gestro, 1892: 776]

**Type data.** Syntypes (Paris Museum)

*ruspolii* Gestro, 1898: 514

**Type data.** Holotype (Genoa Museum)

##### Genus *Sepidium* Fabricius, 1775: 250

**Type species.***Sepidiumtricuspidatum* Fabricius, 1775 (by subsequent designation by Latreille (1810: 429))

= *Espidium* Rafinesque, 1815: 113

**Notes.** Unnecessary replacement name for *Sepidium* Fabricius, 1775

*aitagiae* Escalera, 1913: 41

**Type data.** Holotype (Madrid Museum)

*aliferum* Erichson, 1841: 178

**Type data.** Holotype (British Museum)

= *Sepidiumdouei* Solier, 1843: 18 [syn. by Erichson (1844: 343)]

**Type data.** Syntypes (Paris Museum)

*aper* Fairmaire, 1884: LXXV

**Type data.** Holotype (Paris Museum)

*barbarum* Solier, 1843: 23

**Type data.** Syntypes (Warsaw Museum – Dupont collection)

= *Sepidiumservillei* Solier, 1843: 24 [syn. by Reitter (1914: 384)]

**Type data.** Holotype (Paris Museum)

= *Sepidiumpallens* Allard, 1874: 137 [syn. by Reitter (1914: 384)]

**Type data.** Holotype (Paris Museum)

= *Sepidiumbarbarumsolieri* Desbrochers des Loges, 1881: 101 [syn. by Reitter (1914: 384)]

**Type data.** Syntypes (Paris Museum)

*bicaudatum* Fairmaire, 1871: 388

**Type data.** Holotype (Paris Museum)

*bidentatum* Solier, 1843: 15

**Type data.** Syntypes (Paris Museum)

*bilobatum* Gahan, 1900: 30

**Type data.** Holotype (British Museum)

*boranum* Mal, 1986b: 151

**Type data.** Holotype (Trieste Museum) and paratypes (Berlin Museum, British Museum, Budapest Museum, Tervuren Museum, Trieste Museum)

*bulbiferum* Gerstaecker, 1884: 55

**Type data.** Syntypes (Berlin Museum)

*brevicaudatum* Fairmaire, 1882b: LI

**Type data.** Holotype (Paris Museum)

*capricorne* Desbrochers des Loges, 1881: 96

**Type data.** Holotype (Paris Museum)

**Notes.** Species concept after Mal (1984).

*crassicaudatum* Gestro, 1878: 320

**Type data.** Holotype (Genoa Museum)

*cristatum* Fabricius, 1775: 250

**Type data.** Syntypes (Copenhagen Museum, Kiel Museum)

= *Tenebrionotoceros* Pallas, 1781: 59 [syn. by Allard (1874: 143)]

**Type data.** Syntypes (Humboldt University – Pallas collection)

*cylindrigerum* Fairmaire, 1882a: 75

**Type data.** Syntypes (Paris Museum)

*cyrenaicum* Schuster, 1928: 122

**Type data.** Syntypes (Basel Museum)

*dathan* Crotch, 1872: 268

**Type data.** Holotype (Cambridge Museum)

= *Sepidiumabiram* Crotch, 1872: 268 [syn. by Gebien (1937a: 781)]

**Type data.** Holotype (Cambridge Museum)

= *Sepidiumvietaeformis* Reitter, 1914: 385 [syn. by Gebien (1937a: 781)]

**Type data.** Syntypes (Basel Museum, Budapest Museum, Vienna Museum)

*fusiforme* Kwieton, 1980: 17

**Type data.** Holotype and paratype (Budapest Museum)

*gypsicola* Escalera, 1913: 42

**Type data.** Syntypes (Madrid Museum)

*hoseini* Escalera, 1911: 301

**Type data.** Holotype (Madrid Museum)

*hystryxdesertica* Espanol, 1944: 12

**Type data.** Holotype (Barcelona Museum)

*hystryxhystryx* Antoine, 1932: 185

**Type data.** Holotype (Paris Museum)

*hystryxifniensis* Escalera, 1940: 5

**Type data.** Syntypes (Madrid Museum)

**Notes.**Escalera (1940) designated a variety “*subdesertica*”, expressly giving it the infrasubspecific rank. Therefore, according to the Art. 45.6.4. of the ICZN (1999) is should not be treated as a subspecies.

*inaequale* Reitter, 1914: 386

**Type data.** Syntyptes (Budapest Museum)

*kaszabi* Mal, 1990: 64

**Type data.** Holotype and paratype (British Museum)

*kelleri* Fairmaire, 1893: 151

**Type data.** Holotype (Paris Museum)

*lusitanicum* Kaszab & Pinheiro, 1972

**Type data.** Holotype (Budapest Museum) and paratype (Madrid Museum)

*magnum* Gahan, 1900: 29

**Type data.** Holotype and paratypes (British Museum)

*mali* Ferrer & Martínez, 2012, replacement name

= *Sepidiumelongatum* Mal, 1984: 200 [junior primary homonym of *Sepidiumelongatus* Olivier, 1795: 8]

**Type data.** Holotype (Brussels Museum) and paratypes (British Museum, Brussels Museum, Julio Ferrer collection)

*marraquense* Escalera, 1911: 302

**Type data.** Syntypes (Basel Museum, Madrid Museum)

*mesopotamicum* Reitter, 1914: 386

**Type data.** Syntypes (Basel Museum, Budapest Museum, Vienna Museum)

*mskalicum* Escalera, 1914: 307

**Type data.** Syntypes (Madrid Museum)

*obtusangulum* Fairmaire, 1882a: 73

**Type data.** Holotype (Paris Museum)

*pagesii* Fairmaire, 1894: 321

**Type data.** Syntypes (Basel Museum, British Museum, Budapest Museum)

*penicilligerum* Karsch, 1881: 49

**Type data.** Syntypes (Berlin Museum)

*perforatum* Allard, 1874: 130

**Type data.** Holotype (Paris Museum)

**Notes.** Species concept after Mal (1984).

*peyerimhoffi* Antoine, 1932: 183

**Type data.** Syntypes (Basel Museum, Budapest Museum, Paris Museum)

*reichei* Allard, 1870: 49

**Type data.** Syntypes (Paris Museum)

= *Sepidiumreicheibispinicollis* Reitter, 1914: 389 [syn. by Kwieton 1980: 6]

**Type data.** Syntypes (Budapest Museum)

*requieni* Solier, 1843: 29

**Type data.** Holotype (Paris Museum)

*ruspoliiruspolii* Fairmaire, 1893: 150

**Type data.** Holotype (Paris Museum) and paratype (Basel Museum)

*ruspoliispectabile* Kulzer, 1960: 309

**Type data.** Holotype (Berlin Museum) and paratypes (Basel Museum)

*scebelianum* Mal, 1986b: 153

**Type data.** Holotype (Trieste Museum) and paratypes (Berlin Museum, British Museum, Budapest Museum, Tervuren Museum, Trieste Museum)

*siculum* Solier, 1843: 19

**Type data.** Syntypes (Paris Museum)

= *Sepidiumgenei* Solier, 1843: 20 [syn. by Reitter 1914: 384]

**Type data.** Syntypes (Paris Museum)

*tricuspidatumkorah* Crotch, 1872: 268

**Type data.** Holotype (Cambridge Museum)

*tricuspidatummogadoricum* Escalera, 1914: 306

**Type data.** Syntypes (Madrid Museum)

= *Sepidiumimmundum* Reitter, 1914: 388 [syn. by Kwieton 1980: 10]

**Type data.** Syntypes (Basel Museum, Budapest Museum)

= *Sepidiummogadoricumguisseri* Kocher, 1958: 111 [syn. by Kwieton 1980: 10]

**Type data.** Syntypes (Rabat Institute)

= *Sepidiummogadoricumschrammi* Kocher, 1958: 110 [syn. by Kwieton 1980: 10]

**Type data.** Syntypes (Rabat Institute)

*tricuspidatummultispinosum* Solier, 1843: 26

**Type data.** Holotype (Marseille Museum)

= *Sepidiumlaghoatense* Baudi, 1875: 695 [syn. by Kwieton 1980: 11]

**Type data.** Syntypes (Basel Museum)

*tricuspidatumtomentosum* Erichson, 1841: 179

**Type data.** Syntypes (Humboldt University)

= *Sepidiumbarthelemyi* Solier, 1843: 24 [syn. by Reitter (1914: 387)]

**Type data.** Syntypes (Marseille Museum)

= *Sepidiummaillei* Solier, 1843: 27 [syn. by Reitter (1914: 387)]

**Type data.** Syntypes (Marseille Museum)

= *Sepidiumserratum* Solier, 1843: 28 [syn. by Kwieton 1980: 11]

**Type data.** Holotype (Paris Museum)

= *Sepidiumserratumremotum* Sahlberg, 1903: 50 [syn. By Reitter (1914: 387)]

**Type data.** Syntypes (Helsinki University)

*tricuspidatumtricuspidatum* Fabricius, 1775: 250

**Type data.** Syntypes (Copenhagen Museum, Kiel Museum)

= *Tenebrioalexandrinus* Forskål, 1775: 80 [Gmelin 1790: 2008]

**Type data.** Syntypes (Copenhagen Museum)

= *Sepidiumflexuosum* Solier, 1843: 25 [syn. by Reitter (1914: 386)]

**Type data.** Holotype (Paris Museum)

= *Sepidiumtricuspidatumcerisyi* Solier, 1843: 26 [syn. by Reitter (1914: 386)]

**Type data.** Syntypes (Marseille Museum)

**Notes.** Originally described as a variety of the nominotypical form.

*tualensis* Escalera, 1940: 9

**Type data.** Syntypes (Madrid Museum)

*uncinatum* Erichson, 1841: 178

**Type data.** Holotype (Humboldt University)

= *Sepidiummittrei* Solier, 1843: 16 [syn. by Reitter (1914: 385)]

**Type data.** Syntypes (Paris Museum)

= *Sepidiummittreibicorne* Solier, 1843: 17 [syn. by Reitter (1914: 385)]

**Type data.** Syntypes (Paris Museum)

**Notes.** Originally described as a variety of the nominotypical form.

= *Sepidiumsubcornutum* Escalera, 1925: 376 [syn. by Kwieton 1980: 7]

**Type data.** Syntypes (Madrid Museum)

= *Sepidiumsefrense* Rotrou, 1943: 235 [syn. by Kwieton 1980: 7]

**Type data.** Holotype (Paris Museum)

*variegatum* (Fabricius, 1792: 112) *Tenebrio* [Solier 1843: 21]

**Type data.** Syntypes (British Museum, Kiel Museum)

= *Sepidiumvariegatumangustatum* Solier, 1843: 21 [syn. by Reitter (1916: 383)]

**Type data.** Syntypes (Paris Museum)

= *Sepidiumdufouri* Solier, 1843: 21 [syn. by Reitter (1914: 383)]

**Type data.** Holotype (Paris Museum)

= *Sepidiumlaterale* Allard, 1874: 133 [syn. by Reitter (1914: 383)]

**Type data.** Syntypes (Paris Museum)

= *Sepidiumvariegatumdispar* Desbrochers des Loges, 1881: 101 [syn. by Reitter (1916: 383)]

**Type data.** Syntypes (Paris Museum)

= *Sepidiumvariegatumintegrum* Desbrochers des Loges, 1881: 100 [syn. by Reitter (1914: 383)]

**Type data.** Syntypes (Paris Museum)

= *Sepidiumvariegatumsubfurcatum* Desbrochers des Loges, 1881: 100 [syn. by Gebien (1910b: 170)]

**Type data.** Syntypes (Paris Museum)

*wagneri* Erichson, 1841: 179

**Type data.** Syntypes (Humboldt University)

= *Sepidiumwagnericonfusum* Allard, 1874: 140 [syn. by Reitter (1914: 388)]

**Type data.** Syntypes (Paris Museum)

= *Sepidiumwagnerimacrotys* Antoine, 1951a: 94 [syn. by Kwieton 1980: 13]

**Type data.** Syntypes (Paris Museum)

##### Genus *Vieta* Laporte, 1840: 196

**Type species.***Sepidiumvestitum* Guérin-Méneville, 1831 (designated by Hope (1841: 116))

= *Dymonus* Solier, 1843: 7 [junior subjective synonym proposed by Lesne (1922: 696)]

**Type species.***Sepidiumvestitum* Guérin-Méneville, 1831 (by original designation)

= *Divieta* Reitter, 1914: 390 [junior subjective synonym proposed by Lesne (1922: 696)]

**Type species.***Vietacostata* Allard, 1874 (by subsequent designation by Löbl and Smetana (2008: 40))

*algeriana* Allard, 1870: 50

**Type data.** Syntypes (Paris Museum)

*angolensisangolensis* (Quedenfeldt, 1885: 7) *Sepidium* [Koch, 1958: 53]

**Type data.** Syntypes (Humboldt University, Paris Museum)

*angolensisbiena* Koch, 1958: 53

**Type data.** Holotype and paratypes (California Academy)

*angolensiseduardi* Koch, 1958: 54

**Type data.** Holotype and paratypes (California Academy)

*angolensistransversa* (Fairmaire, 1888b: 262) *Sepidium* [Koch, 1958: 53]

**Type data.** Holotype (Leiden Museum)

= *Sepidiumovampoense* Péringuey, 1892: 55 [syn. by Koch (1958: 53)]

**Type data.** Holotype (Cape Museum)

*angustula* Lesne, 1922: 697

**Type data.** Holotype (Paris Museum)

*aper* Fairmaire, 1887: 278

**Type data.** Holotype (Paris Museum)

*apicorne* (Fairemaire, 1882: 75) *Sepidium* [Koch, 1958: 42]

**Type data.** Holotype (Paris Museum)

*borana* Gridelli, 1939a: 114

**Type data.** Syntypes (Trieste Museum)

*bulbifera* Fairmaire, 1897: 116

**Type data.** Holotype (Paris Museum)

*clypeata* Gahan, 1896: 454

**Type data.** Holotype (British Museum)

*cornutipennis* Gebien, 1937b: 37

**Type data.** Holotype (Trieste Museum) and paratype (Basel Museum, Trieste Museum)

*costata* Allard, 1874: 149

**Type data.** Holotype (Basel Museum)

*crinita* Allard, 1882: LXXXVII

**Type data.** Syntypes (Paris Museum)

= *Sepidiumzambezianum* Péringuey, 1892: 123 [syn. by Koch (1958: 52)]

**Type data.** Syntypes (British Museum and Paris Museum)

*deckerti* Ferrer, 2004a: 217

**Type data.** Holotype (Berlin Museum)

*erecticollis* Ancey, 1881b: 461

**Type data.** Syntypes (Paris Museum)

*erosa* Allard, 1882: LXXXVII

**Type data.** Holotype (Basel Museum)

*furcifera* (Gerstaecker, 1884: 55) *Sepidium* [Gebien, 1937b: 56]

**Type data.** Syntypes (Berlin Museum)

*gracilenta* Ancey, 1881a: 397

**Type data.** Syntypes (Paris Museum)

*grisea* Gridelli, 1939a: 421

**Type data.** Syntypes (Trieste Museum)

*grixonii* Gestro, 1895: 375

**Type data.** Holotype (Genoa Museum)

*hamaticollis* (Fairmaire, 1887: 277) *Sepidium* [Koch, 1958: 43]

**Type data.** Holotype (Paris Museum)

*holdhausi* Reitter, 1914: 391

**Type data.** Holotype (Vienna Museum)

*lacunosa* Fairmaire, 1894: CCLII

**Type data.** Holotype (Basel Museum)

*longehirta* (Fairmaire, 1887: 277) *Sepidium* [Koch, 1958: 43]

**Type data.** Holotype (Paris Museum)

*longepilosa* Fairmaire, 1891b: CCXCIV

**Type data.** Holotype (Paris Museum)

*luctuosa* Fairmaire, 1894: 392

**Type data.** Holotype (Paris Museum)

*lutulenta* Gestro, 1895: 143

**Type data.** Holotype (Genoa Museum)

*luxorii* Allard, 1874: 150

**Type data.** Holotype (Paris Museum)

*millingenii* Kirchsberg, 1877: 203

**Type data.** Syntypes (Basel Museum)

*montana* Fairmaire, 1894: 392

**Type data.** Holotype (Paris Museum)

*muscosa* (Gerstaecker, 1871: 60) *Sepidium* [Koch, 1958: 43]

**Type data.** Syntypes (Berlin Museum)

*ovalis* Allard, 1874: 149

**Type data.** Syntypes (Basel Museum, British Museum)

*pallidicornis* Koch, 1958: 219

**Type data.** Holotype (Cape Museum) and paratypes (Cape Museum, Ditsong Museum)

*protensa* Fairmaire, 1891b: CCXCV

**Type data.** Syntypes (Paris Museum)

*punctipennis* Reitter, 1914: 390

**Type data.** Holotype (Vienna Museum)

*ramosipilus* Koch, 1958: 219

**Type data.** Syntypes (Cape Museum, Ditsong Museum)

*rendiliana* Lesne, 1922: 696

**Type data.** Holotype (Paris Museum)

*robusta* Lesne, 1922: 697

**Type data.** Holotype (Paris Museum)

*russoi* Gebien, 1937b: 38

**Type data.** Holotype (Trieste Museum) and paratype (Basel Museum, Museo Civico Filangieri)

*senegalensisdongolensis* Laporte, 1840: 197

**Type data.** Holotype (Paris Museum)

**Notes.** Taxonomic concept after Gebien (1937b: 56)

= *Dymonusdufossei* Solier, 1843: 10 [syn. by Baudi (1876: 32)]

**Type data.** Holotype (Paris Museum)

*senegalensissenegalensis* (Klug, 1835: 40)

**Type data.** Syntypes (Humboldt University)

= *Sepidiumvestitum* Guérin-Méneville, 1831: 114 [syn. by Allard (1874: 145)]

**Type data.** Syntypes (Paris Museum)

*sexcornuta* (Fairmaire, 1897: 115) *Sepidium* [Koch, 1958: 42]

**Type data.** Holotype (Paris Museum)

*speculifera* Gebien, 1910a: 158

**Type data.** Syntypes (Basel Museum, Tervuren Museum)

*spiculosa* (Gerstaecker, 1884: 55) *Sepidium* [Gebien, 1937b: 56]

**Type data.** Holotype (Berlin Museum)

*subcaudata* Lesne, 1922: 696

**Type data.** Holotype (Paris Museum)

*tuberculata* (Solier, 1844: 11) *Dymonus* [Koch, 1958: 43]

**Type data.** Holotype (Torino Museum – Spinola coll.)

= *Dymonusgibbicollis* Solier, 1843: 12 [syn. by Reitter 1914: 390]

**Type data.** Holotype (Warsaw Museum – Dupont collection)

*tuberosa* Fairmaire, 1882a: 76

**Type data.** Holotype (Paris Museum)

*uncigera* Ancey, 1881b: 461

**Type data.** Holotype (Paris Museum)

*vietomorphoides* Kwieton, 1978: 11

**Type data.** Holotype (Prague Museum)

*villosula* (Fairmaire, 1882a: 74) *Sepidium* [Koch, 1958: 42]

**Type data.** Holotype (Paris Museum)

*zavatartii* Gridelli, 1939a: 110

**Type data.** Syntypes (Trieste Museum)

##### Genus *Vietomorpha* Fairmaire, 1887: 186

**Type species.***Vietomorphafoveipennis* Fairmaire, 1887 (by monotypy)

*abyssinica* Mal, 1986a: 17

**Type data.** Holotype (Brussels Museum) and paratypes (British Museum, Budapest Museum, Ditsong Museum, Ohio State, Tervuren Museum, Paris Museum)

*bartolozzii* Mal, 1986a: 19

**Type data.** Holotype and paratype (Florence Museum)

*crassipes* Mal, 1986a: 16

**Type data.** Holotype (Ditsong Museum) and paratypes (British Museum, Budapest Museum, Ditsong Museum, Paris Museum)

*foveipennis* Fairmaire, 1887: 186

**Type data.** Holotype (Basel Museum)

= *Vietomorphaarabica* Schuster & Gebien, 1938: 59 [syn. by Gridelli (1953: 44)]

**Type data.** Syntypes (Basel Museum)

*tuberosa* (Fairmaire, 1882a: 76) Vieta [Mal, 1986a: 21]

**Type data.** Holotype (Paris Museum)

= *Vietasenegalensissomalica* Gebien, 1937b: 41 [syn. by Mal (1986a: 21)]

**Type data.** Syntypes (Genoa Museum)

#### Subtribe Trachynotina Koch, 1955: 34

**Type genus.***Trachynotus* Latreille, 1828

**Taxonomic diversity.** (10 gen., 218 spp.): *Cyrtoderes* (5 ssp.), *Epairopsis* (4), *Ethmus* (30), *Histrionotus* (2), *Microphligra* (1), *Ossiporis* (11), *Oxycerus* (1), *Somaticus* (148), *Trachynotus* (15), *Trichethmus* (1).

**Distribution.** Widely distributed in the southern part of the Afrotropical Realm (south from equator) (Fig. 51).

##### Genus *Cyrtoderes* Dejean, 1834: 181

**Type species.***Sepidiumlacunosum* Thunberg, 1784 (by subsequent designation by Bousquet & Bouchard (2013: 45); syn of *Tenebriocristatus* DeGeer, 1778)

= *Phligra* Laporte, 1840: 197 [junior objective synonym; see Bousquet & Bouchard (2013: 45)]

**Type species.***Phligradegeeri* Laporte, 1840 (by monotypy); syn of *Tenebriocristatus* DeGeer, 1778.

*cristatuscristatus* (DeGeer, 1778: 653) *Tenebrio* [Bousquet & Bouchard, 2013: 45]

**Type data.** Syntypes (Naturhistoriska riksmuseet)

= *Sepidiumlacunosum* Thunberg, 1787: 48 [syn. by Haag-Rutenberg (1871a: 35)]

**Type data.** Syntypes (Uppsala University)

= *Brachyceruscristatus* Fabricius, 1798: 161 [syn. by Alonso-Zarazaga (2014: 73)]

**Type data.** Holotype (Lund University)

= *Brachycerusareolatus* Thunberg, 1799: 31 [syn. by Thunberg (1813: 399)]

**Type data.** Syntypes (Uppsala University)

= *Phligradegeeri* Laporte, 1840: 197 [syn. by Haag-Rutenberg (1871a: 35)]

**Type data.** Holotype (Paris Museum)

= *Cyrtoderescurculioides* Solier, 1843: 36 [syn. by Haag-Rutenberg (1871a: 35)]

**Type data.** Holotype (Paris Museum)

*cristatusnigritus* Solier, 1843: 35

**Type data.** Holotype (Paris Museum)

*cristatussinuosus* Solier, 1843: 34

**Type data.** Holotype (Paris Museum)

*crucifera* (Haag-Rutenberg, 1871a: 35) *Phligra* [Bousquet & Bouchard, 2013: 45]

**Type data.** Syntypes (Munich Museum – Haag-Rutenberg coll.)

*hamaticollis* (Péringuey, 1904: 235) *Phligra* [Bousquet & Bouchard, 2013: 45]

**Type data.** Syntypes (Cape Museum)

##### Genus *Epairopsis* Koch, 1955: 47

**Type species.***Trachynotusfrontalis* Haag-Rutenberg, 1873 (by original designation)

*deckerti* Ferrer, 2004: 193

**Type data.** Holotype (Berlin Museum)

*frontalisfrontalis* (Haag-Rutenberg, 1873: 43) *Trachynotus* [Koch, 1955: 47]

**Type data.** Syntypes (Naturhistoriska riksmuseet, Warsaw Museum)

= *Epairopslaevigata* Péringuey, 1892: 55 [syn. by Gebien (1920: 99)]

**Type data.** Syntypes (Cape Museum)

*frontalis variegata* (Péringuey, 1892: 55) *Epairops* [Gebien (1920: 99)]

**Type data.** Holotype (Cape Museum)

*superbus*Koch 1958: 217

**Type data.** Syntypes (Basel Museum, Budapest Museum, Ditsong Museum)

##### Genus *Ethmus* Haag-Rutenberg-Rutenberg, 1873: 44

**Type species.***Ethmusmaculatus* Haag-Rutenberg, 1873 (by subsequent designation by Gebien (1937a: 778))

= *Tynthlobia*Fairmaire 1888b: 261 [junior subjective synonym proposed by Fairmaire (1891a: 250)]

**Type species.***Tynthlobiaquadricostata* Fairmaire, 1888 (by monotypy)

###### Subgenus Ethmomerus Koch, 1954b: 243

**Type species.**Ethmus (Ethmomerus) subcylindricus Koch, 1954 (by original designation)

*brevis* Koch, 1954b: 261

**Type data.** Holotype (Tervuren Museum) and paratypes (Budapest Museum, Ditsong Museum, Tervuren Museum)

*subcylindricus* Koch, 1954b: 259

**Type data.** Holotype (British Museum) and paratypes (British Museum, Ditsong Museum)

###### Subgenus Ethmophobes Koch, 1954b: 244

**Type species.***Ethmuslatus* Haag-Rutenberg, 1873 (by original designation)

*hereroherero* Koch, 1958: 32

**Type data.** Holotype and paratypes (Ditsong Museum)

*hereropronamibensis* Koch, 1958: 32

**Type data.** Holotype and paratypes (Ditsong Museum)

*latusbisbicostatus* Koch, 1958: 30

**Type data.** Holotype (Ditsong Museum) and paratypes (Budapest Museum, Ditsong Museum)

*latuskaokoanus* Koch, 1958: 30

**Type data.** Holotype (Ditsong Museum) and paratypes (Budapest Museum, Ditsong Museum)

*latuslatus* Haag-Rutenberg, 1873: 45 [Koch 1954b: 244]

**Type data.** Holotype (Naturhistoriska riksmuseet)

*paradisiacusangolanus* Koch, 1958: 33

**Type data.** Holotype and paratypes (Ditsong Museum)

*paradisiacusformosus* Koch, 1958: 33

**Type data.** Holotype (Ditsong Museum)

*paradisiacusparadisiacus* Koch, 1958: 28

**Type data.** Holotype (Ditsong Museum) and paratypes (Budapest Museum, Ditsong Museum)

*vernayivernayi* Koch, 1958: 26

**Type data.** Holotype (Ditsong Museum) and paratypes (Basel Museum, Budapest Museum, Ditsong Museum)

*vernayireductus* Koch, 1958: 28

**Type data.** Holotypes and paratypes (Ditsong Museum)

*vernayimarginatus* Koch, 1958: 28

**Type data.** Holotype (Ditsong Museum)

###### Subgenus Ethmus Haag-Rutenberg, 1873: 44

**Type species.***Ethmusmaculatus* Haag-Rutenberg, 1873 (by subsequent designation by Gebien (1937a: 778))

*acinopoides* Koch, 1954b: 247

**Type data.** Holotype (Tervuren Museum) paratypes (Ditsong Museum, Tervuren Museum)

*analis* Gebien, 1910a: 150

**Type data.** Holotype (Basel Museum)

*barbosai* Koch, 1954b: 241

**Type data.** Holotype (Maputo Museum)

*borgesi* Koch, 1958: 21

**Type data.** Syntypes (Basel Museum, Budapest Museum, Ditsong Museum)

*cinereosparsus* Gebien, 1910a: 151

**Type data.** Syntypes (Basel Museum)

*dollmani* Koch, 1954b: 248

**Type data.** Holotype (British Museum) and paratypes (British Museum, Ditsong Museum)

*gedyei* Koch, 1954b: 252

**Type data.** Holotype (British Museum) and paratypes (British Museum, Ditsong Museum)

*maculatus* Haag-Rutenberg, 1873: 45

**Type data.** Syntypes (Paris Museum, Munich Museum – Haag-Rutenberg coll.)

*nyassicus* Koch, 1954b: 249

**Type data.** Holotype and paratypes (Munich Museum)

*plicatus* Müller, 1887: 300

**Type data.** Holotype (Leiden Museum)

*pygidialis* Koch, 1954b: 253

**Type data.** Holotype (Ditsong Museum)

*pustulatus* Koch, 1954b: 257

**Type data.** Holotype (National Congo) and paratypes (Budapest Museum, Ditsong Museum, National Congo)

*quadricostatus* (Fairmaire, 1888b: 261) *Tynthlobia* [Koch 1954b: 254]

**Type data.** Holotype (Leyden Museum)

*sculptus* Koch, 1954b: 255

**Type data.** Holotype (Munich Museum)

*silvanus* Koch, 1958: 22

**Type data.** Holotype (Ditsong Museum) and paratypes (Basel Museum, Budapest Museum, Ditsong Museum)

*tessellatus* Koch, 1958: 24

**Type data.** Holotype (Ditsong Museum)

##### incertae sedis

*incostatus* Gebien, 1910a: 150

**Type data.** Syntypes (Basel Museum, Tervuren Museum)

**Notes.** Species unknown to Koch (1954, 1958).

##### Genus *Histrionotus* Koch, 1955: 44

**Type species.***Trachynotuslightfooti* Péringuey, 1892 (by original designation)

*lightfooti* (Péringuey, 1892: 122) *Trachynotus* [Koch, 1955: 44]

**Type data.** Syntypes (Cape Museum)

*omercooperi* Koch, 1955: 43

**Type data.**Koch (1955) did not provide any details concerning the type series. Therefore, a lectotype designation is needed to fix the taxonomic status of the genus and the species. **Lectotype**, designated here, “Aus, Gt. Namaqualand. / 17.IX.1950 / C. Koch, G. van Son”. Paralectotypes (Basel Museum, British Museum, Tervuren Museum).

**Notes.** Although Koch (1955: 44) states that *Histrionotus* was described as a monotypical genus (for *lightfooti*) he intentionally describes *Histrionotusomercooperi* on the preceding page.

##### Genus *Microphligra* Koch, 1955: 47

**Type species.***Phligraminuta* Péringuey, 1904: (by original designation)

**Notes.** In caption of plate 9 in his paper from 1955 Koch introduced the second representative of *Microphligra*: “Microphligra (Paraphligra) succulentium (subg. nov. in litt. sp. nov. in litt.)”. A habitus of this beetle is presented in the preceding page. However, Koch did not specify any characters separating this new entity from the *Microphligraminuta*, therefore this cannot be treated as a valid description according to the regulations of the ICZN (1999).

*minuta* (Péringuey 1904: 236) *Phligra* [Koch, 1955: 47]

**Type data.** Holotype (Cape Museum)

##### Genus *Ossiporis* Pascoe, 1866: 451

**Type species.***Ossiporisterrena* Pascoe, 1866 (by monotypy)

= *Epairops* Fåhraeus, 1870: 282 [syn. by Gebien (1937b: 37)]

**Type species.***Epairopsfragilis* Fåhraeus, 1870 (by monotypy)

*angolensis* Koch, 1953b: 86

**Type data.** Holotype (British Museum) and paratype (Ditsong Museum)

*capeneri* Koch, 1953d: 7

**Type data.** Holotype (Ditsong Museum) and paratypes (British Museum)

*crenulimargo* Koch, 1953d: 26

**Type data.** Holotype (British Museum) and paratypes (British Museum, Brussels Museum, Ditsong Museum)

*marshalli* Koch, 1953d: 24

**Type data.** Holotype (British Museum)

*taterae* Koch, 1953d: 2

**Type data.** Holotype (Tervuren Museum)

*terrenachubbi* Koch, 1953d: 25

**Type data.** Holotype (Ditsong Museum) and paratypes (Durban Museum)

*terrenafragilis* (Fåhraeus, 1870: 282) *Epairops* [Gebien, 1937b: 37]

**Type data.** Lectotype, designated by Ferrer (2004a) (Naturhistoriska riksmuseet) and paralectotypes (British Museum, Naturhistoriska riksmuseet)

*terrenaterrena* Pascoe, 1866: 452

**Type data.** Holotype (Basel Museum, British Museum)

*terrenarhodesiana* Koch, 1953d: 8

**Type data.** Holotype (Ditsong Museum) and paratypes (British Museum, Ditsong Museum, Rhodesia Museum)

*terrenazoutpansbergensis* Koch, 1953d: 28

**Type data.** Holotype and paratypes (Ditsong Museum)

*undulicostis* Koch, 1953d: 6

**Type data.** Holotype (Rhodesia Museum)

##### Genus *Oxycerus* Koch, 1955:46

**Type species.***Trachynotusresolutus* Péringuey, 1904 (by original designation)

*resolutus* (Péringuey, 1904: 234) *Trachynotus* [Koch, 1955: 46]

**Type data.** Holotype (Ditsong Museum)

##### Genus *Somaticus* Hope, 1840: 117

**Type species.***Sepidiumrugosum* Fabricius, 1781 (by original designation)

###### Subgenus Acromaticus Koch, 1955: 143

**Type species.***Sepidiumacuminatum* Quensel, 1806 (by original designation); syn. of *Sepidiumstriatum* Thunberg, 1787

*adventitus* (Péringuey, 1899: 229) *Trachynotus* [Koch, 1955: 169]

**Type data.** Syntypes (Cape Museum)

*albanyensis* Koch, 1955: 164

**Type data.** Holotype (British Museum)

*algoensis* Koch, 1955: 153

**Type data.** Syntypes (Budapest Museum, Ditsong Museum)

*bisinuatus* Koch, 1955: 155

**Type data.** Syntypes (Ditsong Museum)

*caviventris* Koch, 1955: 158

**Type data.** Holotype (Ditsong Museum)

*cohaerens* Koch, 1955: 166

**Type data.** Syntypes (Budapest Museum, Ditsong Museum)

*corallipes* Koch, 1955: 157

**Type data.** Syntypes (Basel Museum, Budapest Museum, Ditsong Museum)

*dimorphus* Koch, 1955: 172

**Type data.** Syntypes (Basel Museum, Budapest Museum, Cape Museum)

*georgensis* Koch, 1955: 166

**Type data.** Holotype (Cape Museum)

*hirundo* Koch, 1955: 159

**Type data.** Holotype (Cape Museum) and paratypes (Cape Museum, Ditsong Museum)

**Notes.**Koch (1955: 159) designated a variety named “*rubripes*”. The author expressly gave it infrasubspecific rank, since he also designated taxa at the subspecies level. Therefore, according to art. 45.6.4. of the ICZN (1999) is should not be treated as a subspecies.

*karrooensis* Koch, 1955: 164

**Type data.** Syntypes (Basel Museum, Budapest Museum, Ditsong Museum)

*licinoides* (Haag-Rutenberg, 1873: 7) *Trachynotus* [Koch, 1955: 176]

**Type data.** Syntypes (Munich Museum)

= *Trachynotusrusticus* Péringuey, 1899: 300 [syn. by Gebien (1937a: 773)]

**Type data.** Syntypes (Cape Museum)

*malaisei* Koch, 1955: 172

**Type data.** Syntypes (Basel Museum, British Museum, Budapest Museum, Ditsong Museum)

*marlothi* Koch, 1955: 17

**Type data.** Holotype (Ditsong Museum)

*moerens* (Haag-Rutenberg, 1879: 294) *Trachynotus* [Koch, 1955: 162]

**Type data.** Holotype (British Museum)

**Notes.** Type deposition information after Koch (1955).

*namaquensis* Koch, 1955: 170

**Type data.** Syntypes (Basel Museum, Budapest Museum, Ditsong Museum)

*nitens* (Péringuey, 1899: 298) *Trachynotus* [Koch, 1955: 171]

**Type data.** Syntypes (Cape Museum)

*nollothensis* Koch, 1955: 158

**Type data.** Holotype (Cape Museum)

*praephallatusfrigidorae* Koch, 1955: 162

**Type data.** Syntypes (Ditsong Museum)

*praephallatuspraephallatus* Koch, 1955: 161

**Type data.** Holotype and paratype (Ditsong Museum)

*punctiger* (Haag-Rutenberg, 1873: 15) *Trachynotus* [Koch, 1955: 156]

**Type data.** Holotype (Museum Berlin)

*purcelli* Koch, 1955: 151

**Type data.** Syntypes (Cape Museum)

*saxicola* Koch, 1955: 177

**Type data.** Holotype (Ditsong Museum)

*scaber* (Haag-Rutenberg, 1873: 15) *Trachynotus* [Koch, 1955: 175]

**Type data.** Holotype (Munich Museum)

*striatus* (Thunberg, 1787: 48) *SepidiumSepidium* [Ferrer, 2009: 114]

**Type data.** Holotype (Uppsala University)

= *Sepidiumacuminatus* Quensel, 1806: 130 [syn. by Ferrer (2009: 114)]

**Type data.** Syntypes (Naturhistoriska riksmuseet)

*suturalifer* Koch, 1955: 151

**Type data.** Holotype (Cape Museum)

*transmontanus* Koch, 1955: 167

**Type data.** Holotype (Cape Museum)

*vansonianus* Koch, 1955: 154

**Type data.** Syntypes (Ditsong Museum)

###### Subgenus Bechuanitis Koch, 1955: 93

**Type species.***Trachynotusbrucki* Haag-Rutenberg, 1873 (by original designation)

*Trachynotusbohemanibohemani* (Haag-Rutenberg, 1873: 11) *Trachynotus* [Koch, 1955: 94]

**Type data.** Holotype (Munich Museum) and paratype (Naturhistoriska riksmuseet)

**Notes.** Type deposition information after Koch (1955).

*bohemanigaerdesi* Koch, 1955: 95

**Type data.** Holotype (Ditsong Museum)

*bohemanischerzi* Koch, 1955: 96

**Type data.** Syntypes (Ditsong Museum)

*bruckibrucki* (Haag-Rutenberg, 1873: 13) *Trachynotus* [Koch, 1955: 93]

**Type data.** Holotype (Naturhistoriska riksmuseet)

**Notes.** Type deposition information after Koch (1955).

*bruckiovamboanus* Koch, 1955: 104

**Type data.** Holotype (Ditsong Museum)

*bruckipoweri* (Hesse, 1935: 554) *Trachynotus* [Koch, 1955: 104]

**Type data.** Holotype (Cape Museum)

**Notes.** Type deposition information after Koch (1955).

*cinctus* (Haag-Rutenberg, 1873: 12) *Trachynotus* [Koch, 1955: 97]

**Type data.** Holotype (Naturhistoriska riksmuseet)

**Notes.** Type deposition information after Koch (1955).

*geniculatusgeniculatus* (Haag-Rutenberg, 1873: 21) *Trachynotus* [Koch, 1955: 99]

**Type data.** Syntypes (British Museum, Munich Museum)

*geniculatushessei* Koch, 1955: 101

**Type data.** Syntypes (Budapest Museum, Ditsong Museum)

*geniculatuspluricostatus* Koch, 1955: 100

**Type data.** Syntypes (Ditsong Museum, Munich Museum)

*hereroensis* Koch, 1955: 99

**Type data.** Holotype (University Lund)

*rugulosicollis* (Hesse, 1935: 554) *Trachynotus* [Koch, 1955: 102]

**Type data.** Syntypes (Cape Museum, Ditsong Museum)

###### Subgenus Ceromelaephus Koch, 1955: 87

**Type species.***Trachynotusbadeni* Haag-Rutenberg, 1873 (by original designation)

*badeni* (Haag-Rutenberg, 1873: 10) *Trachynotus* [Koch, 1955: 87]

**Type data.** Holotype (Munich Museum – Haag-Rutenberg coll.)

= *Trachynotusscrobiculatus* Péringuey, 1885: 110 [syn. by Gebien (1937a: 773)]

**Type data.** Holotype (Cape Museum)

*seminitidus* Koch, 1955: 89

**Type data.** Holotype (Ditsong Museum) and paratypes (Budapest Museum, Cape Museum, Naturhistoriska riksmuseum)

*Trachynotusstrangulatusarborarius* Koch, 1955: 92

**Type data.** Syntypes (Budapest Museum, Ditsong Museum)

*Trachynotusstrangulatusauobensis* Koch, 1955: 93

**Type data.** Holotype and paratypes (Ditsong Museum)

*Trachynotusstrangulatuspatzelti* Koch, 1955: 92

**Type data.** Holotype (Ditsong Museum) and paratype (California Academy of Science)

*Trachynotusstrangulatusrehobothensis* Koch, 1955: 92

**Type data.** Holotype (Ditsong Museum) and paratypes (Cape Museum)

*Trachynotusstrangulatusstrangulatus* (Gebien, 1920: 92) *Trachynotus* [Koch, 1955: 90]

**Type data.** Holotype (Basel Museum)

*Trachynotuswahlbergiausensis* Koch, 1955: 90

**Type data.** Holotype (Cape Museum) and paratype (Ditsong Museum)

*Trachynotuswahlbergiwahlbergi* (Haag-Rutenberg, 1873: 10) *Trachynotus* [Koch, 1955: 89]

**Type data.** Holotype (Naturhistoriska riksmuseum)

**Notes.** Type deposition information after Koch (1955).

###### Subgenus Clinocranion Solier, 1843: 114

**Notes.** While describing this genus Solier (1843) used two following forms of the name, *Clinocranion* and *Clynocranion*. Koch (1955: 70), the first reviewer, selected the first one.

Treated as a subgenus of *Trachynotus* (e.g., Gebien 1920), while the current interpretation proposed by Koch (1955).

**Type species.***Clinocranionspinosum* Solier, 1843 (by subsequent designation by Lucas (1920: 190))

*planatusdrukeri* Koch, 1955: 73

**Type data.** Syntypes (Budapest Museum, Ditsong Museum, Tervuren Museum)

*planatusplanatus* (Solier, 1843: 116) *Clinocranion* [Koch, 1955: 72]

**Type data.** Holotype (Geneva Museum – Gory collection)

**Notes.** Type deposition information after Koch (1955).

*planatussubdamarensis* Koch, 1955: 73

**Type data.** Holotypes and paratypes (Ditsong Museum)

*spinosus* (Solier, 1843: 115) *Clinocranion* [Koch, 1955: 71]

**Type data.** Holotype (Geneva Museum)

###### Subgenus Diacis Koch, 1955: 105

**Type species.***Trachynotusregalis* Haag-Rutenberg, 1875 (original designation)

*angustus* (Péringuey, 1886: 125) *Trachynotus* [Koch, 1955: 106]

**Type data.** Syntypes (Cape Museum)

*distinctus* (Péringuey, 1892: 54) *Trachynotus* [Koch, 1955: 105]

**Type data.** Syntypes (Cape Museum)

*regalis* (Haag-Rutenberg, 1875: 82) *Trachynotus* [Koch, 1955: 106]

**Type data.** Syntypes (British Museum, Munich Museum – Haag-Rutenberg coll.)

###### Subgenus Somaticus Hope, 1840: 117

**Notes.** Although the correct original spelling of this genus group name is *Somaticum* (Hope, 1840: 117 and errata), to our knowledge all subsequent authors have used the incorrect subsequent spelling *Somaticus* and that this incorrect subsequent spelling is in prevailing usage and attributed to the publication of the original spelling; therefore this genus-group name is to be preserved and deemed to be the correct original spelling (ICZN 1999, Art. 33.3.1).

**Type species.***Sepidiumrugosum* Fabricius, 1781 (by original designation)

= *Gonopterus* Solier, 1843: 101 [junior subjective synonym proposed by Gebien, 1910b: 163]

**Type species.***Sepidiumrugosum* Fabricius, 1781 (by monotypy)

*aeneus* (Solier, 1843: 111) *Trachynotus* [Koch, 1955: 82]

**Type data.** Syntypes (Geneva Museum)

**Notes.** Type deposition information after Koch (1955).

*bisbicostatus* (Gebien, 1920: 93) *Trachynotus* [Koch, 1955: 86]

**Type data.** Neotype (Basel Museum), designated by Koch (1955: 86)

**Notes.** According to Koch (1955) the holotype was originally preserved in the Museum Hamburg, but was destroyed during the World War II.

*decoratipescisfluminis* Koch, 1955: 81

**Type data.** Syntypes (Budapest Museum, Ditsong Museum)

*decoratipesdecoratipes* Koch, 1955: 79

**Type data.** Syntypes (Basel Museum, Budapest Museum, Cape Museum, Ditsong Museum)

*glabriventris* Koch, 1955: 78

**Type data.** Syntypes (Basel Museum, Budapest Museum, Paris Museum)

*rugosusrugosissimus* Koch, 1955: 78

**Type data.** Syntypes (Cape Museum)

*rugosusrugosus* (Fabricius, 1781: 315) *Sepidium* [Koch, 1955: 76]

**Type data.** Holotype Copenhagen Museum) and paratypes (British Museum)

**Notes.** Type deposition information after Koch (1955)

= *Pimelialeucophrys* Herbst, 1799: 115 [syn. by Haag-Rutenberg, 1873: 8]

**Type data.** Holotype (Berlin Museum)

*rugosustestaceipes* Koch, 1955: 77

**Type data.** Syntypes (Ditsong Museum)

*stali* (Haag-Rutenberg, 1873: 18) *Trachynotus* [Koch, 1955: 85]

**Type data.** Holotype (Naturhistoriska riksmuseet)

= *Clinocranionlatemarginatum* Péringuey, 1885: 115 [syn. by Gebien (1910b: 165)]

**Type data.** Holotype (Cape Museum)

*straminicornis* Koch, 1955: 81

**Type data.** Holotype (Ditsong Museum)

*stridulatus* Koch, 1955: 78

**Type data.** Syntypes (Budapest Museum, California Academy, Ditsong Museum, Stellenbosch University)

*welwitschi* Koch, 1955: 84

**Type data.** Syntypes (British Museum)

###### Subgenus Tracheloeum Hope, 1840: 116

**Type species.***Tracheloeumlaticolle* Hope, 1840 (by original designation)

*carinatuscancellatus* Koch, 1955: 200

**Type data.** Holotype (Ditsong Museum)

*carinatuscarinatus* (Solier, 1843: 109) *Trachynotus* [Koch, 1955: 199]

**Type data.** Holotype (Geneva Museum)

**Notes.** Type deposition information after Haag-Rutenberg (1871).

*carinatuschevrolati* (Haag-Rutenberg, 1873: 35) *Trachynotus* [Koch, 1955: 54]

**Type data.** Syntypes (Munich Museum – Haag-Rutenberg coll.)

*contractus* (Haag-Rutenberg, 1873: 27) *Trachynotus* [Koch, 1955: 189]

**Type data.** Holotype (Museum Berlin)

**Notes.** Type deposition information after Koch (1955).

*dilatatus* (Haag-Rutenberg, 1873: 25) *Trachynotus* [Koch, 1955: 182]

**Type data.** Holotype (Munich Museum)

*fahraeusi* Koch, 1955: 185

**Type data.** Syntypes (Cape Museum)

**Notes.**Koch (1955) designates a variety “*M-signatus*”. Judging from the context, Koch expressively gave it the infrasubspecific rank. Therefore, according to art. 45.6.4. of the ICZN (1999) is should not be treated as a subspecies.

*giganteus* Koch, 1955: 194

**Type data.** Syntypes (Budapest Museum, Ditsong Museum)

*hoffmanni* (Haag-Rutenberg, 1878: 93) *Trachynotus* [Koch, 1955: 201]

**Type data.** Holotype (Stuttgart Museum)

*impressicollis* (Péringuey, 1885: 111) *Trachynotus* [Koch, 1955: 200]

**Type data.** Syntypes (Cape Museum)

*intermedius* (Haag-Rutenberg, 1878: 92) *Trachynotus* [Koch, 1955: 195]

**Type data.** Holotype (Munich Museum – Haag-Rutenberg coll.)

*laticollis* (Hope, 1840: 116) *Tracheloeum* [Koch, 1955: 179]

**Type data.** Holotype (Oxford University – Hope coll.)

**Notes.** Type deposition information after Koch (1955).

*maculosus* (Fåhraeus, 1870: 276) *Trachynotus* [Koch, 1955: 184]

**Type data.** Syntypes (Naturhistoriska riksmuseet)

**Notes.** Type deposition information after Koch (1955).

*marginatus* (Thunberg, 1787) *Sepidium* [Ferrer: 2009: 114]

**Type data.** Holotype (Uppsala University)

= *Trachynotuslaevis* Fåhraeus, 1870: 275 [syn. by Ferrer, 2009: 114]

**Type data.** Syntypes (Naturhistoriska riksmuseet, Warsaw Museum)

**Notes.** Type deposition information after Koch (1955).

= *Trachnotusglaber* Fåhraeus, 1870: 275 [syn. by Koch (1955: 181)]

**Type data.** Syntypes (Naturhistoriska riksmuseet)

*pretorianus bushveldeus* Koch, 1955: 183

**Type data.** Holotype (Ditsong Museum) and paratype (Cape Museum)

*pretorianuspretorianus* Koch, 1955: 182

**Type data.** Syntypes (Basel Museum, Budapest Museum, Ditsong Museum)

*silphoidesmetallescens* Koch, 1955: 194

**Type data.** Holotype (South African National Collection)

*silphoidesnigronitens* Koch, 1955: 193

**Type data.** Syntypes (Cape Museum, Ditsong Museum)

*silphoidesperingueyi* Koch, 1955: 191, replacement name

= *Trachynotusplicipennis* Péringuey, 1899: 300 [junior primary homonym of *Trachynotusplicipennis* Haag-Rutenberg, 1873: 38]

**Type data.** Syntypes (Cape Museum)

*silphoidessilphoides* (Fåhraeus, 1870: 274) *Trachynotus* [Koch, 1955: 191]

**Type data.** Holotype (Naturhistoriska riksmuseet)

**Notes.** Type deposition information after Koch (1955).

*silphoidesswazicola* Koch, 1955: 193

**Type data.** Syntypes (Munich Museum)

*similis* (Haag-Rutenberg, 1873: 35) *Trachynotus* [Koch, 1955: 196]

**Type data.** Holotype (Munich Museum – Haag-Rutenberg coll.)

*transvaalensis* Koch, 1955: 197

**Type data.** Syntypes (Cape Museum, Ditsong Museum)

*vittiger* (Haag-Rutenberg, 1873: 22) *Trachynotus* [Koch, 1955: 187]

**Type data.** Holotype (British Museum)

**Notes.** Type deposition information after Koch (1955).

###### Subgenus Trachyderes Koch, 1955: 112

**Type species.***Trachynotusbipunctatus* Haag-Rutenberg, 1873 (by original designation)

*albomaculatus* (Haag-Rutenberg, 1873: 41) *Trachynotus* [Koch, 1955: 142]

**Type data.** Holotype (Munich Museum – Haag-Rutenberg coll.)

= *Trachynotusterrenus* Péringuey, 1885: 114 [syn. by Gebien, 1937a: 773]

**Type data.** Syntypes (Cape Museum)

*barnardi* Koch, 1955: 137

**Type data.** Holotype (Cape Museum)

*bipunctatusbipunctatus* (Haag-Rutenberg, 1873: 20) *Trachynotus* [Koch, 1955: 113]

**Type data.** Syntypes (Munich Museum – Haag-Rutenberg coll.)

*bipunctatus pilosus* (Péringuey, 1885: 112) *Trachynotus* [Koch, 1955: 121]

**Type data.** Syntypes (Cape Museum)

*braunsi* Koch, 1955: 135

**Type data.** Syntypes (Basel Museum, British Museum, Budapest Museum, Ditsong Museum, Tervuren Museum)

*cordipennis* Koch, 1955: 136

**Type data.** Holotype (Ditsong Museum)

*dubius* (Péringuey, 1885: 114) *Trachynotus* [Koch, 1955: 130]

**Type data.** Holotype (Cape Museum)

= *Trachynotussericeus* Péringuey, 1886: 124 [syn. by Gebien, 1937a: 773]

**Type data.** Syntypes (Cape Museum)

= *Trachynotusdubiusmaculipennis* Gebien, 1920: 99 [syn. by Koch 1955: 130]

**Type data.** Syntypes (Hamburg University)

*dutoiti* Koch, 1955: 118

**Type data.** Syntypes (Basel Museum, Budapest Museum, Ditsong Museum)

*eremicola* Koch, 1955: 129

**Type data.** Syntypes (Cape Museum, Ditsong Museum)

*fitzsimonsi* Koch, 1955: 126

**Type data.** Holotype (Ditsong Museum) and paratypes (Budapest Museum, Ditsong Museum)

*goryi* (Solier, 1843: 112) *Trachynotus* [Koch, 1955: 117]

**Type data.** Holotype (Geneva Museum)

**Notes.** Type deposition information after Koch (1955).

*gracilipes* (Haag-Rutenberg, 1873: 19) *Trachynotus* [Koch, 1955: 128]

**Type data.** Syntypes (Munich Museum – Haag-Rutenberg coll.)

= *Trachynotusattenuatus*Péringuey 1886: 125 [syn. by Gebien, 1910b: 164]

**Type data.** Syntypes (Cape Museum)

*gunvoraeamnigenus* Koch, 1955: 123

**Type data.** Holotype (Cape Museum)

*gunvoraecylindricollis* Koch, 1955: 122

**Type data.** Holotype (Cape Museum)

*gunvoraegunvorae* (Koch, 1953c: 12) *Trachynotus* [Koch, 1955: 121]

**Type data.** Holotype (Lund University) and paratypes (Ditsong Museum, Lund University)

*haagihaagi* (Péringuey, 1899: 299) *Trachynotus* [Koch, 1955: 114]

**Type data.** Holotype (Cape Museum)

*haagipilipeplus* Koch, 1955: 116

**Type data.** Holotype (Ditsong Museum) and paratype (Naturhistoriska riksmuseet)

*incostatus* (Gebien, 1920: 99) *Trachynotus* [Koch, 1955: 138]

**Type data.** Syntypes (Basel Museum)

*kungorum* Koch, 1955: 140

**Type data.** Syntypes (Ditsong Museum)

*plutus* Koch, 1955: 134

**Type data.** Holotype (Ditsong Museum) and paratypes (California Academy, Ditsong Museum, Munich Museum)

*pygmaeus* (Fåhraeus, 1870: 279) *Trachynotus* [Koch, 1955: 141]

**Type data.** Holotype (Naturhistoriska riksmuseet)

= *Trachynotustantillus* Péringuey, 1899: 301 [syn. by Péringuey (1904: 297)]

**Type data.** Holotype (Cape Museum)

*ratus* Koch, 1955: 125

**Type data.** Syntypes (Basel Museum, Budapest Museum, Ditsong Museum)

*tentyrioides* (Haag-Rutenberg, 1873: 24) *Trachynotus* [Koch, 1955: 124]

**Type data.** Holotype (Munich Museum)

= *Trachynotusacuticostis*Gebien 1920: 97 [syn. by Koch (1955: 124)]

**Type data.** Syntypes (Basel Museum)

*tibialis* (Haag-Rutenberg, 1873: 20) *Trachynotus* [Koch, 1955: 132]

**Type data.** Holotype (Munich Museum)

**Notes.**Koch (1955) designates a variety “*nigripes*”. Judging from the context, Koch expressively give it the infrasubspecific rank. Therefore, according to art. 45.6.4. of the ICZN (1999) is should not be treated as a subspecies.

*zinni* Koch, 1955: 133

**Type data.** Syntypes (Cape Museum)

###### Subgenus Trichotrachys Koch, 1955: 201

**Type species.***Trachynotussordidus* Gerstaecker, 1854 (by original designation)

*angulatus* (Fåhraeus, 1870: 277) *Trachynotus* [Koch, 1955: 206]

**Type data.** Holotype (Naturhistoriska riksmuseet)

**Notes.** Type deposition information after Koch (1955).

*darlingtoni* Koch, 1955: 212

**Type data.** Syntypes (Budapest Museum, Ditsong Museum)

*funestus* (Fåhraeus, 1870: 278) *Trachynotus* [Koch, 1955: 227]

**Type data.** Holotype (Naturhistoriska riksmuseet)

**Notes.** Type deposition information after Koch (1955).

*griseus* (Fåhraeus, 1870: 277) *Trachynotus* [Koch, 1955: 214]

**Type data.** Holotype (Naturhistoriska riksmuseet)

**Notes.** Type deposition information after Koch (1955).

*hispidus* (Hesse, 1935: 555) *Trachynotus* [Koch, 1955: 225]

**Type data.** Syntype (Cape Museum, Ditsong Museum)

*histrio* Koch, 1955: 229

**Type data.** Holotype (Cape Museum)

*lutulentuslutulentus* (Péringuey, 1899: 301) *Trachynotus* [Koch, 1955: 222]

**Type data.** Holotype (Cape Museum)

*lutulentusmontisdraconis* Koch, 1955: 223

**Type data.** Syntypes (Durban Museum)

*metropolis* Koch, 1955: 227

**Type data.** Syntypes (Basel Museum, British Museum, Budapest Museum, Ditsong Museum)

*newtoni* Koch, 1955: 223

**Type data.** Holotype (Ditsong Museum)

*obscurus* Koch, 1955: 208

**Type data.** Syntypes (Cape Museum)

*schalkwykae* Koch, 1955: 213

**Type data.** Syntypes (Pretoria University)

*sinuatus* Koch, 1955: 208

**Type data.** Holotype (Ditsong Museum)

*sordidus* (Gerstaecker, 1854: 532) *Trachynotus* [Koch, 1955: 205]

**Type data.** Syntypes (Berlin Museum)

*terricolasetulosus* (Haag-Rutenberg, 1873: 31) *Trachynotus* [Koch, 1955: 216]

**Type data.** Syntypes (Munich Museum, Warsaw Museum)

**Notes.** Type deposition information after Koch (1955).

*terricolaterricola* (Fåhraeus, 1870: 278) *Trachynotus* [Koch, 1955: 215]

**Type data.** Syntypes (Naturhistoriska riksmuseet)

**Notes.** Type deposition information after Koch (1955).

*testudo* Koch, 1955: 220

**Type data.** Holotype (Cape Museum)

*varicollisbrachythorax* Koch, 1955: 211

**Type data.** Syntypes (Budapest Museum, Ditsong Museum)

*varicollisdisconnectus* Koch, 1955: 212

**Type data.** Holotype (Cape Museum) and paratypes (Cape Museum, Ditsong Museum)

*varicollisvaricollis* Koch, 1955: 209

**Type data.** Syntypes (Budapest Museum, Ditsong Museum)

*vestitus* (Haag-Rutenberg, 1873: 30) *Trachynotus* [Koch, 1955: 218]

**Type data.** Holotype (Munich Museum – Haag-Rutenberg coll.)

*zumptirhodesianus* Koch, 1955: 220

**Type data.** Holotype (Ditsong Museum) and paratype (Cape Museum)

*zumptizumpti* Koch, 1955: 219

**Type data.** Syntypes (Budapest Museum, Ditsong Museum)

###### Subgenus Trichotrichus Koch, 1955: 108

**Type species.***Trachynotuscrinitus* Haag-Rutenberg, 1873 (by original designation)

*crinitus* (Haag-Rutenberg, 1873: 32) *Trachynotus* [Koch, 1955: 109]

**Type data.** Holotype (Munich Museum – Haag-Rutenberg coll.)

*kraatzikraatzi* (Haag-Rutenberg, 1873: 33) *Trachynotus* [Koch, 1955: 110]

**Type data.** Holotype (Munich Museum – Haag-Rutenberg coll.)

*kraatzifulvohirtus* Koch, 1955: 111

**Type data.** Syntypes (Basel Museum, Budapest Museum, Ditsong Museum)

*kraatziorientalis* Koch, 1955: 112

**Type data.** Holotype (Ditsong Museum) and paratypes (Stellenbosch University)

*serratus* (Péringuey, 1885: 112) *Trachynotus* [Koch, 1955: 112]

**Type data.** Holotype (Cape Museum)

###### Subgenus Tropitrachys Koch, 1955: 229

**Type species.***Trachynotusperegrinator* Koch, 1953a (by original designation)

*peregrinator* (Koch, 1953a: 179) *Trachynotus* [Koch, 1955: 230]

**Type data.** Holotype (British Museum) and paratypes (British Museum, Budapest Museum, Ditsong Museum)

*tropicalis* Koch, 1955: 231

**Type data.** Holotype (Frankfurt Museum)

incertae sedis

*damarinus* Péringuey, 1904: 233 [Ferrer 2000: 145]

**Type data.** Holotype (Naturhistoriska riksmuseet)

**Notes.**Ferrer (2000) transferred this species to the genus *Somaticus*. However, he treated “*Trachynotideus*” as a valid generic name and interpreted it as a subgenus within *Somaticus*. This view is not shared here (see comments to the genus *Trachynotidus*) therefore *damarinus* is considered *incertae sedis*.

*scutelliformis* (Laporte, 1840: 197) *Sepidium*

**Type data.** Holotype (Munich Museum)

**Notes.** See Koch (1955: 56) for details.

##### Genus *Trachynotus* Latreille, 1828: 579

**Type species.***Sepidiumvittatus* Fabricius, 1781 (**here designated**)

**Notes.***Trachynotus* used to be attributed to Latreille (1829: 14). In that publication, *Sepidiumreticulatum* was one of the originally included species and this species was later selected as the type species (see Hope 1840: 115). However, the present literature investigation revealed that the name *Trachynotus* was made available earlier (Latreille 1828). Three following species were originally included: *acuminatus* Quensel, 1806 (currently classified in *Somaticus*), *clathratum* (attributed to Fabricius), and *vittatum* Fabricius, 1781. In order to provide the nomenclatural stability the last taxon is hereby designated as a type species of *Trachynotus*.

= *Hipomelus* Dejean, 1834: 181 [junior objective synonym; see Bousquet & Bouchard (2013: 47)]

**Type species.***Sepidiumvittatum* Fabricius, 1781 (by subsequent designation by Hope (1840))

*albulus* Péringuey, 1886: 127

**Type data.** Syntypes (Cape Museum)

*elongatus* (Olivier, 1795: 8) *Sepidium* [Haag-Rutenberg, 1873: 36]

**Type data.** Syntypes (Paris Museum)

*leucographus* Solier, 1843: 107

**Type data.** Syntypes (Paris Museum)

*lutosus* Péringuey, 1885: 113

**Type data.** Syntypes (Cape Museum)

*meracus* Péringuey, 1899: 302

**Type data.** Holotype (Cape Museum)

*ornatus* Haag-Rutenberg, 1873: 40

**Type data.** Holotype (Munich Museum – Haag-Rutenberg coll.)

*plicipennis* Haag-Rutenberg, 1873: 38

**Type data.** Holotype (Munich Museum – Haag-Rutenberg coll.)

*proximus* Laporte, 1840: 197

**Type data.** Holotype (Paris Museum)

*recurvus* Haag-Rutenberg, 1873: 38

**Type data.** Holotype (Munich Museum – Haag-Rutenberg coll.)

*reticulatus* (De Geer, 1778: 651) *Tenebrio* [Haag-Rutenberg, 1873: 2]

**Type data.** Holotype (Naturhistoriska riksmuseet)

= *Sepidiumreticulatum* Thunberg, 1791: 23 [syn. by Ferrer (2009: 116)]

**Type data.** Syntypes (Uppsala University)

*sctulosus* Haag-Rutenberg, 1873: 31

**Type data.** Syntypes (Munich Museum – Haag-Rutenberg coll.)

*tricostatus* Haag-Rutenberg, 1873: 23

**Type data.** Holotype (Munich Museum – Haag-Rutenberg coll.)

*variegatus* Haag-Rutenberg, 1878: 94

**Type data.** Holotype (Munich Museum – Haag-Rutenberg coll.)

*albulusvicinus* (Haag-Rutenberg, 1871b: 51) *Psammodes* [Koch, 1955: 46]

**Type data.** Syntypes (Geneva Museum, Warsaw Museum)

*vittatus* (Fabricius, 1781: 315) *Sepidium* [Haag-Rutenberg, 1873: 5]

**Type data.** Syntypes (British Museum, Copenhagen Museum)

= *Sepidiumvittatum* Thunberg, 1791: 24 [syn. by Ferrer (2009: 117)]

**Type data.** Syntypes (Uppsala University)

= *Sepidiumplicatus* Wiedemann, 1823: 39 [syn. by Koch (1955: 46)]

**Type data.** Holotype (Humboldt University)

= *Trachynotuslacunosus* Solier, 1843: 110 [syn. by Haag-Rutenberg (1873: 39)]

**Type data.** Syntypes (Paris Museum)

##### Genus *Trichethmus* Gebien, 1937b: 45

**Type species.***Trichethmuslobicolis* Gebien, 1937b: 45 (by monotypy)

*lobicolis* Gebien, 1937b: 46

**Type data.** Holotype (Basel Museum)

## Supplementary materials

Supplementary material 1 Analysed distributional data in CSV format Data type: Distributional data. This dataset is made available under the Open Database License (http://opendatacommons.org/licenses/odbl/1.0/). The Open Database License (ODbL) is a license agreement intended to allow users to freely share, modify, and use this Dataset while maintaining this same freedom for others, provided that the original source and author(s) are credited.Marcin J. Kamiński, Kojun Kanda, Ryan Lumen, Jonah M. Ulmer, Christopher C. Wirth, Patrice Bouchard, Rolf Aalbu, Noël Mal, Aaron D. Smith

## References

[B1] Agassiz L (1846) Nomenclatoris Zoologici Index Universalis. Jent et Gassmann, Soloduri, 1135 pp.

[B2] Allard E (1870) [new species description]. Petites Nouvelles Entomologiques 13: 49–50.

[B3] Allard E (1874) Mémoire sur les Coléoptères Ténébrionides formant les genres *Sepidium* , Fabr. & Vieta, Cast. Revue et Magasin de Zoologie 3: 120–151.

[B4] Allard E (1882) Communications. Bulletin de la Société Entomologique de France 6: 87.

[B5] Amyot CJB (1835) M. de Pierret. M. Pierreti. Seville. Magazin de Zoologie 9: pl. 129.

[B6] Ancey CF (1881a) Descriptions de Coléoptères nouveaux d’Aden. Le Naturaliste 50: 39.

[B7] Ancey CF (1881b) Descriptions de Coléoptères nouveaux. Le Naturaliste 58: 461–462.

[B8] Ancey CF (1883) Contributions à la faune de l’Afrique Orientale Descriptions de Coléoptères nouveaux. Il Naturalista Siciliano 2: 116–120.

[B9] Antoine M (1932) Notes d’entomologie Marocaine. Bulletin de la Société des Sciences Naturelles du Maroc 12: 173–188.

[B10] Antoine M (1951a) Notes d’entomologie Marocaine. Bulletin de la Société des sciences naturelles et physiques du Maroc 30: 87–101.

[B11] Ardoin P (1977) ColeopteraTenebrionidae. Scientific report of the Belgian Mt. Kenya bio-expedition, 1975. no 10. Revue de Zoologie Africaine 91(4): 811–816.

[B12] Ardoin P (1979) Mission Balachowsky-Menier dan l’ancien territoire Français des Afars et des Issas. Bulletin de la Société Entomologique de France 8 4: 58–61.

[B13] Baudi F (1875) Catalogo dei Tenebrioniti della fauna Europea e circummediterranea appartenenti alle collezioni del Museo Civico di Genova. Annali del Museo Civico di Storia Naturale di Genova 7: 684–703.

[B14] Baudi F (1876) Europaea et circummediterraneae faunae Tenebrionidum specierum, quae Comes Dejean in suo Catalogo, editio 3a, consignavit, ex ejusdem collectione in R. Taurinensi Musaeo asservata, cum auctorum hodierne recepta denominatione collatio. Deutsche Entomologische Zeitschrift 20: 1–74.

[B15] Bertoloni J (1849) Illustratio rerum naturalium Mozambici. Dissertatio 1 de Coleopteris. Novi Commentarii Academiae Scientiarum Instituti Bononiensis 10: 381–434.

[B16] Bezděk J Regalin R (2015) Identity of species-group taxa of the Western Palaearctic Clytrini (Coleoptera: Chrysomelidae) described by Maurice Pic and Louis Kocher. Acta Entomologica Musei Nationalis Pragae 55(supplement): 1–113.

[B17] Billberg CJ (1815) Insecta ex ordine coleopterorum descripta. Uppsala Kungliga Vetenskapliga Sällskapet. Nova Acta 2: 271–281.

[B18] Boheman CH (1847) Arsberattelse om Zoologiens Framsteg under Aren 1845 och 1846 till Kongl. Vetenskaps Akademien AFGIFVEN af Zoologie Intendenterna. Andra Delen (Insecta. Linn.). Stockholm, 276 pp.

[B19] Bouchard P Lawrence JF Davies A Newton AF (2005) Synoptic classification of the world Tenebrionidae (Insecta: Coleoptera) with a review of family-group names. Annales Zoologici 55(4): 499–530.

[B20] Bouchard P Bousquet Y Davies AE Alonso-Zarazaga MA Lawrence JF Lyal CHC Newton AF Reid CAM Schmitt M Ślipiński SA Smith ABT (2011) Family-group names in Coleoptera (Insecta). ZooKeys 88: 1–972. 10.3897/zookeys.88.807 PMC3088472 21594053

[B21] Bousquet Y (2016) Litteratura Coleopterologica (1758–1900): a guide to selected books related to the taxonomy of Coleoptera with publication dates and notes. ZooKeys 583: 1–776. 10.3897/zookeys.583.7084

[B22] Bousquet Y Bouchard P (2013) The genera in the second catalogue (1833–1836) of Dejean’s Coleoptera collection. ZooKeys 282: 1–219. 10.3897/zookeys.282.4401 PMC3677338 23794836

[B23] Brancsik K (1914) Coleoptera nova. Bericht des Museumvereines für das Comitat Trencsén 1914: 58–69.

[B24] Burchell WJ (1822) Travels in the Interior of Southern Africa. Longman, Hurst, Rees, Orme, and Brown, London, 586 pp. 10.5962/bhl.title.100911

[B25] Chambers N (2000) The letters of Sir Joseph Banks: a selection, 1768–1820. Imperial College Press, London, xliii + 420 pp. 10.1142/9781848160262

[B26] Champion GC (1895) A list of Tenebrionidae supplementary to the” Munich” Catalogue. Mémoires de la Société Entomologiques de Belgique 3: 1–264.

[B27] Chevrolat (1874) Nouvelle espèce d’Échinotus. Genre voisin de *Sepidium* . Revue et Magasin de Zoologie 3: 331.

[B28] Conci C Poggi R (1996) Iconography of Italian entomologists, with essential biographical data. Memorie della Società Entomologica Italiana, Genoa 75: 159–382.

[B29] Copenhagen Museum (2019) Official website accessed on February 28, 2019. https://samlinger.snm.ku.dk/en/dry-and-wet-collections/zoology/entomology/fabricius-collection/

[B30] Crotch GR (1872) List of the Coleoptera found during the Progress of the Survey. In: Wilson CW Palmer HS (Eds) Ordnance Survey of the Peninsula of Sinai.Vol. 1. Ordnance Survey Office, Southampton, 263–268.

[B31] DeGeer C (1778) Mémoires pour servir a l’histoire des insectes. Stockholm. Dixième Mémoire: 591–666.

[B32] Dejean PFMA (1834) Catalogue des Coléoptères de la collection de M. le Comte Dejean. [Livraison 3]. Méquignon-Marvis, Paris, 177–256. 10.5962/bhl.title.8771

[B33] Desbrochers des Loges J (1881) Insectes coléoptères du nord de l’Afrique nouveaux ou peu connus. ler Mémoire. Ténébrionides. Bulletin de I’Academie d’Hippone 16: 51–168.

[B34] Distant WL (1892) A Naturalist in the Transvaal. R. H. Porter, London, 277 pp. 10.5962/bhl.title.29549

[B35] Doyen JT (1994) Cladistic relationships among Pimeliine Tenebrionidae (Coleoptera). Journal of the New York Entomological Society 101: 443–514.

[B36] Ekis G (1975) Taxonomic and nomenclatural status of clerid taxa described by Massimiliano Spinola (1780–1857) (Coleoptera: Cleridae). Bolletino del Museo di Zoologia dell’ Università di Torino 1975(1): 1–80.

[B37] Erichson WF (1841) Über die Insecten von Algier mit besonderer Rücksicht auf ihre geographische Verbreitung. In: Wagner MF (Ed.) Reisen in der Regentschaft Algier 1836, 1837 und 1838 nebst einem naturhistorischen Anhang und einem Kupferatlas.Dritter Band. L. Voss, Leipzig, 140–194.

[B38] Erichson WF (1843) Beitrag zur Insecten-Fauna von Angola, in besonderer Beziehung zur geographischen Verbreitung der Insecten in Africa. Archiv für Naturgeschichte 9: 199–267.

[B39] Erichson WF (1844) Entomology, Coleoptera. In: Ray Society, Reports on zoology for 1843, 1844. Instituted MDCCCXLIV, London, 313–355.

[B40] Escalera MM (1911) Coleópteros nuevos del S.W. de Marruecos. Boletín de la Real Sociedad Española de Historia Natural 11: 299–304.

[B41] Escalera MM (1913) Una campaña entomológica en el Susy descripción de los coléopteros recogidos en ella. Trabajos del Museo de Ciencias Naturales (Serie Zoologica) 8: 1–56.

[B42] Escalera MM (1914) Los coleópteros de Marruecos. Trabajos del Museo Nacionál de Ciencias Naturales Série Zoológica (Madrid) 11: 1–553.

[B43] Escalera MM (1925) Especies de *Pachychila* y otros tenebriónidos nuevos de Marruecos. Boletín de la Real Sociedad Española de Historia Natural 25: 372–385.

[B44] Escalera MM (1940) Especies de *Sepidium* F. de Ifni (Col. Tenebrionidae). Eos, Revista Espaňola de Entomologia 13: 5–11.

[B45] Eschscholtz JF (1829) Zoologischer Atlas, enthaltend Abbildungen und Beschreibungen neuer Tierarten, während des Flottscapitains v. Kotzebue zweiter Reise um die Welt, auf der Russisch-Kaiserlichen Kriegsschlupp Predpriaetie in den Jahren 1823–1826 beobachtet. Drittes Heft. G. Reimer, Berlin, 18 pp. 10.5962/bhl.title.152182

[B46] Español F (1944) Nuevos datos para el conocimiento de los tenebrionidos (Col.) del Sahara. Eos, Revista Española de Entomología 20: 7–30.

[B47] Fabricius JC (1775) Systema entomologicae, systens insectorum classes, ordines, genera, species, adiectis synonymis, locis, descriptionibus, observationibus. Libraria Kortii, Flensburgi et Lipsiae, 832 pp. 10.5962/bhl.title.36510

[B48] Fabricius JC (1781) Species insectorum, exhibens eorum differentias specificas, synonyma auctorum, loca natalia, metamorphosis, adiecitis observationibus, descriptionibus. Tom I. Carol Ernest Bohnii, Hamburgi et Kilonii, 552 pp. 10.5962/bhl.title.36509

[B49] Fabricius JC (1787) Mantissa Insectorum sistens eorum species detectas, adiectis characteribus, genericis, differentiis specificis, emendationibus, observationibus. Tom 1. Christ. Gottl. Proft, Hafniae, 348 pp. 10.5962/bhl.title.11657

[B50] Fabricius JC (1792) Entomologica systematica emendata et aucta. Secundum classes, ordines, genera, species adjectis synonimis, locis, observationibus, descriptionibus. Tom I. Pars 1. Christ. Gottl. Proft, Hafniae, xx + 330 pp. 10.5962/bhl.title.36532

[B51] Fabricius JC (1798) Supplementum Entomologia systematica. Proft & Storch, Hafniae, [4] + 572 pp.

[B52] Fåhraeus (1870) Coleoptera Caffrariae, annis 1838–1845 a J. A. Wahlberg collecta. Heteromera descripsit. Öfversigt af Kongliga Vetenskaps-Akademiens Förhandlingar 27: 243–358.

[B53] Fairmaire L (1894) [new species description]. In Bulletin des séances et bulletin bibliographique de la Société entomologique de France. Séance du 28 novembre 1894: CCLII–CCLIII.

[B54] Fairmaire L (1871) Essai sur les Coléoptères de Barbarie. Annales de la Société entomologique de France 10: 369–404.

[B55] Fairmaire L (1882) [new taxa]. In: Fairmaire L, Lansberge V, Bourgeois J. Mission G. Révoil aux Pays Çomalis. Faune et Flore. Coléoptères recueillis par MG Révoil chez les Çomalis. Descriptions. Challamel Ainé, Paris, iv + 104 pp, 1 pl. [VI–1882].

[B56] Fairmaire L (1882b) Comptes-rendus des Séances de la Société Entomologique de Belgique: 3(16): XLII–LX.

[B57] Fairmaire L (1884) Diagnoses de Coléoptères de l’Afrique Orientale. Comptes-rendus des Séances de la Société Entomologique de Belgique 3(42): LXX–LXXVIII.

[B58] Fairmaire L (1887) Coléoptères des voyages de MG Revoil chez les Somâlis et dan l’intérieur du Zanguebar. Annales de la Société Entomologique de France 6: 69–186.

[B59] Fairmaire L (1888a) Énumération des Coléopteres recueillis par M. le Dr. Hans Schinz dans le sud de l’Afrique. Annales de la Société Entomologique de France 6: 173–202.

[B60] Fairmaire L (1888b) Coléoptères nouveaux de l’Afrique du Musée de Leyde. Notes from the Leyden Museum 10: 255–271.

[B61] Fairmaire L (1891a) Notes sur quelque Coléoptères de l’Afrique intertropicale et descriptions d’espèces nouvelles. Annales de la Société Entomologique de France 60: 231–274.

[B62] Fairmaire L (1891b) Coléoptères de l’Afrique Orientale. Comptes-rendus des Séances de la Société Entomologique de Belgique 4(20): CCLXXIX–CCCVII.

[B63] Fairmaire L (1893) Notes sur quelques Coléoptères des pays Somalis. Annales de la Société Entomologique de Belgique 37(4): 144–156.

[B64] Fairmaire L (1894) Coléoptères de l’Afrique intertropicale et Australe (deuxième note). Annales de la Société Entomologique de Belgique 38(6): 314–335.

[B65] Fairmaire L (1897) Coléoptères nouveaux de l’Afrique intertropicale et Australe (4e note). Annales de la Société Entomologique de France 66: 109–155. 10.5962/bhl.part.29501

[B66] Fairmaire L (1899a) La Faune entomologique du Delagoa. In: Junod, HA Missionnaire avec la collaboration du Prof. E Bugnion. Bulletin de la Société Vaudoise des Sciences Naturelles, XXXV: 162–188.

[B67] Fairmaire L (1899b) Matériaux pour la faune Coléoptèrique de la région Malagache (8e note). Annales de la Société Entomologique de Belgique 43: 511–558.

[B68] Fairmaire L (1901) Matériaux pour la faune Coléoptèrique de la région Malagache (11e note). Revue d’Entomologie 20(5/6): 101–248.

[B69] Ferrer J (1991) Rediscovery of type material of Gustav Johan Billberg (1815) in the Naturhistorika Riksmuseet, Stockholm (Coleoptera: Tenebrionidae). Annals of the Transvaal Museum 35(19): 279–283.

[B70] Ferrer J (1995) Contribution to the knowledge of the Tenebrionidae of Somalia (Coleoptera). Frustula Entomologica 18: 1–76.

[B71] Ferrer J (2000) *Trachynotideusdamarinus* Péringuey 1910, est transféré dans le genre *Somaticus* Hope 1840 (*sensu* Koch 1955). (Coleoptera: Tenebrionidae). Nouvelle Revue d’Entomologie 17(2): 145–146.

[B72] Ferrer J (2004a) Tenebrionidae (Coleoptera) de Namibia, avec descriptions de 12 espèces nouvelles. Mitteilungen aus dem Museum für Naturkunde in Berlin, Zoologische Reihe 80(2): 181–250. 10.1002/mmnz.4850800204

[B73] Ferrer J (2004b) Tenebriónidos nuevos o interesantes del Museo de Génova (Coleoptera). Annali del Musei Civico do Storia Naturale Giacomo Doria 96: 507–546.

[B74] Ferrer J (2009) The types of darkling beetles (Coleoptera: Tenebrionidae) described by Thunberg (1821, 1827) in Coleoptera Capensia and other papers, with taxonomic comments. Boletin Sociedad Entomológica Aragonesa 44: 111–129.

[B75] Ferrer J (2012) Contribución al concimiento del género *Trachynotus* Latreille: un caso de homonimia en el género Sepidium Fabricius (Coleoptera: Tenebrionidae: Pimeliinae). Boletin Sociedad Entomológica Aragonesa 51: 283–287.

[B76] Ferrer J Holston K (2009) Identities of *Tenebrio* Linnaeus types at Uppsala, and the resulting changes in old darkling beetle names (Insecta: Coleoptera: Tenebrionidae). Zootaxa 2308: 29–42. 10.11646/zootaxa.2359.1.7

[B77] Ferrer J Evanno C Evanno A (2010) Description of a new species of *Tarsocnodes* Gebien Coleoptera, Tenebrionidae, Molurini from Congo. Boletin de la Sociedad Entomológica Aragonesa 47: 195–198.

[B78] Forskål P (1775) Descriptiones animalium avium, amphibiorum, piscium, insectorum, vermium quae in itinere orientali observavit. Mölleri, aulae Typographi, Hauniae, 232 pp. 10.5962/bhl.title.2154

[B79] Gahan CJ (1896) On Coleoptera from Aden and Somaliland. Annals and Magazine of Natural History 6(18): 448–461. 10.1080/00222939608680485

[B80] Gahan CJ (1900) [new species description]. On a Collection, of Insects and Arachnids made in 1895 and 1897, by C V A Peel, FZS, in Somaliland, with Descriptions of new Species. By CVA Peel, FZS, EF Austen, FA. Dixey, MA, MD, Herbert Drece, FLS, FZS, CJ Gahax, MA, Gilbert J Arrow, R McLachlan, FRS., Malcolm Burr, FZS, and RI Pocock. Proceedings of the General Meetings for Scientific Business of the Zoological Society of London, 1259 pp.

[B81] Gebien H (1910a) Diagnosen der von Dr. Sheffield Neave im südlichen Kongo-Gebiet gesammelten Tenebrioniden nebst Beschreibungen neuer Arten aus Deutsch-Ostafrika. Annales de la Société entomologique de Belgique 54: 144–182.

[B82] Gebien H (1910b) Pars 15. Tenebrionidae I [pp. 1–166]. In: Schenkling S (Ed.) Coleopterorum Catalogus Volumen XVIII. W. Junk, Berlin, 742 pp.

[B83] Gebien H (1913) Coleoptera, Tenebrionidae. Wissenschaftliche ergebnisse der Deutschen Zentral-Africa-Expedition, 1907–1908: unter Führung Adolf Friedrichs, Herzogs zu Mecklenburg. Band IV: 57–79.

[B84] Gebien H (1920) Käfer aus der Familie Tenebrionidae gesammelt auf der “Hamburger deutsch-südwestafrikanischenStudienreise 1911”. Hamburgische Universität Abhandlungen aus der Auslandskunde Band 5. Reihe C Naturwissenschaften Band 2. Hamburg: L. Friederichsen & Co., 168 pp.

[B85] Gebien H (1937a) Katalog der Tenebrioniden (Col. Heteromera). Teil I. Pubblicazioni del Museo Entomologico «Pietro Rossi» 2: 505–883.

[B86] Gebien H (1937b) Ueber neue Tenebrioniden Ostafrikas aus den Sammlungen des Museo Civico di Storia Nautrale di Trieste. Atti del Museo Civico di Storia Naturale di Trieste 14(2): 21–56.

[B87] Gebien H (1938a) Die Tenebrioniden (ColeopteraHeteromera) der Namibwüste in Südwestafrika. Abhandlungen herausgegeben vom Naturwissenschaftlichen Verein zu Bremen 30: 20–107.

[B88] Gemminger M (1870) [new names]. In: Harold E von (Ed.) Geänderte Namen.Coleopterologische Hefte 6: 119–124.

[B89] Generani M Scaramozzino PL (2000) Australian Hymenoptera in the Spinola collection: a list of species [pp. 231–243]. In: Austin AD, Dowton M (Eds) Hymenoptera: evolution, biodiversity and biological control. CSIRO Publishing, Collingwood, xi + 468 pp.

[B90] Germar EF (1823) Species insectorum novae aut minus cognitae, descriptionibus illustratae. Volumen Primum. Coleoptera. J. C. Hendelii et filii, Halae, 624 pp. 10.5962/bhl.title.130964

[B91] Gerstaecker A (1854) [new species description]. Bericht über die zur Bekanntmachung geeigneten Verhandlungen der Königl. Preuss. Akademie der Wissenschaften zu Berlin: 530–534.

[B92] Gerstaecker A (1871) Beitrag zur Insektenfauna von Zanzibar. III. Coleoptera. Archiv für Naturgeschichte 37(1): 42–86.

[B93] Gerstaecker A (1884) Bestimmung der von Herr Dr. G. A. Fischer während seiner Reise nach dem Massai-Land gesammelten Coleopteren. Jahrbuch der Hamburgischen Wissenschaftlichen Anstalten zu Hamburg für 1883: 43–63.

[B94] Gestro R (1878) Diagnosi di alcune specie nuove di Coleotteri dell’Abssinia e del paese dei Somali. Annali del Museo Civico de Storia Naturale 13: 318–322.

[B95] Gestro R (1883) Appunti sinonimici. Annali del Museo Civico di Storia Naturale di Genova 20: 302–306.

[B96] Gestro R (1892) Di alcuni Coleotteri raccolti nel paese dei Somali. Annali del Museo Civico di Storia Naturale di Genova 32: 747–790.

[B97] Gestro R (1895) Esplorazione del Giuba e dei suoi affluenti compiuta dal Cap P. Bottego durante gli anni 1892–93 sotto gli auspicii Della Società Geografica Italiana. Risultati Zoologici. XVI Coleotteri. Tipografia R. Istituto Sordo-Muti, Genova, 254 pp. 10.5962/bhl.title.49163

[B98] Gestro R (1898) Contribuzione allo studio dei Sepidiini. Annali del Museo Civico di Storia Naturale di Genova 34: 512–158.

[B99] Gmelin JF (1790) Caroli a Linné Systema Naturae per regna tria naturae, secundum classes, ordines, genera, species, cum characteribus, diﬀerentiis, synonymis, locis. Editio decima tertia, aucta, reformata. Tom. I. Pars IV. Georg Emanuel Beer, Lipsiae, 1517–2224.

[B100] Gridelli E (1939a) ColeopteraStaphylinidae, Diversicornia, Heteromera, Lamellicornia, Chrysomelidae (Partim). Missione Biologica nel Paese dei Borana. Volume secondo Raccolte Zoologiche Parte prima Reale Accademia d’Italia, Centro Studi per l’Africa Orientale Italiana, Roma 2: 85–315.

[B101] Gridelli E (1939b) Coleotteri dell’Africa Orientale Italiana. 11 Contributo. Materiali per lo studio della Fauna Eritrea raccolti nel 1901–03 dal Dott. Alfredo Andreini. Memorie della Società Entomologica Italiana 18: 219–258.

[B102] Gridelli E (1953) Catalogo ragionato delle specie di Coleotteri Tenebrionini dell’Arabia. Atti del museo civico di storia naturale di Trieste 19: 3–73.

[B103] Guérin-Méneville FE (1831) Iconographie du règne animal de G. Cuvier, ou representation d’après la nature de Vune des espèces les plus remarquables et souvent non encore figurées, de chaque genre d’animaux. Avec un texte descriptif mis au couraní de la science. Ouvrage pouvant servir ďatlas à tous les traités de zoologie. II. Planches des animaux invertébrés. Insectes. [1829–1838]. 10.5962/bhl.title.6255

[B104] Guérin-Méneville FE (1834) Matériaux pour une classification des mélasomes. (Extraits ďune monographie de cette famille). Magasin de Zoologie 1: 1–39.

[B105] Guérin-Méneville FE (1844) Insectes Magasin de Zoologie, D’ Anatomie Comparee et de Paleontologie, Recueil, 377 pp.

[B106] Guérin-Méneville FE (1845) Description de quelques-uns des Insectes les plus remarquable découverts par M. A. Delegorcue dans les pays des Boschimans, des Ama Zoulous, des Massilicatzi et au Port Natal, pendant les années 1838, 39 , 40, 41, 42, 43 et 44. Revue Zoologique par La Société Cuvierienne VIII: 283–286.

[B107] Guérin-Méneville FE (1858) Description de deux coléoptères du genre *Sepidium* , dont l’un est pentamère et paraít étre le male, et l’autre est hétéromère. Revue de Zoologie Pure et Appliquée (2) 10: 127–129.

[B108] Haag-Rutenberg G (1871a) Beiträge zur Familie der Tenebrioniden (II. Stück). Coleopterologische Hefte VII: 21–111.

[B109] Haag-Rutenberg G (1871b) Beiträge zur Familie der Tenebrioniden (III. Stück). Coleopterologische Hefte VIII: 29–113.

[B110] Haag-Rutenberg G (1873) Beiträge zur Familie der Tenebrioniden (IV. Stück). Coleopterologische Hefte XI: 1–49.

[B111] Haag-Rutenberg G (1875) Beiträge zur Familie der Tenebrioniden (V. Stück). Coleopterologische Hefte XIV: 67–92.

[B112] Haag-Rutenberg G (1877) [new species description] In: Vincenz G (Ed.) Zur Käfer-Fauna Central-Afrikas.Gesellschaft in Wien, XXVII, 501–522.

[B113] Haag-Rutenberg G (1879) Fernere Nachträge zu den Heteromeren-Monographien der Moluriden, Eurychoriden und Adesmiiden. Deutsche Entomologische Zeitschrift 23: 289–296. 10.1002/mmnd.48018790217

[B114] Harold E (1877) Coleopterorum species novae. Mittheilungen der münchner entomologischen Verein 1: 97–111.

[B115] Harold E (1878) Diagnosen neuer Coleopteren aus dem innern Africa. Mittheilungen der münchner entomologischen Verein 2: 99–111.

[B116] Herbst JFW (1799) Natursystem aller bekannten in- und ausländischen Insekten als eine Fortsetzung der von Büffonschen Naturgeschichte: Nach dem System des Ritters Carl von Linné bearbeitet von Carl Gustav Jablonsky, fortgesetzt von Johann Friedrich Wilhelm Herbst. Berlin, I–XVI.

[B117] Hesse AJ (1935) Scientific results of the Vernay-Lang Kalahari Expedition, March to September, 1930. Tenebrionidae (Coleoptera). Annals of the Transvaal Museum 16: 525–579.

[B118] Hope FW (1840) “*Somaticus* Hope”. In: Bridgewater JC. The Coleopterist’s Manual. 3. London, 191 pp.

[B119] ICZN (1999) International Code of Zoological Nomenclature, Fourth Edition, adopted by the International Union of Biological Sciences. International Trust for Zoological Nomenclature, London, xxix + 306 pp.

[B120] Irish J (1985) Zoological types in the State Museum. Cimbebasia 7: 108–132.

[B121] Karsch F (1881) Die Käfer der Rohlfs’schen Afrikanischen Expedition 1878–79. Berliner Entomologische Zeitschrift 25: 41–50. 10.1002/mmnd.18810250108

[B122] Kaszab Z (1963) Angaben zur Kenntnis der Tenebrioniden des Tschadsee-Gebietes, nebst einer Revision der afrikanischen Mesomorphus-Arten (Coleoptera). Revue de zoologie et de botanique africaines 67: 341–387.

[B123] Kaszab Z (1972) The scientific results of Hungarian Zoological Expedition to Tanganyika. 15. Coleoptera: Tenebrionidae. Annales historico-naturales Musei nationalis hungarici 63: 225–238.

[B124] Kaszab Z Pinheiro MFV (1972) Uma nova especie de *Sepidium* (Coleoptera, Tenebrionidae) em Portugal. Eine neue Sepidium Art (Coleoptera, Tenebrionidae) aus Portugal. Estudos e Divulgaçao Téchnica (C) Entomologia forestall 1972: 5–17.

[B125] Kirby W (1819) A century of insects, including several new genera described from his cabinet. The Transactions of the Linnean Society of London 12 [1817] (2): 375–482, pls. 21–23. 10.1111/j.1095-8339.1817.tb00239.x

[B126] Kirby WF (1885) Coleoptera. In: Bell FJ (Ed.) The Zoological Record for 1884; being volume the twenty-first of the record of zoological literature.John van Voorst, London, 14–125.

[B127] Kirchsberg O (1877) *Vietamillingenii* nov. spec. und *Arthrodeisarabicus* nov. spec. Deutsche entomologische Zeitschrift 21: 203–204. 10.1002/mmnd.4800210130

[B128] Klug JCF (1835) Insekten. In: Nordmann A Erman AG Klug JFC (Eds) Verzeichniss von Thieren und Pflanzen, welche auf enier Reise um die Erde gesammelt wurden.Georg Reimer, Berlin, 27–50.

[B129] Koch C (1951) Die Tenebrioniden des südlichen Afikas VII *Arturium* nov. gen. Molurinorum ex aff *Phrynocolus* Lac. Atti della Società Italiana di Scienze Naturali 90: 89–96.

[B130] Koch C (1952) Die Tenebrioniden des südlichen Afrikas XIII Vorstudien zu einer Monographie der Molurini, 3. (Col. Tenebrionidae). Entomologische Arbeiten 3: 214–349.

[B131] Koch C (1953a) Die Tenebrioniden des südlichen Afrikas XIV Über einige neue Molurini aus dem Ungarischen Naturwissenschaftlichen Museum zu Budapest (Vorarbeiten zu einer Monographie der Molurini, 4. Annales historico-naturales Musei nationalis hungarici 44 (series nova 3): 137–181.

[B132] Koch C (1953b) The Tenebrionidae of southern Africa XVII Contributions to the fauna of Angola. Publicações Culturais da Companhia de Diamantes de Angola 16: 61–96.

[B133] Koch C (1953c) The Tenebrionidae of Southern Africa XXIV. Vorläufige Beschreibung neuer Tenebrioniden des Südlichen Africas aus der Sammlung der Universität Lund. Lund University Arsskrift 49: 1–24.

[B134] Koch C (1953d) The Tenebrionidae of southern Africa III Tenebrionidae from a nest of Tatera. Revue de Zoologie et de Botanique Africaines 47: 1–30.

[B135] Koch C (1954a) Die Tenebrioniden des südlichen Afrikas XIX Zwei neue Distretus (Perdistretus) aus dem Belgischen Congo. Annales du Musée du Congo belge 1: 435–439.

[B136] Koch C (1954b) The Tenebrionidae of southern Africa XXVI New Port. East African species collected by Dr A J Barbosa. Revista da faculdade de ciencias, Universidade de Lisboa. Serie C. Ciencias Naturais 3: 239–310.

[B137] Koch C (1955) Monograph of the Tenebrionidae of southern Africa Vol I (Tentyriinae, MoluriniTrachynotina: Somaticus Hope). Transvaal Museum Memoir 7, 242 pp.

[B138] Koch C (1956) Die Tenebrioniden des Südlichen Afrikas – XXXVI Neue *Melanolophus* (Molurini) aus dem Museum Triest. Atti del Museo Civico di Storia Naturale di Trieste 20: 170–176.

[B139] Koch C (1958) Tenebrionidae of Angola. Publicacões Culturais da Companhia de Diamantes de Angola 39: 11–231.

[B140] Koch C (1959) Erster taxonomischer Beitrag zur Kenntnis der Tenebrioniden Somalis. Entomologischen Arbeiten aus dem Museum G. Frey Tutzing bei München 10: 568–596.

[B141] Koch C (1960) Dritter taxonomischer Beitrag zur Kenntnis der Tenebrioniden Somalias. Memorie della Società Entomologica Italiana 38: 257–268.

[B142] Koch C (1962a) Analysis of the Madagascan components of the subfamily Tentyriinae (Tenebrionidae, Coleoptera) with revisions of the generic systematics of the Asidini from Africa south of the Sahara and the African, Asiatic and Palaearctic Epitragina of Tentyriini. Mémoires de l’institut de la scientifique de Madagascar 13: 1–146.

[B143] Koch C (1962b) The Tenebrionidae of Southern Africa XXXII New psammophilous species from the Namib Desert. Annals of the Transvaal Museum 24: 107–159.

[B144] Koch C (1962c) Vierter taxonomischer Beitrag zur Kenntnis der Tenebrioniden Somalias: über die von Prof G Scortecci 1953 und 1957 in der Migiurtinia Provinz gesammelten Arten. Atti della Società italiana di scienze naturali e del Museo civile di storia naturale 101: 237–270.

[B145] Koch C (1965) Missione 1962 del Prof Giuseppe Scorecci nell’Arabia meridionale Coleotteri Tenebrionidae Includendo materiale di viaggi in Arabia del Sig G Popov (1962) e del Dr G Benardelli (1962–63. Atti della Società italiana di scienze naturali e del Museo civile di storia naturale 104: 99–154.

[B146] Koch C (1969) Sechster taxonomischer Beitrag zur Kenntnis der Tenebrioniden Somalias Abhandlungen über die tropisch-xerophilen Molurini-Gattungen *Phrynocolus* und *Phrynophanes*, sowie Untergattung *Somalarabes* von *Psammophanes*. Entomologische Arbeiten aus dem Museum G Frey Tutzing bei München 20: 1–35.

[B147] Kocher L (1958) Description de nouveaux coléoptères du Maroc. Bulletin de la Société de Sciences Naturelles et Physiques du Maroc 48: 107–113.

[B148] Kolbe HJ (1883) Neue Coleoptera von Westafrika. Berliner entomologische Zeitschrift 27: 15–36. 10.1002/mmnd.18830270105

[B149] Kolbe HJ (1886) Neue afrikanische Coleoptera des Berliner zoologischen Museums. In: Karsch F Entomologishe Nachrichten 11: 289–301.

[B150] Kolbe HJ (1891) Aufzählung der von Herrn Dr. Hans Meyer im Jahre 1889 im Gebiete des Kilimandscharo- und Ugueno-Gebirges gesammelten Coleopteren Stettiner Entomologische Zeitung 52: 18–36.

[B151] Kolbe HJ (1904) Über einige interessante Lamellicornier und Tenebrioniden Afrikas. Berliner entomologische Zeitschrift 49: 282–302.

[B152] Kraatz G (1897) Zwei neue ansehnliche *Psammodes* – Arten aus Ostafrica. Deutsche Entomologische Zeitschrift 1897 (Heft 1): 46–48. 10.1002/mmnd.48018970108

[B153] Kulzer H (1960) Einige neue Tenebrioniden (Col.). Entomologische Arbeiten aus dem Museum G. Frey Tutzing bei München 11: 304–432.

[B154] Kulzer H (1963) Verzeichnis des Typenmaterialsder Tenebrionidensammlung des Museums G. Frey. Entomologische Arbeiten aus dem Museum G. Frey Tutzing bei München 14: 375–599.

[B155] Kwieton E (1978) Espèces nouvelles des genres *Adesmia* Fisch., *Pimelia* Sol. et *Vieta* (Col. Tenebrionidae). Bulletin de la Société Entomologique de Mulhouse 1978 8–12.

[B156] Kwieton E (1980) Synopsis des espèces du genre *Sepidium* F. d’Algerie et de Tunisie (Col., Tenebrionidae). Annotationes Zoologicae et Botanicae, 138: 1–19.

[B157] Lacordaire JT (1859) Histoire naturelle des insectes. Genera des Coléoptères ou exposé méthodique et critique de tous les genres proposés jusqu’ici dans cet ordre d’insectes. Tome cinquième contenant les familles des ténébrionides, cistélides, nilionides, pythides, mélandryides, lagriides, pédilides, anthicides, pyrochroïdes, mordellides, rhipiphorides, stylopides, meloïdes et oedémérides. Librairie Encyclopédique de Roret, Paris, Première partie (1–400), Deuxième partie (401–750). [1859 (title page); 27 Jun 1859 (Acad. Sci. France); 16 Jul 1859 (Bibliogr. France 1859)].

[B158] Laporte FLN de Caumont de Castelnau (1840) Histoire naturelle des insectes coléoptères; avec une introduction renfermant l’anatomie et la physiologie des animaux articulés, par M Brullé. Tome deuxième. P Duménil, Paris, 563 pp.

[B159] Latreille PA (1802) Histoire naturelle, générale et particulière des crustacés et des insectes. Ouvrage faisant suite à l’histoire naturelle générale et particulière, composée par Leclerc de Buffon, et rédigée par CS Sonnini, membre de plusieurs sociétés savantes. Familles naturelles des genres. Tome troisième. F Dufart, Paris, xii + 13–467 + [1] pp. [An X (title page, = 1802); Nov 1802 (Evenhuis 1997)]. 10.5962/bhl.title.15764

[B160] Latreille PA (1828) Rhynchophores ou porte-bec. In: Bory de Saint-Vincent JBGM (Ed.) Dictionnaire classique d’histoire naturelle. Tome quatorzième. Pla-Roy. Rey & Gravier, Baudouin Frères, Paris, 584–603. [Sep 1828 (title page)].

[B161] Lesne P (1922) Bostrychides, Clérides, Sphindides, et Ténébrionides. In: Rothschild MB Voyage de M. le baron Maurice de Rothschild en Éthiopie et en Afrique Orientale Anglaise (1904–1905). Résultats scientifiques. Animaux articulés. Deuxième partie. Imprimerie Nationale, Paris, 483–1041.

[B162] Lighton JRB (1987) Cost of tokking: the energetics of substrate communication in the tok-tok beetle, *Psammodesstriatus*. Journal of Comparative Physiology A 157: 11–20. 10.1007/BF00702723

[B163] Linnaeus C (1760) Fauna Sueciae sistens animalia Sueciae regni: Mammalia, Aves, Amphibia, Pisces, Insecta, Vermes, distributa per classes, ordines, genera et species, cum diferentiis specierum, synonymis, auctorum, locis natalium, descriptionibus insectorum; editio altera, auctiora “1761”, Laurentii et Salvi, Stockholmiae, 48 + 578 pp., 2 pl. 10.5962/bhl.title.46380

[B164] Louw S (1979) A partial revision of the subtribes Oxurina and Hypomelina (Coleoptera: Tenebrionidae: Molurini). Cimbebasia 5: 95–177.

[B165] Louw S (1980) Synonymy of *Argenticrinishaackei* Louw and *Psammodeslossowi* Koch (Coleoptera: Tenebrionidae: Molurini). Cimbebasia series A 55: 216–217.

[B166] Löbl I Smetana A (2008) Errata. In: Löbl I, Smetana A (Eds) Catalogue of Palaearctic Coleoptera. Volume 5. Tenebrionoidea. Apollo Books, Stenstrup, 21–27. [publ. 15 Apr 2008 (verso of title page)].

[B167] Lucas R (1920) Catalogus alphabeticus generum et subgenerum Coleopterorum orbis terrarum totius. R. Stricker, Berlin, xxvi + 696 pp.

[B168] Mal N (1984) Une espèce de *Sepidium* afine à *S.bidentatum* Solier, et description d´une espèce nouvelle de Portugal. L´Entomologiste 40: 193–204.

[B169] Mal N (1986a) Additions au genere *Vietomorpha* Fairmaire, 1887 (ColeopteraTenebrionidae), Monitore Zoologico Italiano. Supplemento, 21: 11–24. 10.1080/03749444.1986.10736705

[B170] Mal N (1986b) Description de deux espèces nouvelles du genre “*Sepidium* ” Fabricius, 1755 (ColeopteraTenebrionidae). Atti del Museo Civico di Storia Naturale di Trieste 39: 151–157.

[B171] Mal N (1990) Description d’une espèce nouvelle du genre *Sepidium* Fabricius, 1775 (Coleoptera, Tenebrionidae. Lambillionea 90: 64–67.

[B172] Mal N (2005) Description d’une espèce nouvelle du genre *Physophrynus* Fairmaire, 1882 (Coleoptera: Tenebrionidae: Molurini). Annales Zoologici 55: 9–10. 10.3161/0003454053642211

[B173] Merkl O Grabant A Soltész Z (2015) Type Catalogue of Darkling Beetles (Tenebrionidae) preserved in the Hungarian Natural History Museum. Hungarian Natural History Museum, Budapest, 735 pp.

[B174] Müller CL (1887) Vierzehn neue Heteromeren von Bradshaw im Zambesi-Gebiete aufgefunden, mit Abbildungen von van de Poll. Tijdschrift voor entomologie 30: 297–308.

[B175] Matthews EG Lawrence JF Bouchard P Steiner WE Ślipiński SA (2010) 11.14 Tenebrionidae Latreille, 1802. In: Leschen RAB Beutel RG Lawrence JF (Eds) Handbook of zoology.A natural history of the phyla of the animal kingdom. Vol. IV. Arthropoda: Insecta. Walter de Gruyter, Berlin, 574–659. 10.1515/9783110911213.574

[B176] Ohl M (2012) The primary types of Mantispidae (Neuropterida)in the Museum fur Naturkunde, Berlin – An annotated catalogue. Zoosystematics and Evolution 88: 97–124. 10.1002/zoos.201200010

[B177] Olivier AG (1795) Entomologie, ou histoire naturelle des insectes, avec leur caractères génériques et spécifiques, leur description, leur synonymie, et leur figure enluminée. Coléoptères. Tome troisième. De Lanneau, Paris, 557 pp.

[B178] Pallas PS (1781) Icones Insectorum praesertim Russiae sibiriaeque peculiarum quae collegit et descriptionibus illustravit. Wolfgangi Waltheri, Erlangae 4, 104 pp. 10.5962/bhl.title.15809

[B179] Pascoe FP (1866) Notices of new or little-known genera and species of Coleoptera. Journal of Entomology 1866: 443–492.

[B180] Penrith M-L (1986) Revision of the genera *Bombocnodulus* Koch and *Brinckia* Koch (Coleoptera: Tenebrionidae: Molurini). Journal of the Entomological Society of Southern Africa 49: 55–85.

[B181] Penrith M-L (1987) Revision of the genus *Tarsocnodes* Gebien (Coleoptera: Tenebrionidae: Molurini), and a description of a monotypical genus from the Kalahari. Cimbebasia Series 7: 236–270.

[B182] Péringuey LA (1885) First contribution to the South-African Coleopterous Fauna. Transactions of the South African philosophical Society 3: 74–149. 10.1080/21560382.1881.9526176

[B183] Péringuey LA (1886) Second contribution to the South-African Coleopterous Fauna. Transactions of the South African Philosophical Society 4: 67–19. 10.1080/21560382.1884.9526202

[B184] Péringuey LA (1892) Third contribution to the South-African Coleopterous Fauna. Transactions of the South African philosophical Society 6: 1–94. 10.1080/21560382.1889.9526255

[B185] Péringuey LA (1896) Descriptions of new genera and species of Coleoptera from South Africa, chiefly from Zambezia. The Transaction of the Entomological Society of London 1896: 149–189. 10.1111/j.1365-2311.1896.tb00961.x

[B186] Péringuey LA (1899) Fifth contribution to the South-African Coleopterous Fauna. Annals of the South Africa Museum 1: 240–330.

[B187] Péringuey LA (1904) Sixth contribution to the South African Coleopterous fauna. Annals of the South African Museum 3: 167–300.

[B188] Péringuey LA (1908) Tenebrionidae und Curculionidae. Denkschriften der Medicinisch-Naturwissenschaftlichen Gesellschaft zu Jena 13: 393–424.

[B189] Pierre F (1979) Les Tenebrionidae du Djebel Marra (Soudan) et notes sur quelques particularites de leur morphologie (Col.). Bulletin de la Société Entomologique de France 84: 4–10.

[B190] Quedenfeldt G (1885) Verzeichniss der von Herrn Major a. D. von Mechow in Angola und am Quango-Strom 1878–1881 gesammelten Tenebrioniden und Cisteliden. Berliner entomologische Zeitschrift 29: 1–38. 10.1002/mmnd.18850290106

[B191] Quedenfeldt G (1888) Beiträge zur Kenntniss der Koleopteren-Fauna von Central-Afrika nach den Ergebnissen der Lieutenant Wissman’schen Kassai-Expedition 1883 bis 1886. Berliner entomologische Zeitschrift 32: 155–219.

[B192] Quensel K (1806) Schoenherr CJ Synonymia Insectorum, oder: Versuch einer Synonymie aller bisher bekannten Insecten; nach Fabricii Systema Eleutheratorum geordnet. Erster Band. Eleutherata oder Käfer. Heinr. A. Nordstrom, Stockholm, xxii + 293 pp. 10.5962/bhl.title.66107

[B193] Rafinesque CS (1815) Analyse de la nature ou tableau de l’univers et des corps organisés. Rafinesque, Palermo, 224 pp. 10.5962/bhl.title.106607

[B194] Reiche LJ (1850) Entomologie. In: Ferret A, Galinier JG (Eds) Voyage en Abyssinie 1839–43, Voyage en Abyssinie dans les provinces du Tigré, du Samen et de l’Amhara. Paulin, Paris, 259–471, 13 pls.

[B195] Reitter E (1914) Bestimmungs-Tabelle der Tenebrioniden-Abteilung der Sepidiini. Deutsche entomologische Zeitschrift 1914: 381–392. 10.1002/mmnd.48019140404

[B196] Robiche G (2013) Note sur le genre *Psammodes* Kirby, 1818 au Mozambique et descriptions de nouvelles espèces appartenant aux genres *Psammophanes* Lesne, 1922 et *Dichtha* Haag-Rutenberg, 1871 (Coleoptera, Tenebrionidae). Lambillionea 113: 155–166.

[B197] Rotrou M (1943) Description d’une nouvelle espèce de *Sepidium* (Coléoptère) d’Algérie. Entomologiste 40: 193–204.

[B198] Rye EC (1873) Insecta. Coleoptera. In: Newton A (Ed.) The Zoological Record for 1871; being the volume eighth of the record of zoological literature.John van Voorst, London, 222–329.

[B199] Sahlberg F (1903) Coleoptera Numido-Punica. Ofversigt Finska Forhandlingar XLV: 1–70.

[B200] Scherer G (1992) Die Sektion Coleoptera der Zoologischen Staatssammlung München. Spixiana. Zeitschrift für Zoologie, Supplement 17: 61–71.

[B201] Schuster A (1928b) Neue Tenebrioniden aus Cyrenaica (IV). Bollettino della Società Entomologica Italiana 60: 122–124.

[B202] Schuster A Gebien H (1938) Tenebrioniden (Col.) aus Arabien. Entomologische Blätter 34: 49–62.

[B203] Solier AJJ (1843) Essai sur les collaptérides de la tribu des Molurites. Imprimerie Royale, Turin, 127 pp. [4 pls.] [extract of Memorie della Reale Accademia delle Scienze di Torino (2)6 [1844]: 213–339].

[B204] Thunberg CP (1787) Museum Naturalium Academiae Uppsaliensis. Cujus partem quartam. Publico examini subjicit P. Bjerkén. Joh. Edman, Uppsaliae, [2] + 43–58 pp.

[B205] Thunberg CP (1791) Dissertatio entomologica novas insectorum species. 6. (Dissertatio resp. A. J. Lagus). Joh. Edman, Uppsaliae, i–iv, 107–130.

[B206] Thunberg CP (1799) De Brachycero, tractatus entomologicus. Nova Acta Regiae Societatis Scientiarum Upsaliensis 6: 11–37.

[B207] Thunberg CP (1813) Coleoptera Rostrata Capensia. Mémoires de l’Académie Impériale des Sciences de St. Pétersbourg (5. Sér.) 4 [1811]: 376–400.

[B208] Thunberg CP (1821) *Opatrum* Insecti genus. Reg. Academiae Typographi, Uppsaliae, i–v, 27–34.

[B209] Viñolas A Caballero-López B Masó G (2017) The collection of type specimens belonging to the subfamily Pimeliinae (Coleoptera, Tenebrionidae) in the Natural Sciences Museum of Barcelona, Spain. Arxius de Miscellània Zoològica 15: 30–92. 10.32800/amz.2017.15.0030

[B210] Waterhouse CO (1885) On the Insects collected on Kilima-njaro. Journal of Zoology 53: 230–235. 10.1111/j.1096-3642.1885.tb02900.x

[B211] Westwood JO (1875) XIV. Descriptions of new Heteromerous Coleoptera. Ecological Entomology 23: 223–232. 10.1111/j.1365-2311.1875.tb01909.x

[B212] Wiedemann CRW (1823) Zweihundert neue Käfer von Java, Bengalen, und dem Vorgebirge der Guten Hoffnung. Zoologisches Magazin 2: 3–133.

[B213] Wilke S (1921) Die Molurinen-Gattung *Phrynocolus* Lac. (Col., Tenebr.). Archiv für Naturgeschichte 87: 161–174.

[B214] Wilke S (1922) Die *Phrynocolus*-Arten des Genueser zoologischen Museums. (Col. Tenebr.). Berliner entomologische Zeitschrift 1922: 381. 10.1002/mmnd.192219220409

[B215] Zimsen E (1964) The type material of I.C. Fabricius. Munksgaard, Copenhagen, 656 pp.

